# *Rhinochelys amaberti* Moret (1935), a protostegid turtle from the Early Cretaceous of France

**DOI:** 10.7717/peerj.4594

**Published:** 2018-04-10

**Authors:** Isaure Scavezzoni, Valentin Fischer

**Affiliations:** Evolution and Diversity Dynamics Lab, Université de Liège, Liège, Belgium

**Keywords:** Albian, Mesozoic, Testudinata, Vallon de la Fauge, Cambridge Greensand, Cryptodira

## Abstract

Modern marine turtles (chelonioids) are the remnants of an ancient radiation that roots in the Cretaceous. The oldest members of that radiation are first recorded from the Early Cretaceous and a series of species are known from the Albian-Cenomanian interval, many of which have been allocated to the widespread but poorly defined genus *Rhinochelys*, possibly concealing the diversity and the evolution of early marine turtles. In order to better understand the radiation of chelonioids, we redescribe the holotype and assess the taxonomy of *Rhinochelys amaberti* Moret (1935) (UJF-ID.11167) from the Late Albian (*Stoliczkaia dispar* Zone) of the Vallon de la Fauge (Isère, France). We also make preliminary assessments of the phylogenetic relationships of Chelonioidea using two updated datasets that widely sample Cretaceous taxa, especially *Rhinochelys*. *Rhinochelys amaberti* is a valid taxon that is supported by eight autapomorphies; an emended diagnosisis proposed. Our phylogenetic analyses suggest that *Rhinochelys* could be polyphyletic, but constraining it as a monophyletic entity does not produce trees that are significantly less parsimonious. Moreover, support values and stratigraphic congruence indexes are fairly low for the recovered typologies, suggesting that missing data still strongly affect our understanding of the Cretaceous diversification of sea turtles.

## Introduction

‘Turtles’ (Testudinata) is a diversified group of diapsid reptiles (Bever et al., 2015) with several terrestrial, marine, and fresh-water species. Modern marine turtles (Chelonioidea) are divided into two clades (i.e. leathery-shelled turtles and hard-shelled turtles), which are supported by morphological and embryological characters (Wyneken, 2001) and are part of a wider radiation, whose oldest fossil record dates from the Barremian of Columbia (Cadena & Parham, 2015). A series of extinct chelonioids, known collectively as as Protostegidae (Hirayama, 1997), were present during the Early Cretaceous. Chelonioids were diversified during the Late Cretaceous, with the presence of at least five distinct clades at the end of the Cretaceous (Nicholson et al., 2015; Cadena & Parham, 2015). These Late Cretaceous taxa exhibit a wide range of morphologies such as the gigantic taxa *Archelon, Protostega* and *Atlantochelys*, and taxa with highly unusual cranial architecture such as *Alienochelys* and *Ocepechelon*  (Hay, 1908; Zangerl, 1953; Bardet et al., 2013; De Lapparent de Broin et al., 2014). The diversity and disparity of Late Cretaceous chelonioid suggests an earlier diversification, during the Early Cretaceous.

A series of species have been described from ‘middle’ Cretaceous strata, most of them being assigned to the widespread genus *Rhinochelys*. However, *Rhinochelys* is a poorly defined, likely waste-basket taxon, in dire need of revision (Collins, 1970; Hirayama, 1997; Hooks, 1998). As a result, the diversity of Early Cretaceous chelonioid and the nature and tempo of their radiation are poorly understood. To contribute to this wide issue, we redescribe the holotype of *Rhinochelys amaberti* Moret (1935) from the Upper Albian of France, which has never been re-assessed since 1935. We also describe a series of previously unreported chelonioid skulls originating from the lower part of the Cambridge Greensand (Upper Albian) of the United Kingdom (Collins, 1970; Hirayama, 1997; Hooks, 1998). We then evaluate the relationships of chelonoids and the monophyly of *Rhinochelys* using two updated matrices  Bardet et al. (2013) and Cadena & Parham (2015).

## Material & Methods

### Geography and geology of the holotype of *Rhinochelys amaberti*

**Figure 1:**
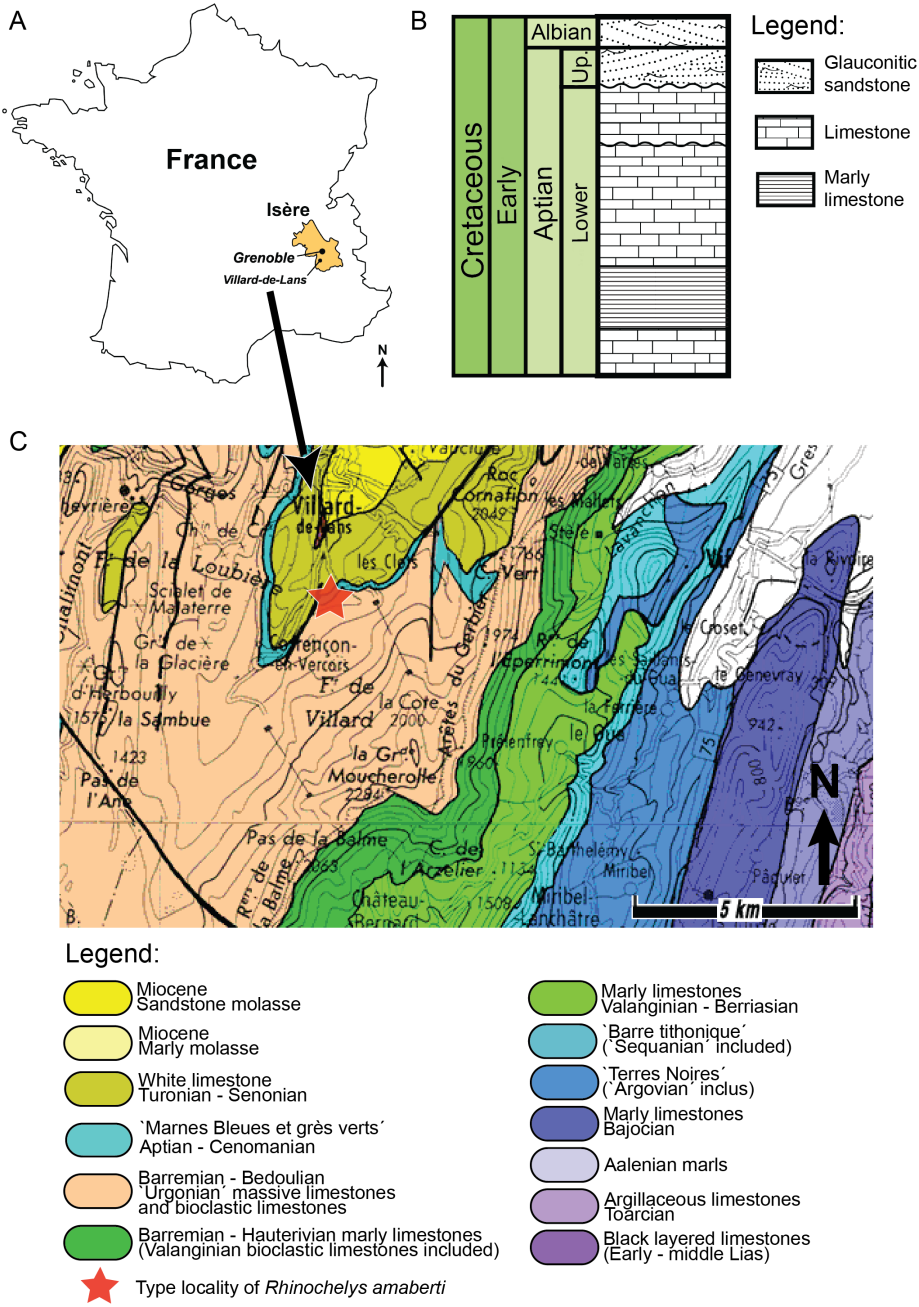
(A) Schematic map of France positioning Grenoble and Villard-de-Lans. (B) Stratigraphic log of northern Vercors representing Aptian-Albian deposits. Modified from Wilpshaar, Leereveld & Visscher (1997). (C) Simplified geological map from BRGM (1967) of western Vercors at the latitude of Villard-de-Lans (Gidon, 1977). Legend and map modified from Gidon (1977).

**Figure 2:**
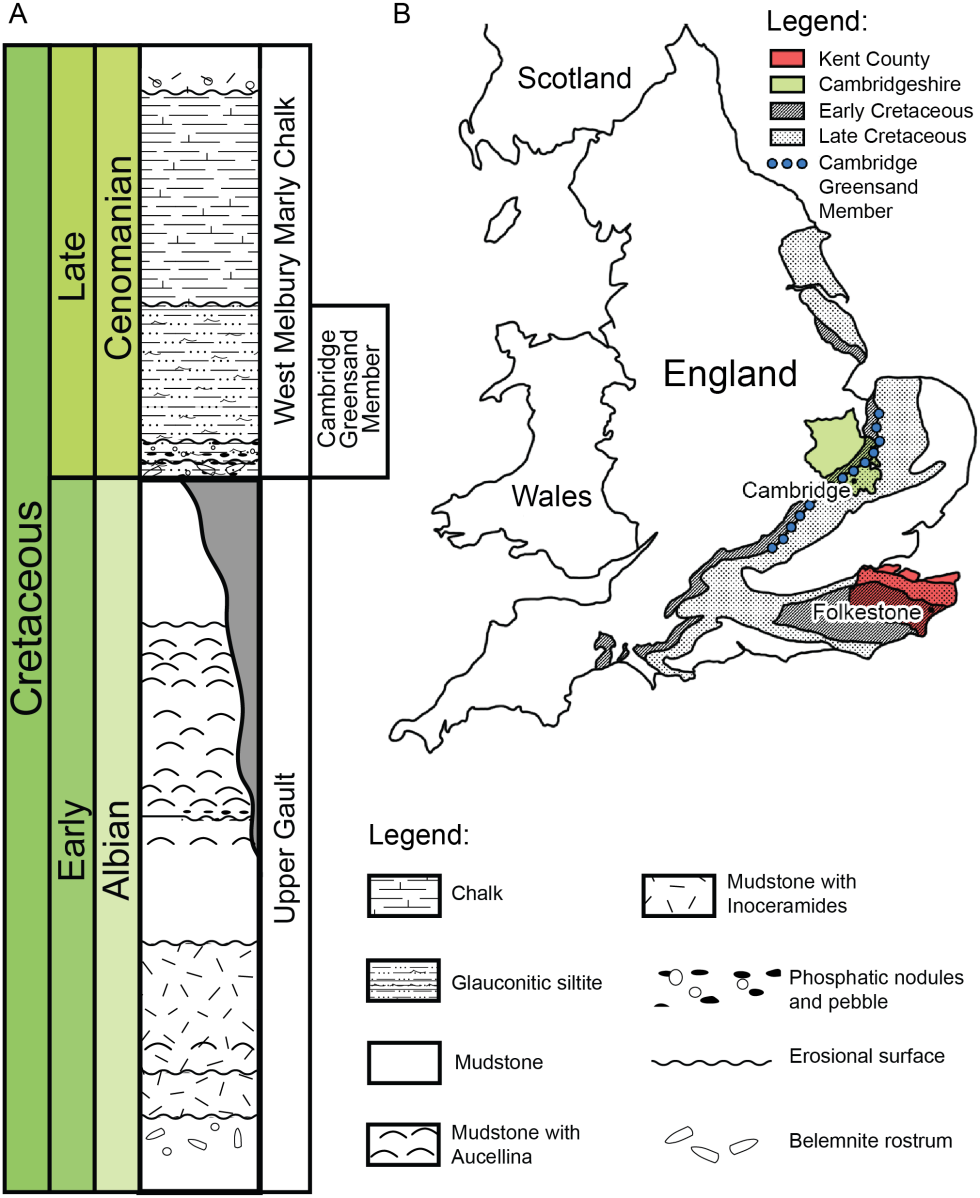
(A) Schematic log modified from Unwin (2001) containing the lithology and the stratigraphic position of the Cambridge Greensand Member. (B) Schematic map positioning the Cretaceous deposits of England. The Cambridge Greensand Member is situated at their junction (modified from Benton & Spencer (1995)).

The holotype of *Rhinochelys amaberti* Moret (1935) (UJF-ID.11167) was found in the Vallon de la Fauge, near Villard-de-Lans in the Isère department (France) (Fig. 1) (Moret, 1935). The Vallon de la Fauge area belongs to a larger geological and geographical unit called the Vercors. In the northeastern Vercors, where the Vallon de la Fauge is located, the thick carbonate successions of the Barremian-Early Aptian are surimposed by bioclastic limestone from the Late Aptian (about 15 m thick), followed by several meters of Albian greenish sandstones and glauconitic marls in decimetric beds (Fig. 1). These Albian deposits are known locally as ‘Béton phosphaté’ because of their abundant phosphatized ammonites and bioclasts (Arnaud, 1988). The sandy and marly deposits overlying the carbonates belong to the Marnes Bleues Formation, which extends from the Aptian to the Cenomanian (Bréhéret, 1997) (Fig. 1). The outcrop in the Vallon de la Fauge solely consists of these glauconitic and sandy marls and has been dated from the *Stoliczkaia dispar* Zone (Late Albian, late Early Cretaceous; Moret, 1935). Thus, *Rhinochelys amaberti* is roughly contemporaneous with the specimens from the Cambridge Greensand Member (Fig. 2) (West Melbury Marly Chalk Formation, UK; Hopson, 2005; Martill & Unwin, 2012) and bears the same coloration as the specimens (see Fig. S1–S8) from Cambridge Greensand Member (Fischer et al., 2014).

### Geography and geology of the Cambridge Greensand Member specimens

We describe a series of previously unreported testudine fossils originating from the Cambridge Greensand Member in the Cambridgeshire (Fig. 2) (UK) (see Figs. S1–S8). The Cambridge Greensand Member (Fig. 2) is a Cenomanian remanié deposit, corresponding to the *Mantelliceras mantelli* Zone and the *Neostlingoceras carcitanense* Subzone (Hopson, 2005). The Cambridge Greensand Member must have formed in a shallow epeiric sea environment (Unwin, 2001); its fauna comprises vertebrate and invertebrate remains (e.g.,: pterosaurs, ichthyosaurs, turtles, plesiosaurs, etc. Benton & Spencer, 1995) either dating from Cenomanian or Late Albian (Unwin, 2001). The latter have a dark brown color due to their phosphatised nature (Benton & Spencer, 1995) and originate from the underlaying Gault Formation (Fig. 2) (Benton & Spencer, 1995; Hopson, 2005; Martill & Unwin, 2012; Fischer et al., 2014).

### Phylogenetic analyses

There is a considerable disagreement on the intra-generic diversity of *Rhinochelys*. As much as 16 distinct species have been proposed in the past (Seeley, 1869; Lydekker, 1889a; Moret, 1935; Collins, 1970), but more recent studies have considered one or two valid species of *Rhinochelys*: *R. pulchriceps* and *R. nammourensis* (Tong et al., 2006; Hooks, 1998; Hirayama, 1994), notably due to a lack of characters to differentiate them in existing datasets (Hooks, 1998). Nevertheless, there is a notable intrageneric disparity of skull morphologies *Rhinochelys* (Collins, 1970), which we confirmed by our study of a series of unreported skulls from the Cambridge Greensand Member (see Supplementary Information). To test this, we established a series of morphotypes (*Rhinochelys* morphotype ‘*cantabrigiensis*’, *Rhinochelys* morphotype ‘*elegans*’, and ‘GS63 GS67’), based on the data from Owen (1851), Lydekker (1889b), Lydekker (1889a) and Collins (1970) and our own interpretations (see Table 1), and we assessed their phylogenetic position. The rationale for each morphotype referal can be found in the Supplementary Information.

**Table 1:** Taxa assignation of Cambridge Greensand Member from the RBINS Collections.

Specimen	Taxonomic assignation	References
IRSNB GS63, IRSNB GS67	*Rhinochelys* indet.	Owen (1851), Lydekker (1889a), Moret (1935), Collins (1970)
IRSNB GS64, IRSNB GS65	*Rhinochelys* morphotype ‘*elegans*’	Lydekker (1889a), Moret (1935), Collins (1970)
IRSNB GS68, IRSNB GS70	*Rhinochelys pulchriceps*	Owen (1851), Lydekker (1889a), Moret (1935), Collins (1970)

We modified the dataset of Cadena & Parham (2015). This matrix was chosen as the most recent dataset available for Testudines with a wide character sampling. We added four new taxa to this dataset: (i) *Rhinochelys amaberti*, using the holotype skull (UJF-ID.11167) and the associated mandible; (ii) *Rhinochelys* morphotype ‘*elegans*’ Lydekker (1889a), using the specimens IRSNB GS64 and IRSNB GS65 (see Figs. S1 and S2; see Table 1), as well as the description and drawings of Lydekker (1889a) and Collins (1970); (iii) *Rhinochelys* morphotype ‘*cantabrigiensis*’ Lydekker (1889a) based on the description and pictures of the holotype from Lydekker (1889a) and Collins (1970); and (iv) IRSNB GS63 and IRSNB GS67 (see Figs. S7 and S8; see Table 1) as representatives of a potentially new taxon from the Cambridge Greensand. We also revised the scores of *Rhinochelys pulchriceps* (Owen, 1851) using IRSNB GS68 (see Figs. S3–S5; see Table 1) plus the descriptions and pictures of Owen (1851), Lydekker (1889a) and Collins (1970). Finally, we have corrected a series of erroneous scores for *Syllomus aegyptiacus* (Lydekker, 1889b) (character 10 is scored 1 instead of 0, and character 40 is scored 1 instead of ?) (Weems, 1980), *Euclastes platyops* Cope (1867) (character 1 is scored 1 instead of ?, and character 10 is scored 0 instead of ?) (Hay, 1905), *Euclastes acutirostris* Jalil et al. (2009) (character 10 is scored 0 instead of 1) (Jalil et al., 2009), *Pacifichelys* (character 10 is scored 0 instead of 1), *Natator depressus* (Garman, 1880) (character 10 is scored 0 instead of 1) (Brinkman et al., 2009).

The original character set of Cadena & Parham (2015) could not resolve the relationships of early sea turtles (see Fig. S9). Indeed, in this dataset, ‘middle’ Cretaceous (Aptian-Turonian) chelonioid all have identical or nearly identical character scoring, necessarily resulting in a large polytomy (Cadena & Parham, 2015). This effect was further increased with the new taxa added above. Moreover, the presence of a large number of unrelated continental (including freshwater) taxa in the original matrix likely produced far-field effects that split Pan-Chelonioidea among Cryptodira (‘full matrix’; see Fig. S9) (Myers et al., 2017). To cope with these issues, we removed all taxa that are likely non-marine, and chose the Late Jurassic *Solnhofia parsonsi* Gaffney (1975) as the outgroup. *Solnhofia* is a pan-cryptodiran that appears clearly outside the Pan-Chelonioidea clade, making a sensible outgroup for the present analysis, as the relationships of more derived pan-cryptodirans has not reached a consensus (e.g., Zhou & Rabi (2015); Cadena & Parham (2015)). In Cadena & Parham (2015), *Solnhofia* is the closest taxon to the marine turtle node without being part of it. As mentionned above, new characters are needed to recover the relationships of chelonioids. We have thus added (and illustrated) 10 new characters and altered the character #10 of Cadena & Parham (2015), using our observations of Albian cheloninoids as well as thorough analysis of the literature (see Figs. S10–S19). These characters underwent a critical evaluation based on the recommendations of Simoes et al. (2016). We used Mesquite v.3.10 to manage the nexus file. The resulting dataset (‘cheloninoid matrix’) contains 48 taxa and 266 characters.

We also assess the phylogenetic relationships of chelonioids using a different but widely used dataset: the matrix from Bardet et al. (2013), which stems from Kear & Lee (2006). Five new taxa were added to this dataset (called ‘Bardet matrix full’): (i) *Rhinochelys nammourensis* using descriptions and pictures from Tong et al. (2006); (ii) *Rhinochelys amaberti*; (iii) *Rhinochelys* morphotype ‘*elegans*’; (iv) *Rhinochelys* morphotype ‘*cantabrigiensis*’ and (v) ‘IRSNB GS63 IRSNB GS67’ (using the same data sources as in the chelonioid matrix discussed above). No new characters were added in this matrix in order to maintain two widely distinct datasets.

All phylogenetic datasets were analyzed in maximum parsimony using the New Technology Search (parsimony ratchet) of TNT v.1.5 (Goloboff & Catalano, 2016) (drift and ratchet activated; 200 ratchet iterations) to identify a series of ‘best candidate’ trees that were then subjected to tree branch reconnections. Bremer Decay indices were computed using TNT v.1.5. Because the strict consensuses of both datasets were largely unresolved due to the inclusion of poorly known OTUs, we used the a posterior method ‘Iterative Positional Congruence Reduced’ (IterPCR) established by Pol & Escapa (2009), and recently implemented within TNT v.1.5 (Goloboff & Catalano, 2016), to identify wildcard taxa. For the ‘Bardet matrix’, the removal of *Rhinochelys* morphotype ‘*cantabrigiensis*’ was sufficient to recover a well-resolved consensus (called ‘Bardet matrix’ throughout). All strict consensus cladograms were computed and time calibrated (ages obtained from paleobiology database) in R using the strap package (Bell & Lloyd, 2015). Branch lengths were reconstructed using both the ‘basic’ and ‘equal’ methods. The stratigraphic congruence of the most parsimonious trees was evaluated using the ape (Paradis, Claude & Strimmer, 2004), geoscale (Bell & Lloyd, 2015), and paleotree (Bapst, 2012), and strap (Bell & Lloyd, 2015) packages.

By all means, our phylogenetic analyses should be regarded as preliminary, as it appears clear from our results that much more data are needed to recover robust relationships among chelonioids. To further test the robustness of some of our results, we used Templeton’s nonparametric test implemented in PAUP* (Templeton, 1983). This method establishes the statistical difference of length between topologies. The trees were constrained using the force function in TNT.

### Systematic palaeontology

**Table:** 

Testudines Linnaeus, 1758
Cryptodira Cope, 1868
Chelonioidea Oppel, 1811
Protostegidae Cope, 1873
*Rhinochelys* Seeley (1869)
*Rhinochelys amaberti* Moret (1935)

#### Holotype, stratum typicum, and locus typicus

The holotype of *Rhinochelys amaberti* is UJF-ID.11167, a well preserved partial cranium from the late Albian (*Stoliczkaia dispar* Zone) of the Vallon de la Fauge (Isère, France). The holotype also includes a mandible that was found at the exact same locality and horizon as the cranium, although several years later. The size, shape, and diagenesis of this mandible are compatible with the holotype cranium.

#### Emended diagnosis

*Rhinochelys amaberti* possesses the following autapomorphies (among Protostegidae): triangular- shaped nasal bone; the antorbital bulge formed by the maxilla (dorsal to the maxillary sulcus) and the prefrontal is strongly prominent to the point of concealing the labial edges of the maxilla in dorsal view, and is even visible in ventral view (this bulge is also present but smaller in *Rhinochelys pulchriceps*, *Rhinochelys nammourensis, Rhinochelys* morphotype ‘*elegans*’ and *Rhinochelys* morphotype ‘*cantabrigiensis*’); mediolaterally narrow, nearly absent nasal-frontal contact; large dorsal extension of the frontal; oval nasal fossa (with horizontal long axis); nasal fossa forms a deep indentation in the maxilla in lateral view; the skull is dorsoventrally compressed, barely protruding dorsally and ventrally from the orbit; the parietal-frontal surface is parallel to the ventral surface of the maxilla.

### Description of the holotype of *R. amaberti*

**Figure 3:**
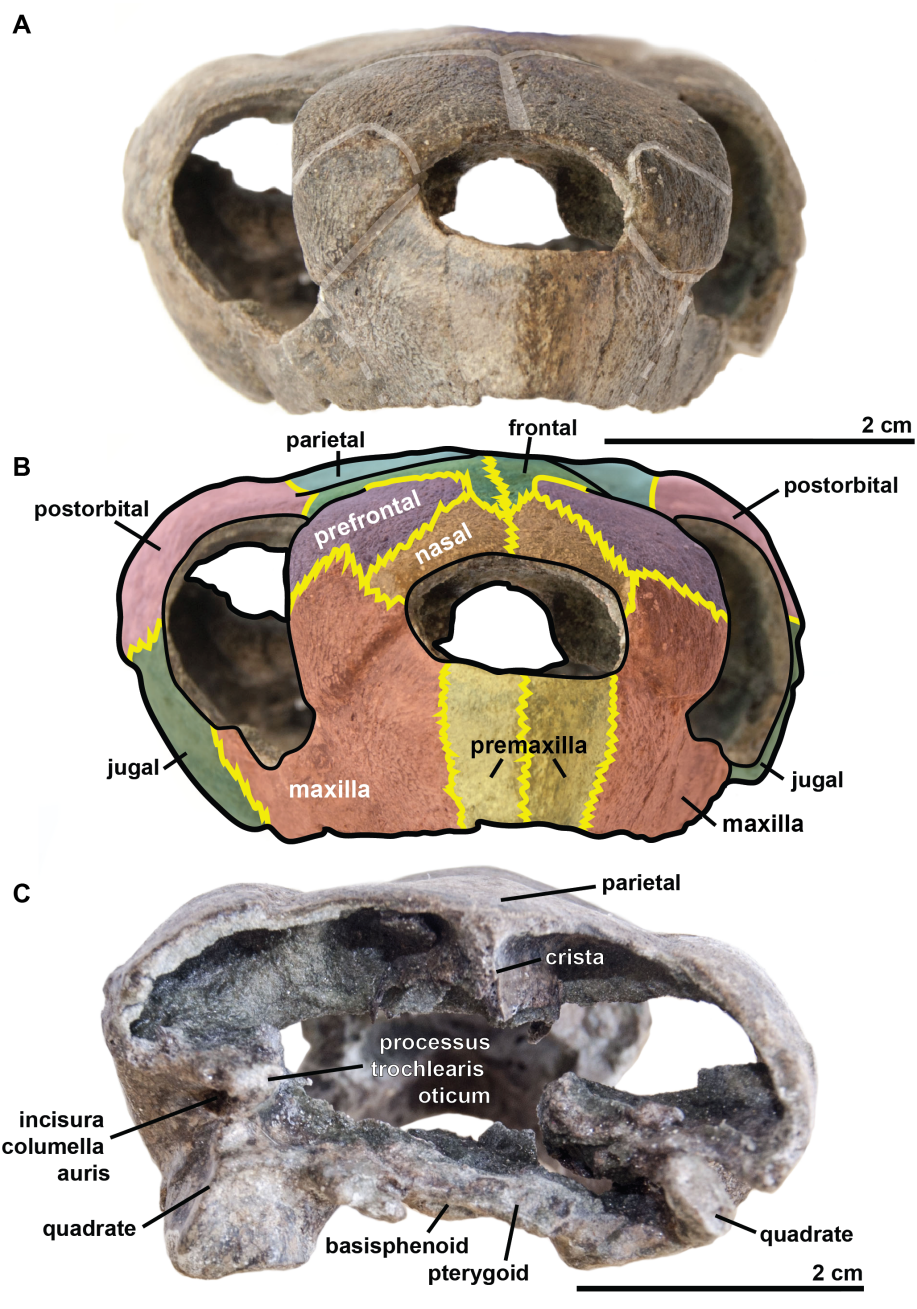
Skull of the holotype (UJF-ID.11167) of *Rhinochelys amaberti* in (A, B) anterior and (C) posterior views. In (A) scute sulci are colored in transparent white. In (B) bone sutures are colored in yellow.

**Figure 4:**
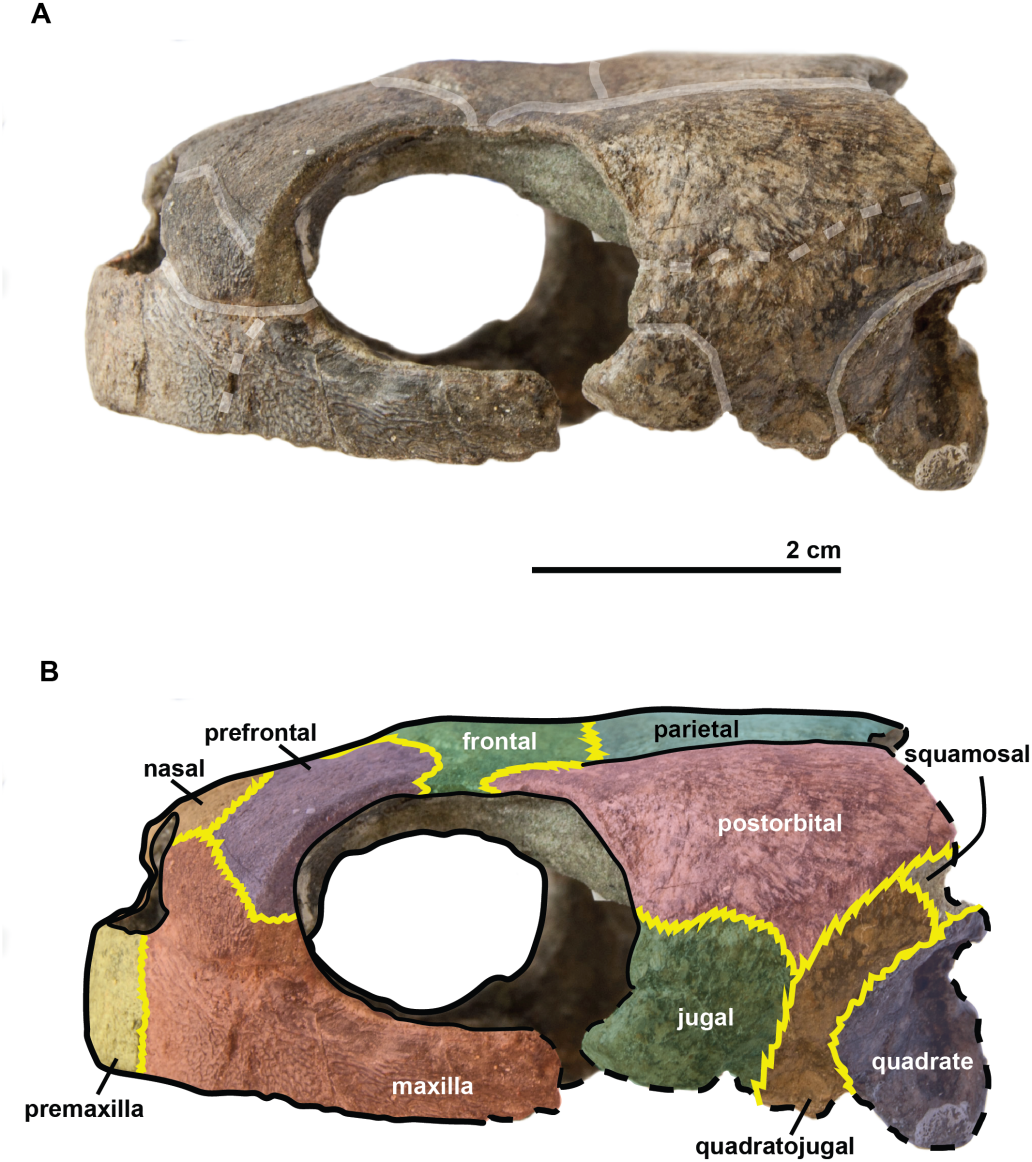
Skull of the holotype (UJF-ID.11167) of *Rhinochelys amaberti* in lateral view showing the left side. In (A) scute sulci are colored in transparent white. In (B) bone sutures are colored in yellow, black dashed lines highlight the broken bones and white dashed lines show the probable position of a scute sulcus.

**Figure 5:**
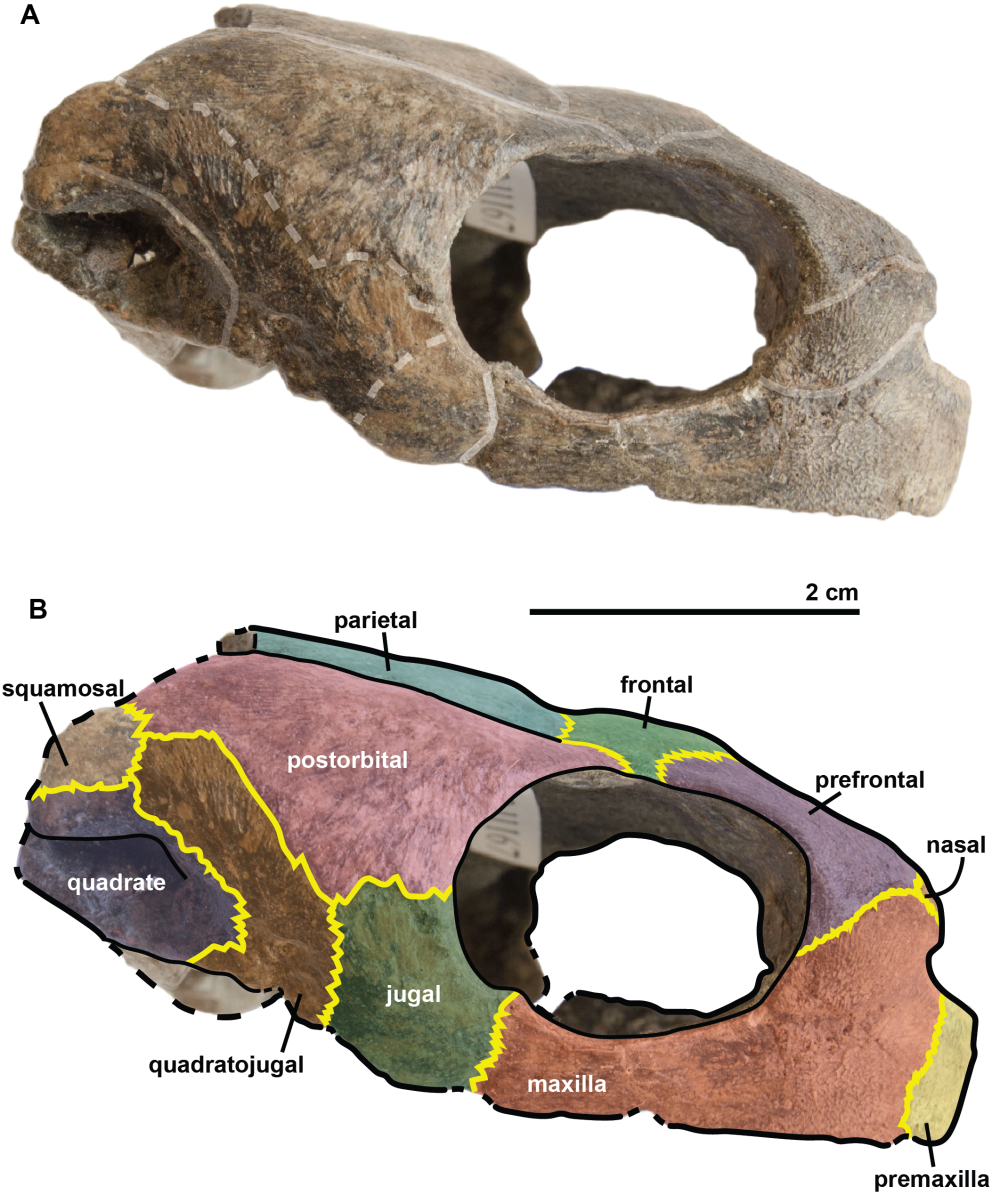
Skull of the holotype (UJF-ID.11167) of *Rhinochelys amaberti* in lateral view showing the right side. In (A) scute sulci are colored in transparent white. In (B) bone sutures are colored in yellow, black dashed lines highlight the broken bones and white dashed lines show the probable position of a scute sulcus.

**Figure 6:**
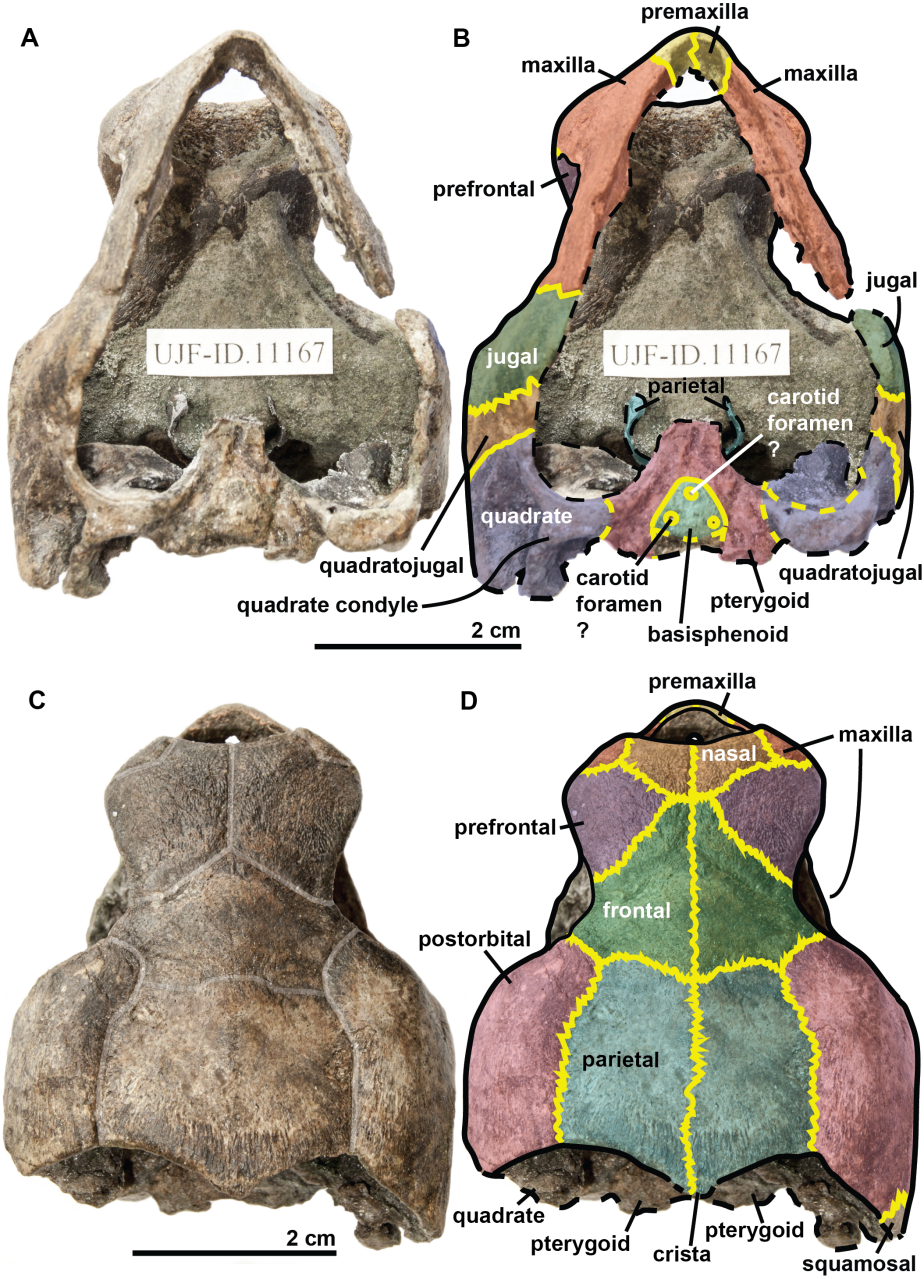
Skull of the holotype (UJF-ID.11167) of *Rhinochelys amaberti* in (A) ventral and (B) dorsal views. In (B) and (D) bone sutures are colored in yellow, black dashed lines highlight the broken bones, and yellow dashed lines show the position of unpreserved suture lines. In (C) scute sulci are colored in transparent white.

The **cranium** is 5.3 cm long and 2.4 cm high. It is dorsoventrally compressed (Fig. 3) and is wider than it is long. This general shape of the cranium thus differs from those of *R. pulchriceps* (Owen, 1851; Lydekker, 1889a; Moret, 1935; Collins, 1970); *Rhinochelys* morphotype ‘*elegans’* (Lydekker, 1889a; Moret, 1935; Collins, 1970) and *R. nammourensis* (Tong et al., 2006), but appears similar to that of *Rhinochelys* morphotype ‘*cantabrigiensis’* (Lydekker, 1889a; Moret, 1935; Collins, 1970) in having a skull that is wider than long. The anterior surface of the skull is vertically oriented in lateral view (Fig. 4), resulting in an anteroposteriorly short skull lacking a rostrum or a beak unlike in *Archelon* (Hay, 1908), *Ocepechelon* (Bardet et al., 2013) and *Protostega* (Hay, 1908), but is similar to *Ashleychelys* (Weems, 2014). Posteriorly to the level of the naris, the skull rapidly reaches its maximum height, at the level of the first half of the anterior margin of the orbit (Figs. 4 and 5). Such a skull shape is also seen in *R. pulchriceps* (Owen, 1851; Lydekker, 1889a; Moret, 1935; Collins, 1970). In dorsal view (Fig. 6), the skull expands laterally just posterior to the orbits. This lateral expansion of the skull extends up to the postorbital-jugal-quadratojugal suture, as in *R. pulchriceps* (Owen, 1851; Lydekker, 1889a; Moret, 1935; Collins, 1970) and unlike in *Rhinochelys* morphotype ‘*elegans’* or *Rhinochelys* morphotype ‘*cantabrigiensis’* (Lydekker, 1889a; Moret, 1935; Collins, 1970).

The **maxilla** possesses a shallow sinusoidal sulcus (Figs. 4 and 5) which connects the nasal cavity and the orbit. The maxilla forms an important bulge, dorsally to this sulcus. This bulge is here larger than in any other species currently referred to as *Rhinochelys* (Lydekker, 1889a; Moret, 1935; Collins, 1970). This feature does not seem to be age-related in protostegids (Collins, 1970; Tong et al., 2006). As a consequence, the maxillary bone is hardly visible in dorsal view whereas in ventral view it is predominant and conceals a great portion of the prefrontal (Fig. 6). In all other species currently referred to as *Rhinochelys* (except *R. pulchriceps*, see Figs. S3 and S6), the bulge is shallow and cannot be seen in ventral view but only in anterior and dorsal views (Lydekker, 1889a; Collins, 1970) (also see Figs. S1, S2, S7 and S8). This bulge has, however, been regarded as a common trait among *Rhinochelys* (Lydekker, 1889a; Moret, 1935; Collins, 1970; Tong et al., 2006). The maxilla also contributes to the orbital rim (Fig. 5) up to the three-fourths of its ventral margin (infraorbital margin), and up to the mid-height of its anterior margin (as in the other species currently referred to as *Rhinochelys*) (Lydekker, 1889a; Moret, 1935; Collins, 1970). The maxilla contacts the nasal at the dorsal and lateral extremities of the nasal fossa. The maxilla also contacts the premaxilla and the prefrontal.

The **parieto-frontal** dome is parallel to the ventral surface of the maxilla, but is not completely flat (Figs. 4 and 5). Such an extended flattened parieto-frontal dome is also found in *R. pulchriceps* (Owen, 1851; Lydekker, 1889a; Moret, 1935; Collins, 1970). There are several cranial sulci running across the frontal and parietal (Fig. 6). These sulci contain the parietal-postorbital and frontal-postorbital sutures, and form a ‘Y’ structure on the frontals which then continues on the nasals (the stem of the ‘Y’ coincides with the median suture of theses bones). The **parietal** is short and wide, smaller than that of *R. pulchriceps* (Owen, 1851; Collins, 1970) and *R. nammourensis* (Tong et al., 2006).

In profile, the **‘snout’** (formed by the premaxillae and extremity of maxillae; Figs. 4 and 5) of *R. amaberti* is shorter and narrower than that of *R. pulchriceps*. Indeed, the ventral view reveals that the snout of *R. amaberti* forms a ‘V’ shape (Fig. 6) rather than the ‘U’ shape seen in *R. pulchriceps* (Owen, 1851; Lydekker, 1889a; Collins, 1970).

The **nasal cavity** is oval in shape, with its long axis oriented horizontally (Fig. 3). In profile, the ventral edge of the nasal fossa, which is formed by the maxilla, forms a right angle (Figs. 4 and 5). This region is rounded rather than angular in the other species currently attributed to *Rhinochelys* (i.e., *‘R. cantabrigiensis’, ‘R. jessoni’* (Lydekker, 1889a; Moret, 1935; Collins, 1970)) or clearly forms an obtuse angle (i.e., *R. pulchriceps*, and *Rhinochelys* morphotype ‘*elegans’* (Owen, 1851; Lydekker, 1889a; Moret, 1935; Collins, 1970)) (see also Figs. S1–S3, S6–S8).

In comparison to other chelonioids, the **orbit** is large compared to the size of the skull: in profile, its height almost makes up for the total height of the skull (Figs. 4 and 5), which is a common feature of the genus *Rhinochelys* (see also Fig. S4) (Owen, 1851; Lydekker, 1889a; Collins, 1970). The orbit of *R. amaberti* is oval in shape, as in *R. pulchriceps* (Owen, 1851; Lydekker, 1889a; Collins, 1970), and unlike the rounded shape seen in *Rhinochelys* morphotype ‘*elegans*’, *Rhinochelys* morphotype ‘*cantabrigiensis*’, (Lydekker, 1889a; Moret, 1935; Collins, 1970). The dorsal opening of the orbit seems greater than that of other *Rhinochelys* species (Owen, 1851; Lydekker, 1889a; Moret, 1935; Collins, 1970) with the exception of *R. nammourensis* (Tong et al., 2006). The orbit is still facing laterally like in the other *Rhinochelys* (Owen, 1851; Lydekker, 1889a; Moret, 1935; Collins, 1970; Tong et al., 2006), and unlike in *Euclastes* (Hay, 1908; Lynch & Parham, 2003; Hirayama & Tong, 2003; Jalil et al., 2009), *Ocepechelon* (Bardet et al., 2013), *Alienochelys* (De Lapparent de Broin et al., 2014), and *Allopleuron* (Ubaghs, 1875). The orbital rim is formed by the maxilla (the most important contribution), jugal, postorbital, frontal (the smallest contribution) and prefontal.

The **frontal** is large and makes up an important portion of the upper orbital rim (Fig. 6). The anterior expansion meeting the nasal bone is extremely small, preventing the prefrontals from meeting medially. However it possesses an important dorsal expansion. The sulcus forming a ‘V’ shape medially (see above) is narrower than the one of *Rhinochelys* morphotype ‘*cantabrigiensis’* and *Rhinochelys* morphotype ‘*elegans’* (Lydekker, 1889a; Moret, 1935; Collins, 1970), but similar to that of *R. pulchriceps* (Owen, 1851; Lydekker, 1889a; Moret, 1935; Collins, 1970). The frontal contacts the nasal bone, prefontal, postorbital and parietal. The surface defined by the nasal and frontal (Figs. 4 and 5) is inclined dorsally with respect to the ventral surface of the maxilla by an angle of 25°. As in *R. pulchriceps*, the participation of the frontal to the supraorbital rim is small (Owen, 1851; Lydekker, 1889a; Collins, 1970), this frontal participation is clearly larger in *Rhinochelys* morphotype ‘*elegans’* and *Rhinochelys* morphotype ‘*cantabrigiensis’* (Lydekker, 1889a; Moret, 1935; Collins, 1970).

A **nasal** bone is present (Figs. 3 and 6), which is a feature found in all protostegids (Zangerl, 1953). It is wider than it is long (6 mm × 1.6 mm), and shows a 50° angle with respect to the ventral surface of the maxilla (Figs. 4 and 5). The nasal meets the maxilla dorsally to the upper margin of nasal fossa (Figs. 4–6); their suture extends up to the dorsal border of the nasal fossa (Figs. 4 and 5). Nasal and frontal also share a suture which prevents the prefrontals from meeting medially. This suture is extremely reduced, compared to other species currently assigned to the genus *Rhinochelys* (Owen, 1851; Lydekker, 1889a; Moret, 1935; Collins, 1970; Tong et al., 2006). The nasal bone meets the frontal, prefrontal and the maxillary bone.

The **prefontal** (Fig. 6) is large and possesses an important dorsal expansion (greater than in other species currently attributed to *Rhinochelys* (Owen, 1851; Lydekker, 1889a; Moret, 1935; Collins, 1970; Tong et al., 2006)) which is however smaller than the dorsal expansion of the frontal (like in other species currently assigned to *Rhinochelys* (Owen, 1851; Lydekker, 1889a; Moret, 1935; Collins, 1970; Tong et al., 2006)). The frontals almost meet medially but are separated by the thin nasal-frontal suture (Fig. 6). Thus the frontal only shares a suture with the maxilla, nasal and frontal. The prefrontal contributes greatly to the supraorbital margin, especially in comparison to the frontal. This prefrontal contribution is more important than in other *Rhinochelys* species (Owen, 1851; Lydekker, 1889a; Moret, 1935; Collins, 1970; Tong et al., 2006). The prefrontal also accounts for the anterior bulge of the skull, but to a lesser degree than the maxilla.

The **postorbital** reaches ventrally to the mid-height of the orbits where it meets the jugal (Figs. 4 and 5). Posteriorly, it shares a suture with the quadratojugal and the squamosal, but does not contact the quadrate. Dorsally, the postorbital also starts at the mid-length of the orbits where it meets the frontal and the parietal. The dorsal expansion of the postorbital is well developed as the dorsal width of the skull extends as far as the postorbital-jugal-quadratojugal suture (Fig. 6). The covering of the temporal region by the postorbital, squamosal and parietal is a protostegid feature (Zangerl, 1953).

The **ventral border of both maxilla and premaxilla** is entirely flat; there is no anterior notch or lifting forming a beak (Figs. 4 and 5). This differs from the other species of *Rhinochelys* found in the UK (Owen, 1851; Lydekker, 1889a; Moret, 1935; Collins, 1970). *‘Rhinochelys elegans’* possesses a rather flat surface since the ventral margin of its premaxilla slightly rises dorsally (Lydekker, 1889a; Moret, 1935; Collins, 1970).

The **premaxillary profile** is straight (Figs. 4 and 5) like *Rhinochelys* morphotype ‘*elegans’* (Lydekker, 1889a; Collins, 1970) (while it is convex in the other species of *Rhinochelys* (Owen, 1851; Lydekker, 1889a; Moret, 1935; Collins, 1970)). The premaxilla is rather small, and its height is slightly greater (7 mm) than its width (5 mm).

The **quadrate** is circular in shape (Figs. 4 and 5) but is dorso-ventrally crushed and not complete (posteriorly broken). The quadrate entirely comprises the cavum tympani, like in the other species currently assigned to *Rhinochelys* (Owen, 1851; Lydekker, 1889a; Moret, 1935; Collins, 1970; Tong et al., 2006). It shares a suture with the quadratojugal, squamosal and pterygoid. The quadrate also bear the incisura columella auris and the incomplete processus trochlearis oticum. These can be seen in posterior view of the skull (Fig. 6). The ventral surface of the quadrate condyle is almost flat, and is twice as wide than long.

The **quadratojugal** is semi-circular in shape and partially wraps around the quadrate (Figs. 4 and 5): at the dorsal-most point of the quadrate, the quadratojugal contacts the squamosal which then wraps around the other half of the quadrate. These morphologies are also found in the other species of *Rhinochelys* (Owen, 1851; Lydekker, 1889a; Moret, 1935; Collins, 1970; Tong et al., 2006). The quadratojugal contacts the jugal, postorbital, squamosal, and the quadrate. The **squamosal** is not complete and meets the quadrate, quadratojugal and the postorbital (Figs. 4 and 5).

The **jugal** is crescent-shaped, as is the quadratojugal, but its concavity is anteriorly oriented (Figs. 4 and 5). It meets the maxilla at the last posterior fourth of the orbit, the postorbital at the posterior mid-height of the orbits, and the quadratojugal posteriorly. This jugal morphology is similar to that of *R. pulchriceps* (Owen, 1851; Lydekker, 1889a; Moret, 1935; Collins, 1970; Tong et al., 2006). However, the jugal is of similar size and expansion in all of the other species currently referred to as *Rhinochelys*, and also possesses the same sutures (which differ in size and position).

The **basisphenoid** and **pterygoid** are damaged and only visible in ventral view (Fig. 6). The basisphenoid is triangular in shape, shows at least three foramina, and separates the pterygoids posteriorly. Several arterial patterns have been highlighted among living and extinct turtles, which are based on one of the two broad forms: (A) either the bifurcation between the palatal and cerebral branches of the carotid artery is floored by bones, (B) or this split occurs outside the skull (Sterli & Fuente, 2010; Sterli et al., 2010). The posterior extension of the pterygoid in ventral view also influences the position of the carotid foramina (Myers et al., 2017). These foramina might not be the cerebral and palatal branches of the carotid artery because a single foramen should not be paired in derived turtles (D Brinkman, pers. com., 2018). Therefore the identity of the remaining foramina remains ambiguous at the moment. The pterygoids meet medially, but their anterior portion is not preserved and thus we cannot observe any foramina to compare with the basisphenoid. The pterygoid contacts the quadrate laterally.

**Figure 7:**
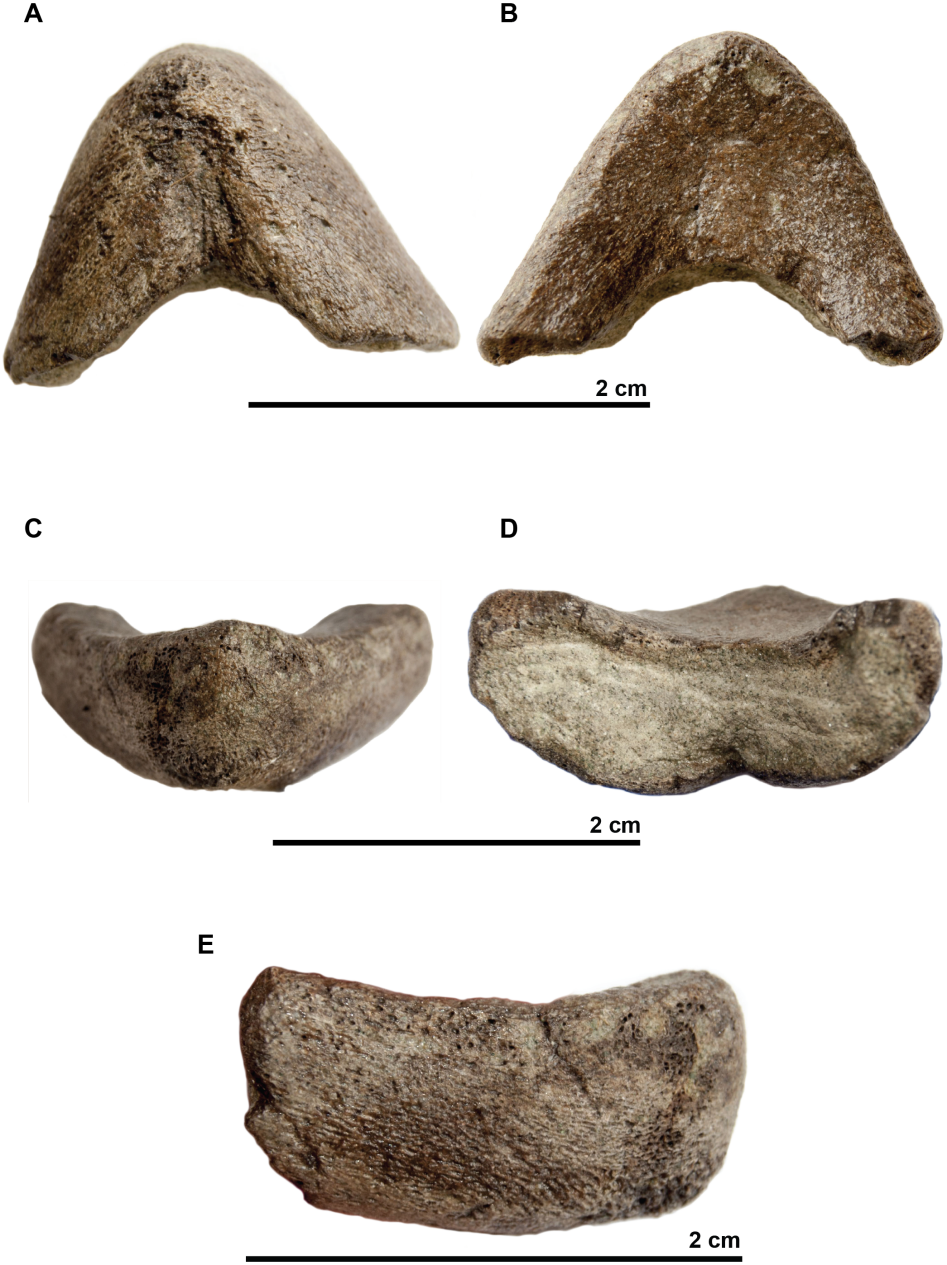
Symphyseal region of dentaries assigned to the skull of the holotype (UJF-ID.11167) of *Rhinochelys amaberti* in (A) ventral view, in (B) dorsal view, in (C) anterior view, in (D) posterior view, and in right (E) lateral view.

The **associated mandible** (Fig. 7) is not complete and only presents partial dentaries. In dorsal view, a thin crest is visible on the anterior part of the symphysis with two small notches on each side. In profile, the dentary reaches its maximal thickness rapidly; it does not thicken posteriorly. The dentary forms a subtle beak: its anterior surface faces mostly anteriorly. In ventral view the symphysis presents a sulcus. If this mandible belongs to the holotype of *Rhinochelys amaberti*, then the symphysis length would be less than one-third of the total jaw length as estimated from the length of the skull.

## Results

### Phylogenetic analyses

**Figure 8:**
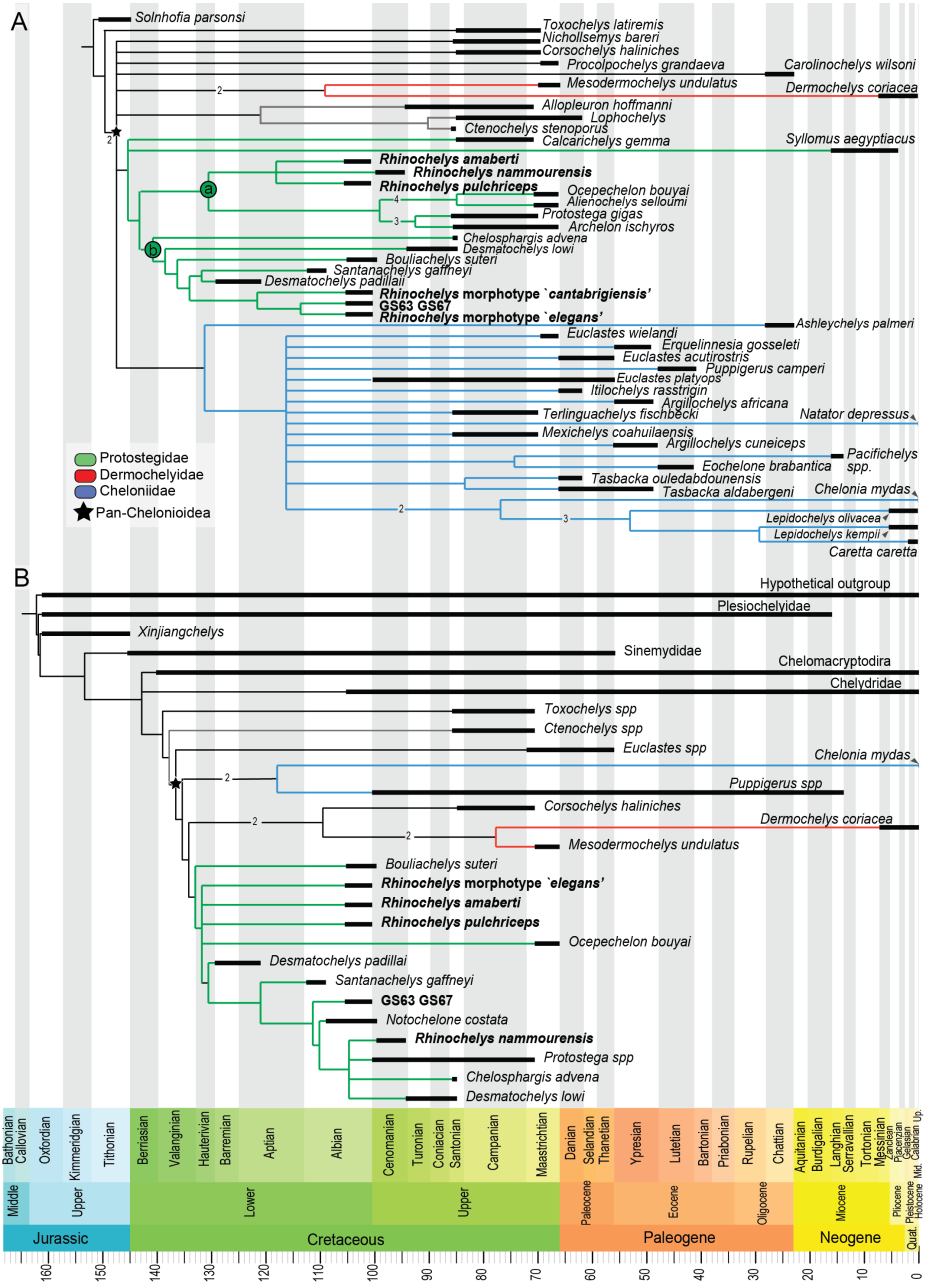
Results from preliminary phylogenetic analyses. (A) Strict consensus cladogram in equal-weights (maximum parsimony) of 42 most parsimonious trees (42 trees, 399 steps long), from a matrix of 266 characters and 48 taxa (‘chelonioid matrix’). Bremer supports values above 1 are indicated near their corresponding node. (B) Strict consensus cladogram in equal-weights (maximum parsimony) of 27 most parsimonious trees (27 trees, 245 steps long), from a matrix of 104 characters and 27 taxa (‘Bardet matrix’). Bremer supports values above 1 are indicated on the branch leading to their corresponding node.

Our analysis of the chelonioid dataset yielded in 42 most parsimonious trees, each with a length of 399 steps. The Consistency Index is 0.421 and the Retention Index is 0.631, giving a Rescaled Consistency Index of 0.266. The strict consensus of the most parsimonious trees (Fig. 8) recovers the three traditional chelonioid families (Protostegidae in green, Cheloniidae in blue, Dermochelyidae in red) but with slightly different compositions than in previous attempts (e.g., Hirayama, 1998; Kear & Lee, 2006). Because of our focus is on Protostegidae and *Rhinochelys* in particular, only some of these differences will be discussed here; others are discussed in the Supplementary Information. The deep nodes of the Pan-Chelonioidea clade are not well resolved, forming a polytomy that includes several other taxa previously regarded as cheloniids (Gaffney & Meylan, 1988; Hirayama, 1994; Hirayama, 1997; Weems, 2014; Brinkman et al., 2015), such as *Corsochelys haliniches*, formerly regarded as dermochelyid (Hirayama, 1994; Hirayama, 1997; Hooks, 1998) or a cheloniid (Bardet, 1995; Kear & Lee, 2006) and *Nichollsemys baieri*, previously regarded as a cheloniid (Cadena & Parham, 2015) or close to *Toxochelys* and cheloniids (Brinkman et al., 2015), which form here part of the Pan-Chelonioidea basal polytomy. This topology resembles that of Brinkman et al. (2006), who also recovered *Nichollsemys* as an early chelonioid. *Ctenochelys* groups with *Allopleuron* and *Lophochelys*, which were formerly regarded as cheloniids (Hirayama, 1994; Hirayama, 1997; Weems, 2014). This clade is also included in the general polytomy. As a consequence, *Toxochelys* is here isolated outside Pan-Chelonioidea.

Protostegidae + Dermochelyidae do not form a clade, unlike in some previous studies of chelonioid relationships (the ‘Dermochelyoidea’ of Gaffney & Meylan (1988); Lehman & Tomlinson (2004); Bardet et al. (2013); De Lapparent de Broin et al. (2014); Cadena & Parham (2015) or the ‘Pandermochelys’ of Kear & Lee (2006)). Basal protostegids include *Calcarichelys* and, unexpectedly, the Neogene taxon *Syllomus aegyptiacus*. This taxon was previously regarded as a cheloniid of uncertain affinities (Lynch & Parham, 2003; Parham & Pyenson, 2010) or a dermochelyid (Cadena & Parham, 2015). The following state changes support the clade uniting *Syllomus* with other protostegids: character 10.0; character 88.0; character 116.3 and character 263.1. The other characters (45, 57, 58, 66, 75, 137, 153, 197, 198, 247, 251) that support the node uniting *Syllomus* with protostegids (i.e., node 73 → 64 from the apomorphy list in Supplementary Information) are unknown for *Syllomus*. We tested the robusteness of the placement of *Syllomus* as a protostegid instead of a cheloniid. In TNT, we constrained tree search to recover a clade uniting *Syllomus* + cheloniids (i.e., *Ashleychelys palmeri, Euclastes wielandi, Euclastes acutirostris, Euclastes platyops, Erquelinnesia gosseleti, Puppigerus camperi, Itilochelys rasstrigin, Argillochelys africana, Argillochelys cuneiceps, Mexichelys coahuilaensis, Terlinguachelys fischbecki, Pacifichelys spp., Eochelone brabantica, Tasbacka ouledabdounensis, Tasbacka aldabergeni, Chelonia mydas, Lepidochelys kempii, Lepidochelys olivacea, Caretta caretta* and *Natator depressus*). This yielded most parsimonious trees that have a length of 413 steps, which is 14 additional steps than in the unconstrained analysis. Still, Templeton’s test (Templeton, 1983) indicates that this solution is not statistically distinguishable (*p* = 0.0668) from the most parsimonious solution (i.e., *Syllomus* as a protostegid).

While *Chelosphargis* and *Calcarichelys* are still recovered as protostegids, they are not closely related, unlike in Zangerl (1960), Gaffney & Meylan (1988), Hirayama (1994), Bardet (1995) and Hirayama (1997). *Calcarichelys* is, however, extremely fragmentary, which might affect its position. Derived protostegids separate into two clades (‘a’ and ‘b’ in Fig. 8). *Santanachelys gaffneyi* (clade ‘b’) is recovered in a more derived position than in Hirayama (1998), Kear & Lee (2006), De Lapparent de Broin et al. (2014) and Cadena & Parham (2015) and is grouped with *Desmatochelys padillai*. This ‘intermediate’ position of *Santanachelys gaffneyi* and *Bouliachelys suteri* in between the two species of *Desmatochelys* suggests that the Barremian taxon *D. padillai* does not belong to *Desmatochelys*, otherwise known from the Turonian-Santonian interval (Cadena & Parham, 2015). This clustering is coherent since *Santanachelys* and the species of *Desmatochelys* also bear several primitive protostegid features (Zangerl, 1960; Elliot, Irby & Hutchison, 1997). *Chelosphargis* remains a protostegid (Zangerl, 1960; Gaffney & Meylan, 1988; Bardet, 1995), and is placed at the most primitive position in clade ‘b’.

Our main result of the analysis of the ‘Chelonioid’ dataset is that the genus *Rhinochelys* is no longer recovered as a basal protostegid and is split in two clades (Fig. 8), *Rhinochelys* morphotype ‘*elegans*’, *Rhinochelys* morphotype ‘*cantabrigiensis*’ and the unnamed taxon represented by the specimens IRSNB GS63 and IRSNB GS67 are placed among clade ‘b’ (close to *D. padillai* and *Santanachelys*), whereas *R. pulchriceps, R. nammourensis* and *R. amaberti* are placed within clade ‘a’. A *Rhinochelys-Desmatochelys* affiliation has been proposed before (Bardet, 1995) and is here recovered for a subset of this genus only (*Rhinochelys* morphotype ‘*elegans*’, *Rhinochelys* morphotype ‘*cantabrigiensis*’, IRSNB GS63-IRSNB GS67). Clade ‘a’ presents two sister lineages (Fig. 8): the clade containing *R. amaberti, R. pulchriceps, R. nammourensis*, and the clade containing both *Archelon-Protostega* and *Ocepechelon-Alienochelys*. This position of the *Ocepechelon-Alienochelys* clade differs from previous studies where they were considered dermochelyoids, and thus sister taxa to dermochelyids and protostegids (alongside *Bouliachelys*) (Bardet et al., 2013; De Lapparent de Broin et al., 2014). The clustering of *Ocepechelon* and *Alienochelys* is possibly attributable to their peculiar morphology. Indeed, these poorly known and aberrant taxa have barely no character available to distinguish them in the dataset we used.

We tested the polyphyly hypothesis of *Rhinochelys* using a Templeton test. In TNT we forced the strict monophyly of *Rhinochelys*; this produced most parsimonious trees with a length of 400 steps, which is just one step longer than the most parsimonious solution. Logically, the Templeton test yielded a high *p*-value (*p* = 0.7389), indicating that the polyphyly and monophyly of *Rhinochelys* are statistically indistinguishable in this dataset.

Our analysis of the ‘Bardet matrix full’ resulted in 99 most parsimonious trees, each with a length of 245 steps (see Fig. S20). We re-run the analyses after deleting the wildcard taxon identified by the IterPCR method (*Rhinochelys* morphotype ‘*cantabrigiensis*’). This resulted in 27 most parsimonious trees, each with a length of 245 steps as well (Fig. 8). The strict consensus cladogram presented in Fig. 8 also recovers a non-monophyletic *Rhinochelys*: *R. amaberti, R. pulchriceps* and *Rhinochelys* morphotype ‘*elegans’* are recovered as closedly related, forming a polytomy close to the base of the clade Protostegidae. *Ocepechelon bouyai* is also included within this polytomy. The morphotype ‘GS63-GS67’ is more closely related to *Santanachelys* than the other *Rhinochelys* species. *R. nammourensis* is recovered as a more derived protostegid, and forms a polytomy with *Protostega* spp., *Chelosphargis* and *Desmatochelys lowi*. Other aspects of the topology differ from the results obtained from the analysis of the chelonioid dataset (Fig. 8). This is possibly due to the narrower sampling of taxa and different character set. Both dataset agree that the genera *Desmatochelys* and *Rhinochelys*, as presently defined, might not be monophyletic.

### Stratigraphic congruence

**Figure 9:**
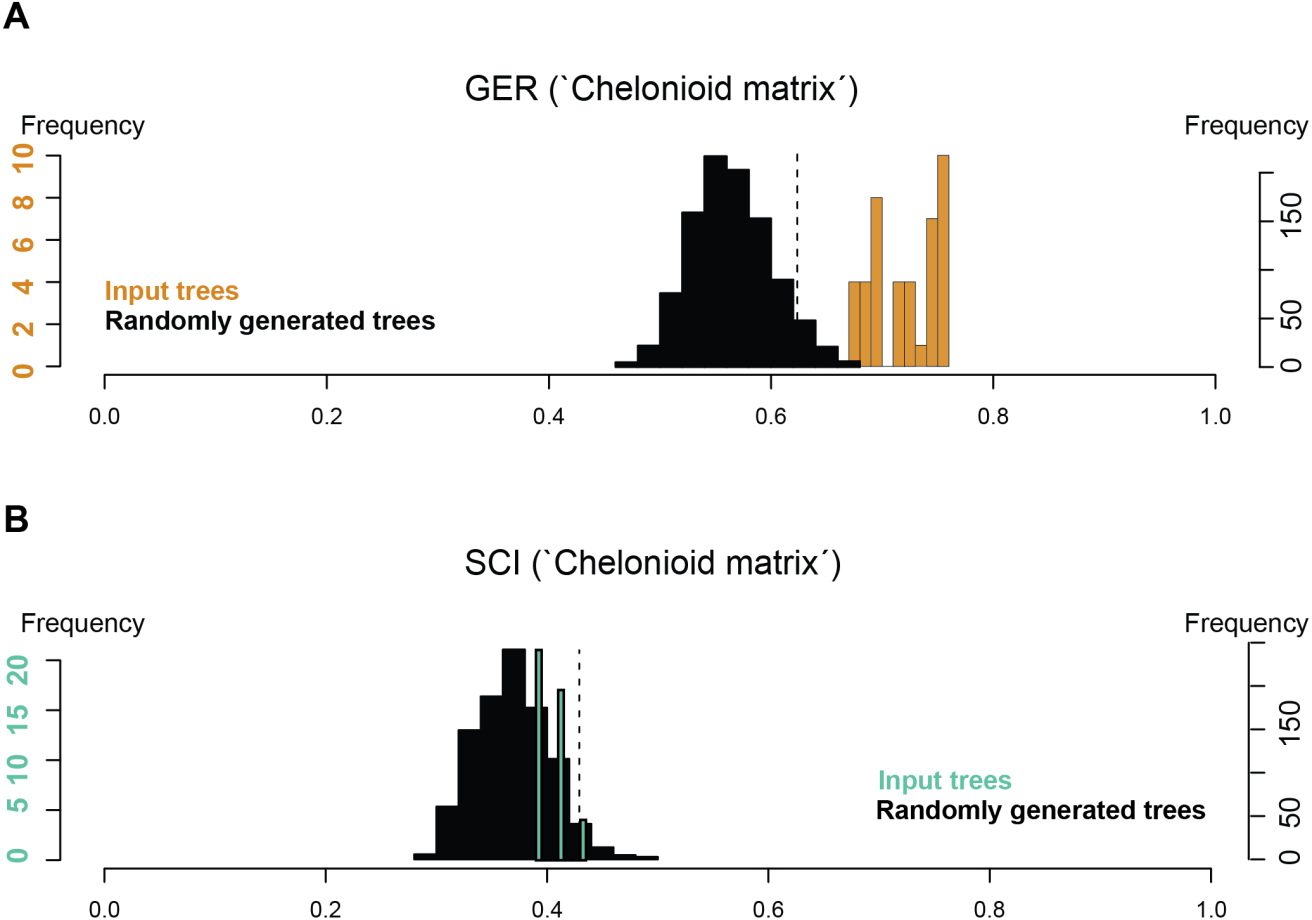
Stratigraphic congruence scores (GER and SCI) of the ‘chelonioid matrix’: (A) GER scores for the ‘chelonioid matrix’; (B) SCI scores for the ‘chelonioid matrix’

Stratigraphic congruence metrics were computed for the ‘chelonioid’ matrix (Fig. 9). For the two metrics considered (the Gap Excess Ratio, GER, and the Stratigraphic Congruence Index, SCI), the scores obtained for the input trees are at most marginally better than that of randomly generated trees (with slightly better results for the GER than the SCI). These indicate weak stratigraphic congruence for the most parsimonious trees, whose topologies force long ghost lineages. This suggests that either the morphological data presently available severely biases the phylogeny of fossil chelonioids and/or that the sampling of fossil chelonioids is poor.

## Discussion

The redescription of *Rhinochelys amaberti* reveals several shared features with *R. pulchriceps*, the type species of *Rhinochelys*. This similarity is confirmed by our phylogenetic analyses (Fig. 8). Indeed, in our most parsimonious trees, *R. pulchriceps* and *R. amaberti* are always closely related, contrary to other taxa or morphotypes referred to as *Rhinochelys*. Because *R. pulchriceps* in the type species of the genus, we advocate that *R. amaberti* likely belongs to *Rhinochelys*, but as a distinct species, given the eight autapomorphic features we described above. The osteological study of *R. amaberti* and comparisons with other *Rhinochelys* specimens suggest the existence of a noticeable disparity of skull architectures within *Rhinochelys*. Two main skull types can be recognized: (i) flattened and thus dorsally facing parieto-frontal dome, cross-shaped frontals, small participation of frontals to orbital rim and important maxillary bulge; (ii) skull with a high profile due to anterodorsal inclination of nasal-frontal surface, T-shaped frontals, moderate participation of frontals to orbital rim and lesser/weak maxillary bulge. These differences ramify into the phylogenetic results, with the most parsimonious solutions being the polyphyly of the genus *Rhinochelys*, as currently conceived. As shown above, however, a monophyletic *Rhinochelys* is a less parsimonious but statistically indistinguishable hypothesis. This uncertainty results, in turn, in unclear implications for the taxonomy of *Rhinochelys*. Indeed, under a monophyletic *Rhinochelys*, the possibility that the differences seen in the taxa and morphotypes of this genus are due to instraspecific variations cannot be ruled out. Yet, the validity and distinctiveness of *R. amaberti*, its close relationships with *R. pulchriceps* and our assessment of the RBINS material suggest the existence of a certain taxonomic diversity within the British material. This material is crucial to understand the radiation of chelonioids and is in need of a thorough re-investigation, which is however beyond the scope of this study.

An unexpected and weakly supported result of our analysis of the chelonioid matrix is the recovery of *Syllomus* as a late surviving protostegid (Fig. 8), although in a basal position within this clade. Such a position outside cheloniids might be plausible as *Syllomus* is a peculiar taxon showing some characters infrequently found in cheloniid (like a highly serrated maxilla, or quadrate not wrapping the stapes (Weems, 1980)). Because of its peculiarities and its Neogene age (where only a few taxa are known) *Syllomus* is however subject to long-branch attraction and its phylogenetic position should be regarded as highly tentative until a thorough reinvestigation of this taxa is attempted. The phylogeny of Mesozoic marine turtles presented here supports the subdivision of most of the marine turtles in three major families (Fig. 8). However, their taxonomic contents are volatile, because of a generalized lack of information in the matrices that are presently available (see also Myers et al., 2017). The datasets we considered agree on the fact that the radiation of chelonioid mainly took place during the Early Cretaceous; a reduction of the cladogenesis rates after the middle Cretaceous is evident and contributed to the erosion of the chelonioid phylogenetic diversity over time. However, poor stratigraphic congruence indexes (as we report in Fig. 9) indicate the presence of long ghost lineages, thus old node ages. While this does not mean that the ages of the nodes are incorrect, a solid phylogenetic hypothesis for the evolution of chelonioids is needed to reconstruct their cladogenesis rate through time with confidence. For all these reasons, it is evident that the phylogeny of chelonioids is in dire need of new data. A thorough osteological re-evaluation of most protostegids (along with *Syllomus*) is therefore necessary to provide a series of new characters. A particular attention should also be given to the operational taxonomic units preserving both the skull and the postcranial skeleton, such as *R. nammourensis*, as these skeletal regions are often mutually exclusive in a series of testudine taxa, resulting in poorly supported phylogenetic hypotheses.

## Conclusions

*Rhinochelys amaberti* is a valid taxon from the late Albian of France that is distinguished by eight autapomorphic features. The generic placement of *R. amaberti* as a distinct species within *Rhinochelys* is supported by anatomical and phylogenetic evidence. We described previously unreported skulls from the late Albian of the United Kingdom, which suggest the presence of two skull types within the genus *Rhinochelys*. We recover *Rhinochelys* as a non-monophyletic entity in two distinct phylogenetic analyses that widely sample protostegids, but these most parsimonious solutions cannot be statistically distinguished from a monophyletic *Rhinochelys*. Our analyses reveal that the phylogeny of chelonioids is unstable and not congruent with stratigraphy and would benefit from increased character sampling. Despite this instability, our analyses of distinct datasets suggest that Chelonioidea evolved rapidly during the Early Cretaceous, thanks to the intense radiation of Protostegidae.

##  Supplemental Information

10.7717/peerj.4594/supp-1 Supplemental Information 1 Supplementary methods and results

10.7717/peerj.4594/supp-2 Dataset S1 Full cladistic dataset

10.7717/peerj.4594/supp-3 Dataset S2 Taxon ages for the chelonioid cladistic dataset

10.7717/peerj.4594/supp-4 Dataset S3 Chelonioid cladistic dataset

10.7717/peerj.4594/supp-5 Dataset S4 Taxon ages for the Bardet cladistic dataset

10.7717/peerj.4594/supp-6 Dataset S5 Bardet cladistic dataset

10.7717/peerj.4594/supp-7 Dataset S6 Bardet full cladistic dataset

10.7717/peerj.4594/supp-8 Supplemental Information 2 Cladogenesis rate for chelonioid dataset in equal Mean cladogenesis rate and standard deviation using all most parsimonious trees arising from the analyse of the chelonioid dataset using an equal optimization of branch lengths

10.7717/peerj.4594/supp-9 Supplemental Information 3 Cladogenesis rate for Bardet dataset in equal Mean cladogenesis rate and standard deviation using all most parsimonious trees arising from the analyse of the Bardet dataset using an equal optimization of branch lengths

10.7717/peerj.4594/supp-10 Supplemental Information 4 Cladogenesis rate for chelonioid dataset in basic Mean cladogenesis rate and standard deviation using all most parsimonious trees arising from the analyse of the chelonioid dataset using a basic optimization of branch lengths

10.7717/peerj.4594/supp-11 Supplemental Information 5 Cladogenesis rate for Bardet dataset in basic Mean cladogenesis rate and standard deviation using all most parsimonious trees arising from the analyse of the Bardet dataset using a basic optimization of branch lengths

10.7717/peerj.4594/supp-12 Supplemental Information 6 List of character state changes supporting nodes for chelonioid dataset

## Institutional abbreviations

IRSNB: Royal Belgian Institute of Natural Sciences, Brussels, Belgium
UJF: Universite Joseph Fourier, Grenoble, France

## Additional Information and Declarations

### Data Availability

The following information was supplied regarding data availability: The cladistic dataset has been provided as a Supplemental File.

## References

[ref-1] Arnaud H (1988). Subsidence in certain domains of southeastern France during the Ligurian Tethys opening and spreading stages. Bulletin de la Société Géologique de France.

[ref-2] Bapst DW (2012). Paleotree: an R package for paleontological and phylogenetic analyses of evolution. Methods in Ecology and Evolution.

[ref-3] Bardet N (1995). Evolution et extinction des reptiles marins au cours du Mésozoïque. Palaeovertebrata.

[ref-4] Bardet N, Jalil N-E, De Lapparent de Broin F, Germain D, Lambert O, Amaghzaz M (2013). A giant chelonioid turtle from the Late Cretaceous of Morocco with a suction feeding apparatus unique among tetrapods. PLOS ONE.

[ref-5] Bell MA, Lloyd GT (2015). Strap: an R package for plotting phylogenies against stratigraphy and assessing their stratigraphic congruence. Palaeontology.

[ref-6] Benton MJ, Spencer PS, Wimbledon WA, Palmer D (1995). British Cretaceous fossil reptile sites. Fossil reptiles of Great Britain.

[ref-7] Bever GS, Lyson TR, Field DJ, Bhullar B-AS (2015). Evolutionary origin of the turtle skull. Nature.

[ref-8] Bréhéret J-G (1997). L’Aptien et l’Albien de la Fosse vocontienne (des bordures au bassin): evolution de la sédimentation et enseignements sur les événements anoxiques. Société géologique du Nord.

[ref-9] Brinkman DB, Aquillon-Martinez MC, De Leon Davila CA, Jamniczky H, Eberth DA, Colbert M (2009). *Euclastes coahuilaensis* sp. nov., a basal cheloniid turtle from the late Campanian Cerro del Pueblo Formation of Coahuila State, Mexico. PaleoBios.

[ref-10] Brinkman DB, Densmore M, Rabi M, Ryan MJ, Evans DC (2015). Marine turtles from the Late Cretaceous of Alberta, Canada. Canadian Journal of Earth Sciences.

[ref-11] Brinkman DB, Hart M, Jamniczky H, Colbert M (2006). *Nichollsemys baieri* gen. et sp. nov, a primitive chelonioid turtle from the late campanian of North America. Paludicola.

[ref-12] Bureau de recherches géologiques et minières (BRGM) (1967). Carte géologique de la France au 1/50.000, feuille No 796-Vif.

[ref-13] Cadena EA, Parham JF (2015). Oldest known marine turtle? A new protostegid from the Lower Cretaceous of Colombia. PaleoBios.

[ref-14] Collins JI (1970). The chelonian *Rhinochelys* Seeley from the Upper Cretaceous of England and France. Palaeontology.

[ref-15] Cope E (1867). On *Euclastes*, a genus of extinct Cheloniidae. Proceedings of the National Academy of Sciences of Philadelphia.

[ref-16] De Lapparent de Broin F, Bardet N, Amaghzaz M, Meslouh S (2014). A strange new chelonioid turtle from the Latest Cretaceous Phosphates of Morocco. Comptes Rendus Palevol.

[ref-17] Elliot DK, Irby GV, Hutchison JH, Callaway JM, Nicholls EL (1997). *Desmatochelys lowi*, a marine turtle from the Upper Cretaceous. Ancient marine reptiles. Chapter 9.

[ref-18] Fischer V, Bardet N, Guiomar M, Godefroit P (2014). High diversity in cretaceous ichthyosaurs from Europe prior to their extinction. PLOS ONE.

[ref-19] Gaffney ES (1975). *Solnhofia parsonsi*, a new cryptodiran turtle from the Late Jurassic of Europe. American Museum Novitates.

[ref-20] Gaffney ES, Meylan PA, Benton MJ (1988). A phylogeny of turtles. The phylogeny and classification of tetrapods.

[ref-21] Garman S (1880). On certain species of Chelonioidae. Bulletin of the Museum of Comparative Zoology at Harvard College.

[ref-22] Gidon M (1977). Carte géologique simplifiée des Alpes occidentales, du Léman à Digne, au 1/250.000.

[ref-23] Goloboff PA, Catalano SA (2016). *TNT* version 1.5, including a full implementation of phylogenetic morphometrics. Cladistics.

[ref-24] Hay OP (1905). On the group of fossil turtles known as the Amphichelydia, with remarks on the origin and relationships of the suborders, superfamilies, and families of Testudines. Bulletin of the American Museum of Natural History.

[ref-25] Hay OP (1908). The fossil turtles of North America. Carnegie Institution of Washington.

[ref-26] Hirayama R (1994). Phylogenetic systematics of chelonioid sea turtles. The Island Arc.

[ref-27] Hirayama R, Callaway JM, Nicholls EL (1997). Distribution and diversity of cretaceous chelonioids. Ancient marine reptiles.

[ref-28] Hirayama R (1998). Oldest known sea turtle. Nature.

[ref-29] Hirayama R, Tong H (2003). *Osteopygis* (Testudines: Cheloniidae) from the Lower Tertiary of the Ouled Abdoun phosphate basin, Morocco. Palaeontology.

[ref-30] Hooks GE (1998). Systematic revision of the Protostegidae, with a redescription of *Calcarichelys gemma* Zangerl, 1953. Journal of Vertebrate Paleontology.

[ref-31] Hopson PM (2005). A stratigraphical framework for the Upper Cretaceous Chalk of England and Scotland with statements on the Chalk of Northern Ireland and the UK Offshore Sector. BSG Research Report.

[ref-32] Jalil N-E, De Lapparent de Broin F, Bardet N, Vacant R, Bouya B, Amaghzaz M, Meslouh S (2009). *Euclastes acutirostris*, a new species of littoral turtle (Cryptodira, Cheloniidae) from the Palaeocene phosphates of Morocco (Oulad Abdoun Basin, Danian-Thanetian). Comptes Rendus Palevol.

[ref-33] Kear BP, Lee MS (2006). A primitive protostegid from Australia and early sea turtle evolution. Biology Letters.

[ref-34] Lehman TM, Tomlinson SL (2004). *Terlinguachelys Fischbecki*, a new genus and species of sea turtle (Chelonioidea: Protostegidae) from the Upper Cretaceous of Texas. Journal of Paleontology.

[ref-35] Lydekker R (1889a). On remains of Eocene and Mesozoic Chelonia and a tooth of (?) *Ornithopsis*. Quarterly Journal of the Geological Society.

[ref-36] Lydekker R (1889b). Catalogue of the fossil Reptilia and Amphibia in the British Museum (Natural history): Part 3, containing the order Chelonia.

[ref-37] Lynch SC, Parham JF (2003). The first report of hard-shelled sea turtles (Cheloniidae sensu lato) from the Miocene of California, including a new species (*Euclastes hutchisoni*) with unusually plesiomorphic characters. Paleobios.

[ref-38] Martill DM, Unwin DM (2012). The world’s largest toothed pterosaur, NHMUK R481, an incomplete rostrum of *Coloborhynchus capito* (Seeley, 1870) from the Cambridge Greensand of England. Cretaceous Research.

[ref-39] Moret L (1935). *Rhinochelys amaberti*, nouvelle espèce de tortue marine du Vraconien de la Fauge près du Villard-de-Lans (Isère). Bulletin de la Societe Geologique de France.

[ref-40] Myers TS, Polcyn MJ, Mateus O, Vineyard DP, Gonçalves AO, Jacobs LL (2017). A new durophagous stem cheloniid turtle from the lower Paleocene of Cabinda, Angola. Papers in Palaeontology.

[ref-41] Nicholson DB, Holroyd PA, Benson R. BJ, Barrett PM (2015). Climate-mediated diversification of turtles in the Cretaceous. Nature Communications.

[ref-42] Owen R (1851). A monograph on the fossil Reptilia of the Cretaceous formations. Monograph of the Palaeontographical society.

[ref-43] Paradis E, Claude J, Strimmer K (2004). APE: analyses of phylogenetics and evolution in R language. Bioinformatics.

[ref-44] Parham JF, Pyenson ND (2010). New sea turtle from the Miocene of Peru and the iterative evolution of feeding ecomorphologies since the cretaceous. Journal of Paleontology.

[ref-45] Pol D, Escapa IH (2009). Unstable taxa in cladistic analysis: identification and the assessment of relevalnt characters. Cladistics.

[ref-46] Seeley HO (1869). Index to the fossil remains of Aves, Ornithosauria and Reptilia, from the Secondary system of strata arranged in the Woodwardian Museum of the University of Cambridge.

[ref-47] Simoes TR, Caldwell MW, Palci A, Nydam RL (2016). Giant taxon-character matrices: quality of character constructions remains critical regardless of size. Cladistics.

[ref-48] Sterli J, Fuente MSDL (2010). Anatomy of *Condorchelys antiqua* Sterli, 2008, and the origin of the modern jaw closure mechanism in turtles. Journal of Vertebrate Paleontology.

[ref-49] Sterli J, Müller J, Anquetin J, Hilger A (2010). The parabasisphenoid complex in Mesozoic turtles and the evolution of the testudinate basicranium. Canadian Journal of Earth Sciences.

[ref-50] Templeton AR (1983). Phylogenetic inference from restriction endonuclease cleavage site maps with particular reference to the evolution of humans and apes. Evolution.

[ref-51] Tong H, Hirayama R, Makhoul E, Escuillie F (2006). *Rhinochelys* (Chelonioidea, Prostostegidae) from the Late Cretaceous (Cenomanian) of Nammoura, Lebanon. Atti della Societa Italiana di Scienze Naturali e del Museo Civico di Storia Naturale de Milano.

[ref-52] Ubaghs C (1875). La *Chelonia hoffmanni*, Gray, du tuffeau de Maestricht. Annales de la Société Géologique de Belgique.

[ref-53] Unwin D (2001). An overview of the pterosaur assemblage from the Cambridge Greensand (Cretaceous) of Eastern England. Fossil Record.

[ref-54] Weems RE (1980). *Syllomus aegyptiacus*, a Miocene Pseudodont Sea Turtle. Copeia.

[ref-55] Weems RE (2014). Paleogene chelonians from Maryland and Virginia. PaleoBios.

[ref-56] Wilpshaar M, Leereveld H, Visscher H (1997). Early Cretaceous sedimentary and tectonic development of the Dauphinois Basin (SE France). Cretaceous Research.

[ref-57] Wyneken J (2001). The anatomy of sea turtles. U.S. Department of Commerce NOAA Technical Memorandum NMFS-SEFSC.

[ref-58] Zangerl R (1953). The vertebrate fauna of the Selma Formation of Alabama. Part III. The turtles of the family Protostegidae. Part IV. The turtles of the family Toxochelyidae. Fieldiana: Geology Memoirs.

[ref-59] Zangerl R (1960). The vertebrate fauna of the Selma Formation of Alabama. Rainer Zangerl. Part V. An advanced cheloniid sea turtle. Fieldiana: Geology Memoirs.

[ref-60] Zhou C-F, Rabi M (2015). A sinemydid turtle from the Jehol Biota provides insights into the basal divergence of crown turtles. Scientific Reports.

