# Supplementary material for: Rhinochelys amaberti Moret (1935), a protostegid turtle from the Early Cretaceous of France
Source: PeerJ. 2018 Apr 10;6:e4594. doi: 10.7717/peerj.4594 (PMC5898427; doi:10.7717/peerj.4594)
Supplement: Supplemental Information 1 [file peerj-06-4594-s001.pdf]

Supplementary information: *Rhinochelys amaberti* Moret [1935], a protostegid turtle from the Early Cretaceous of France

Isaure Scavezzoni and Valentin Fischer
Evolution and Diversity Dynamics Lab, Université de Liège, Liège, Belgium.

1 Taxonomic assignation of Cambridge Greensand Member specimens from IRSNB

1.1 Specimens IRSNB GS64 & IRSNB GS65

We analyzed the collection of RBINS, with a total of 9 skulls, 19 mandibles and 5 post-cranial elements. We referred some of these skulls to the genus *Rhinochelys*, six of them were then used in the phylogenetic analysis. IRSNB GS64 (Fig. S1) and IRSNB GS65 (Fig. S2) are referable to *Rhinochelys* morphotype '*elegans*', with IRSNB GS64 likely being a juvenile specimen. IRSNB GS64 and IRSNB GS65 share the following features with the referred specimens of *Rhinochelys* morphotype '*elegans*' [Moret, 1935, Collins, 1970]: the premaxilla is taller than it is wide [Lydekker, 1889a, Collins, 1970] and shows a straight profile [Collins, 1970]; nasals form a typical semi-circular/oval shape [Lydekker, 1889a, Moret, 1935, Collins, 1970]; the 'T' shape of the frontals (with a short vertical stem anteriorly and practically none posteriorly) and their suture with the nasal [Lydekker, 1889a, Moret, 1935, Collins, 1970]; the frontal-prefrontal suture which starts at the nasal's midheight [Lydekker, 1889a, Moret, 1935, Collins, 1970]; the lateral-dorsal expansion of the maxilla slightly outreaching the nasal fossa, plus the shape and position of its suture with the nasal [Lydekker, 1889a, Collins, 1970]; the slight maxillary bulge [Lydekker, 1889a, Moret, 1935, Collins, 1970] above its flattened sinusoidal sulcus [Collins, 1970]; the flat ventral surface of the premaxilla and maxilla [Collins, 1970] along with a slight premaxillary and maxillary dorsal lifting [Collins, 1970]; the inclination of the nasal-frontal surface (which is close to 45°[Collins, 1970] and does not tend to flatten even after reaching the mid length of the orbital cavity on IRSNB GS65) with respect to the maxilla's ventral surface (even if the maxilla is not complete on IRSNB GS64 and IRSNB GS65). This inclination makes the skull high especially when compared to that of *Rhinochelys pulchriceps* [Lydekker, 1889a,

Collins, 1970] and *Rhinochelys amaberti* [Moret, 1935]; the presence of an inclined sulcus on both frontals forming a 'V' (with an obtuse opening) at their median suture, which then goes on medially down to the nasal fossa (thus drawing a final shape resembling a 'Y') [Collins, 1970]; the orbital and the nasal cavities (only preserved enough on IRSNB GS65) are on the same horizontal level [Collins, 1970]; the narrowness of the skull anterior to the orbits [Lydekker, 1889a, Moret, 1935, Collins, 1970] (this is more specifically visible on IRSNB GS65); in ventral view, the angle between the maxillae (preserved on IRSNB GS65 only) [Collins, 1970]; the relative sizes of the orbital and nasal cavities [Collins, 1970]; the lateral orientation of the orbits (preserved on IRSNB GS65 only) [Lydekker, 1889a, Moret, 1935, Collins, 1970].

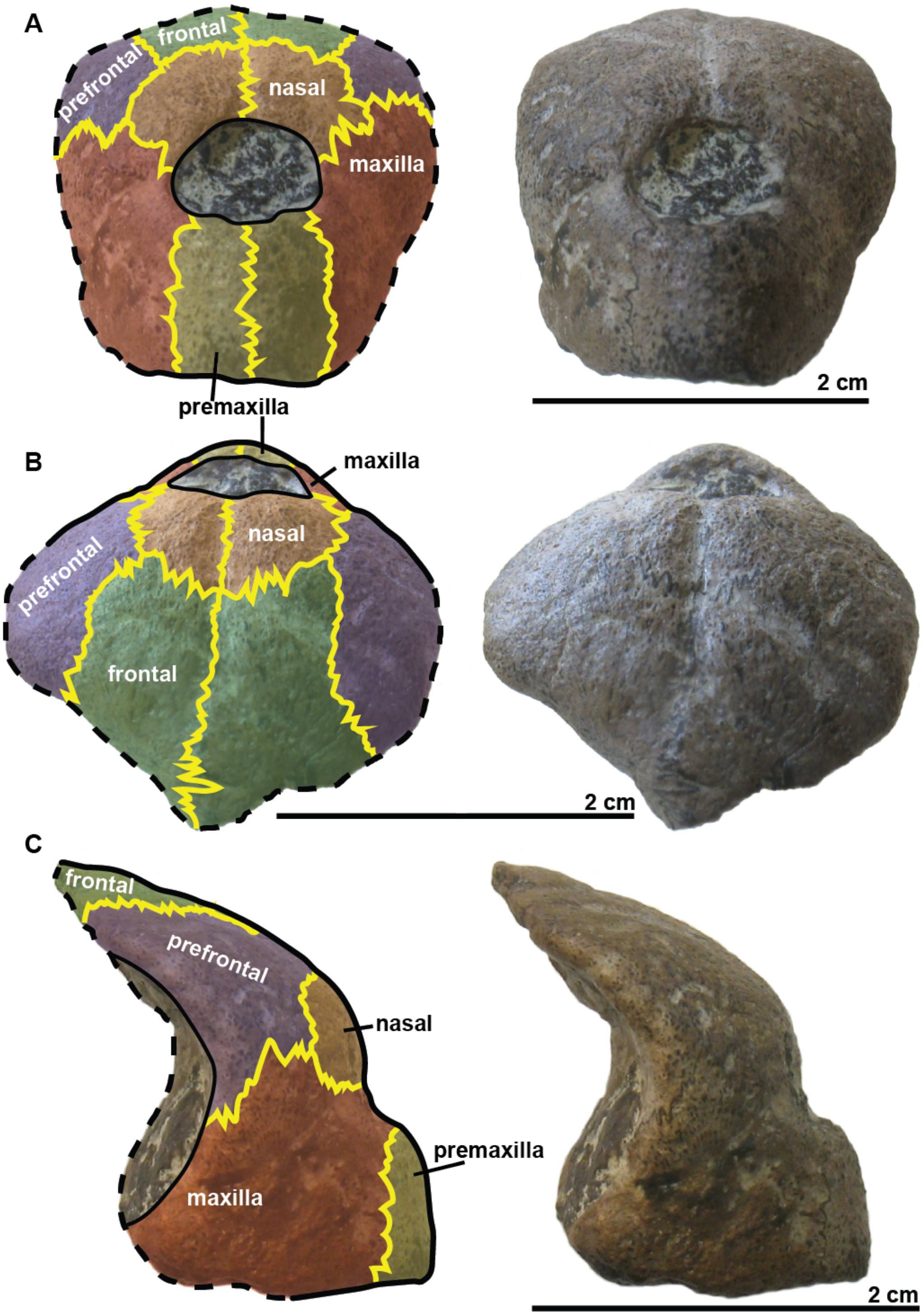

Figure S1: (**A**) Anterior, (**B**) dorsal and (**c**) lateral views of the specimen IRSNB GS64, assigned to *Rhinochelys* morphotype '*elegans*'. Bone sutures are colored in yellow.

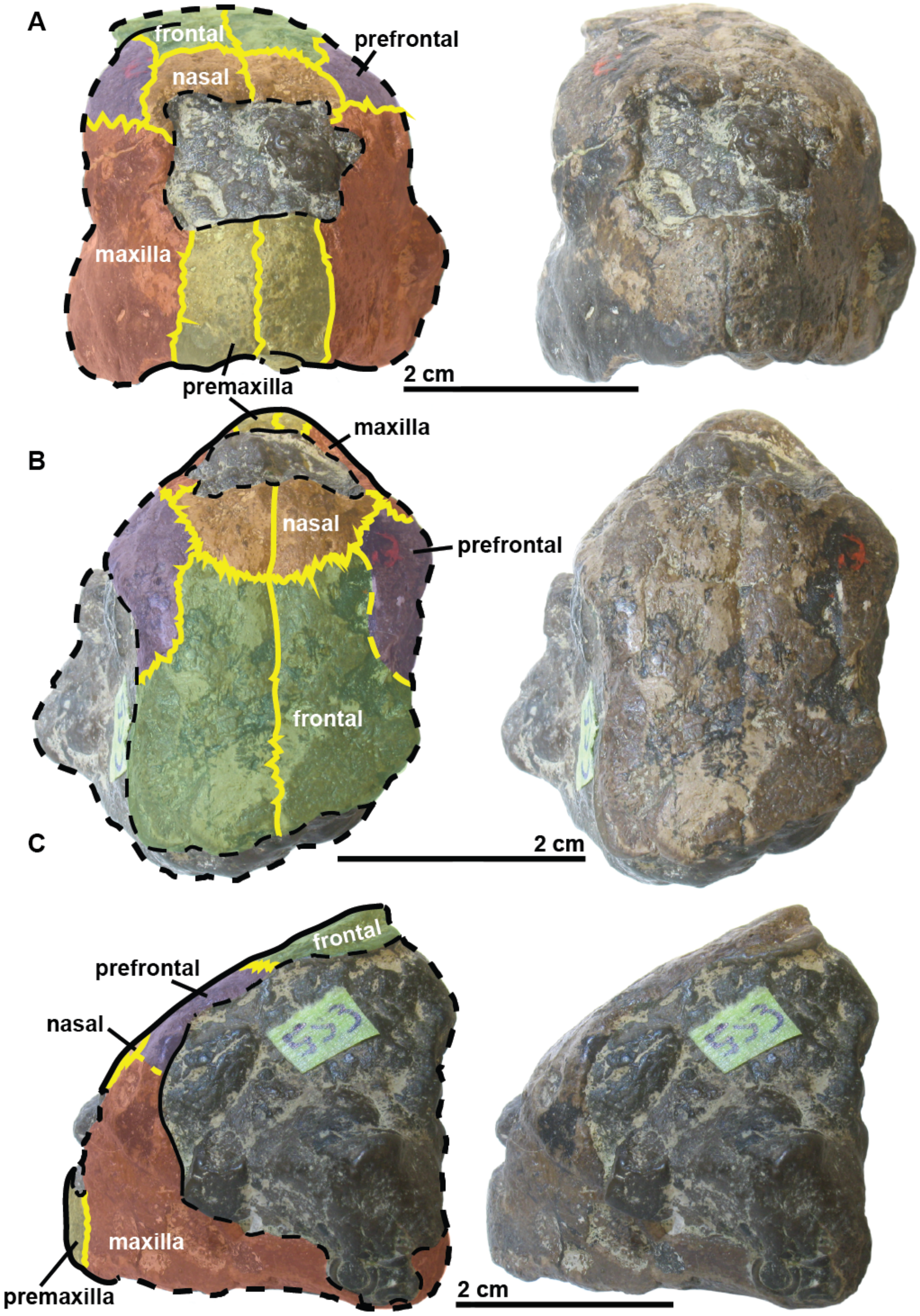

Figure S2: (**A**) Anterior, (**B**) dorsal and (**c**) lateral views of the specimen IRSNB GS65, assigned to *Rhinochelys* morphotype '*elegans*'. Bone sutures are colored in yellow.

1.2 Specimens IRSNB GS68 & IRSNB GS70

IRSNB GS68 (Fig. S3, S4 and Fig. S5) and IRSNB GS70 (Fig. S6) are referable to *Rhinochelys pulchriceps*, with IRSNB GS70 being a juvenile specimen. IRSNB GS68 and IRSNB GS70

share the following features with the referred specimens of *Rhinochelys pulchriceps* [Owen, 1851, Lydekker, 1889a, Moret, 1935, Collins, 1970]: the shape and size of the nasal bone, as well as the length and position on the nasals of their suture with the prefrontal, maxilla and frontal [Owen, 1851, Lydekker, 1889a, Moret, 1935, Collins, 1970]; the cruciform shape of the frontals with their minor participation to the orbital rim [Owen, 1851, Lydekker, 1889a, Moret, 1935, Collins, 1970]; the presence of frontal-parietal and frontal-postorbital sutures [Owen, 1851, Lydekker, 1889a, Moret, 1935, Collins, 1970]; the elongated sinusoidal shape of the frontal-prefrontal suture [Owen, 1851, Lydekker, 1889a, Moret, 1935, Collins, 1970]; the reduced prefrontal expansion [Owen, 1851, Lydekker, 1889a, Moret, 1935, Collins, 1970]; the slender shape of the snout thanks to the maxilla contribution (which also creates an angular profile for the nasal fossa, like the tread of a stair) [Owen, 1851, Moret, 1935, Collins, 1970]; the maxillary bulge (dorsal to its sulcus) which overhangs the labial maxillary margins [Owen, 1851, Lydekker, 1889a, Moret, 1935, Collins, 1970]; the slight dorsal lifting of the ventral surface of the maxilla just posteriorly to its suture with the premaxillary bone [Owen, 1851, Lydekker, 1889a, Moret, 1935, Collins, 1970]; the small size of the nasal fossa [Owen, 1851, Lydekker, 1889a, Moret, 1935, Collins, 1970]; the oval shape and relative dimension of the orbital cavity (only seen on IRSNB GS68) [Owen, 1851, Moret, 1935, Collins, 1970]; the circular shape of the quadrate and its suture with the pterygoid and opisthitic (preserved on IRSNB GS68 only) [Owen, 1851, Moret, 1935, Collins, 1970]; the shape and position of the basioccipital and basisphenoid (preserved on IRSNB GS68 only) [Owen, 1851, Moret, 1935, Collins, 1970]; the absence of a secondary palate (preserved on IRSNB GS68 only) [Owen, 1851, Moret, 1935, Collins, 1970]; palatines meeting medially but that are not fused, forming the outer border of the apertura narium interna (preserved on IRSNB GS68 only) [Owen, 1851, Moret, 1935, Collins, 1970]; the vomer meets the premaxilla and palatin, thus separating the apertura narium interna (preserved on IRSNB GS68 only) [Owen, 1851, Moret, 1935, Collins, 1970]; the presence of a palatine-maxilla suture preventing the pterygoid from meeting the maxilla (preserved on IRSNB GS68 only) [Owen, 1851, Moret, 1935, Collins, 1970]; the narrowness of the pterygoids which are meeting (no space or parasphenoid separating the pterygoids) (preserved on IRSNB GS68 only) [Owen, 1851, Moret, 1935, Collins, 1970]; in ventral view, the angle between both maxillae

is about 70° [Collins, 1970]; there is no ridge over the maxillary sulcus [Owen, 1851, Moret, 1935, Collins, 1970]; the weak inclination of the nasal-frontal and frontal-parietal surfaces with respect to the ventral surface of the maxilla (leading to the low aspect of the skull as in *Rhinochelys pulchriceps* and *Rhinochelys amaberti*) [Owen, 1851, Moret, 1935, Collins, 1970]. The overall shape (not clearly presented on IRSNB GS70) of the skull (small and elongated) is a feature shared with *Rhinochelys pulchriceps*.

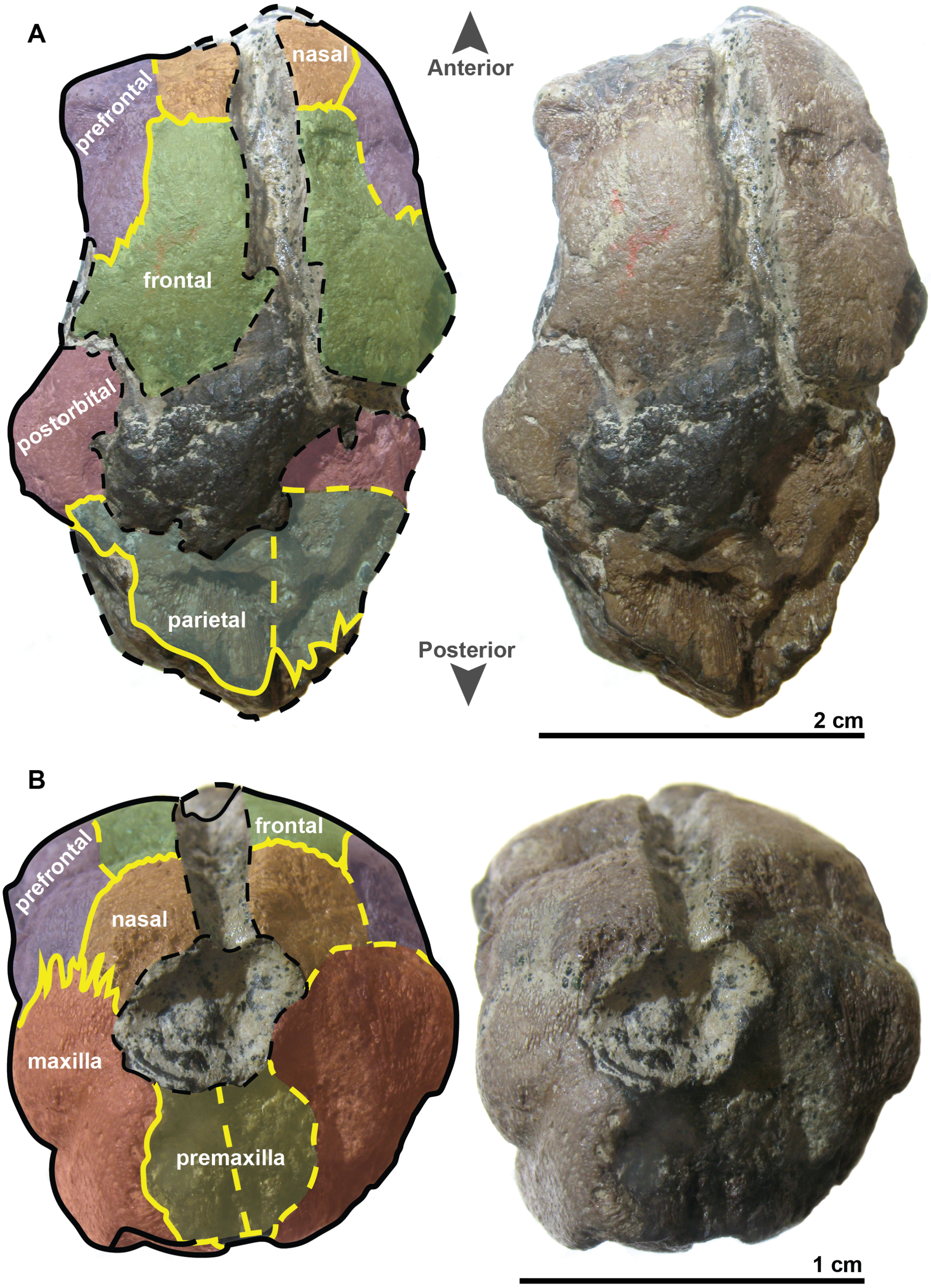

Figure S3: (**A**) Dorsal and (**B**) anterior views of the specimen IRSNB GS68, assigned to *Rhinochelys pulchriceps*. Bone sutures are drawn in yellow plain lines. Yellow dashed lines indicate the hypothetic position of missing bone sutures.

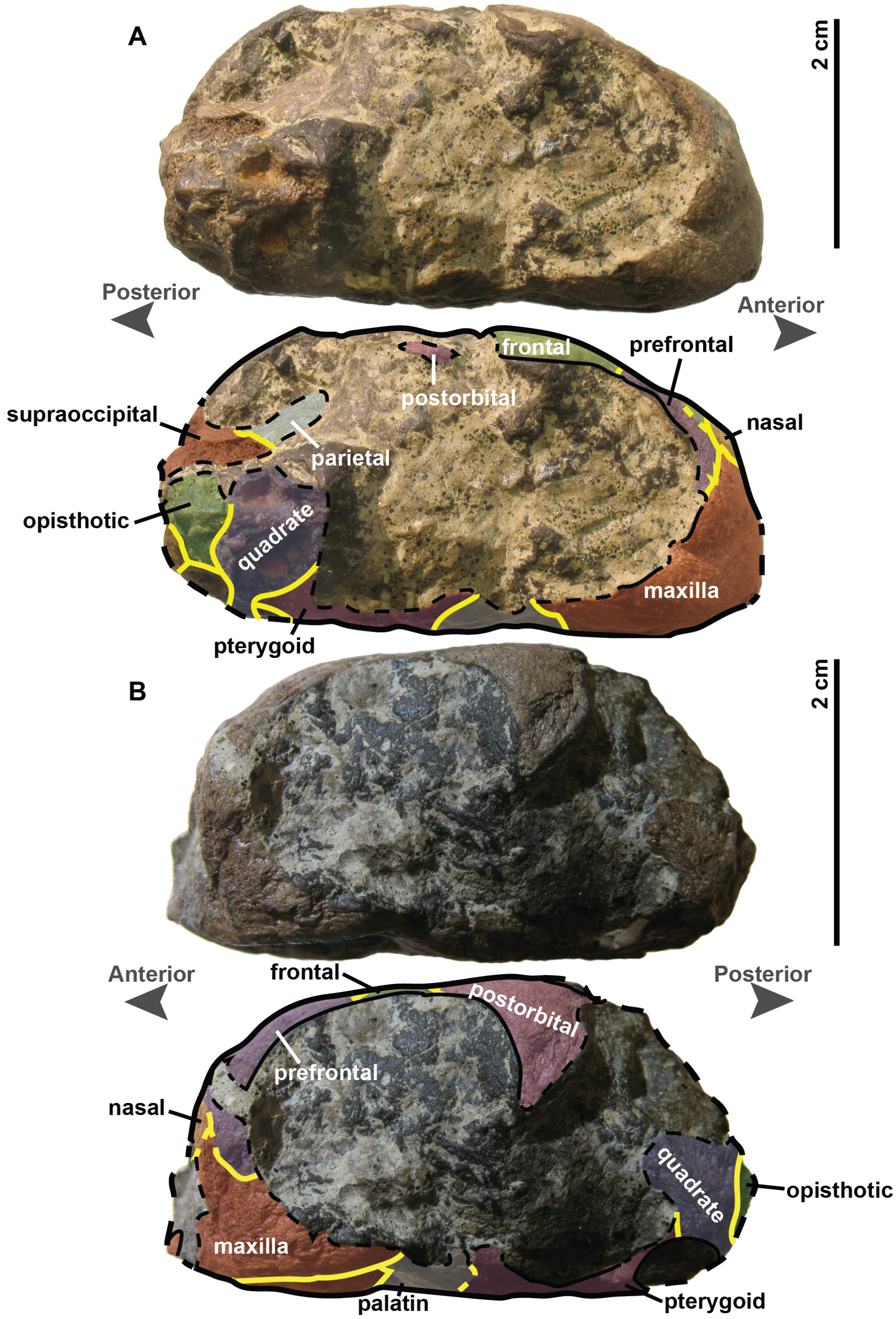

Figure S4: (**A**) Right lateral and (**B**) left lateral views of the specimen IRSNB GS68, assigned to *Rhinochelys pulchriceps*. Bone sutures are drawn in yellow plain lines. Yellow dashed lines indicate the hypothetic position of missing bone sutures.

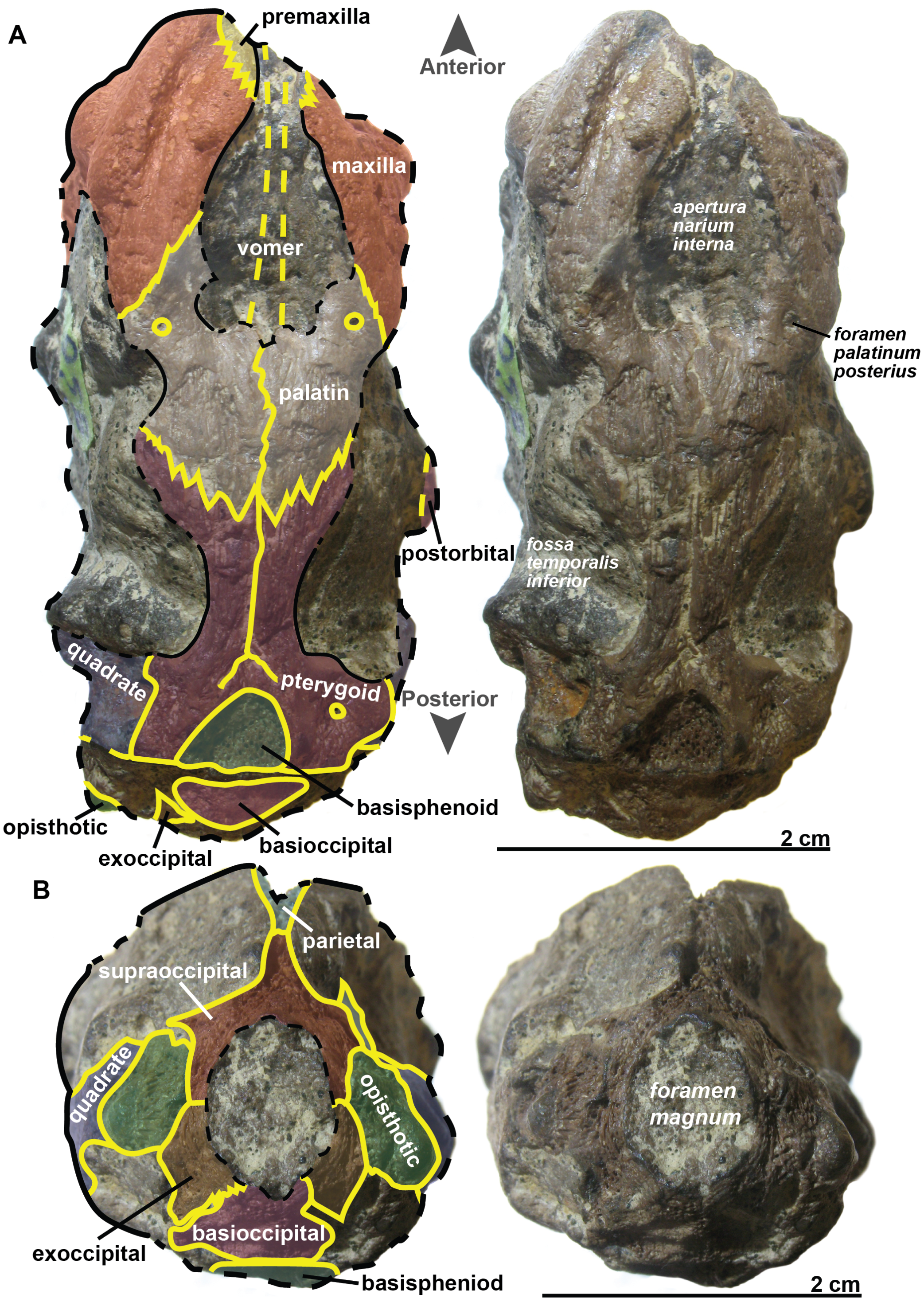

Figure S5: (**A**) Ventral and (**B**) posterior views of the specimen IRSNB GS68, assigned to *Rhinochelys pulchriceps*. Bone sutures are drawn in yellow plain lines. Yellow dashed lines indicate the hypothetic position of missing bone sutures.

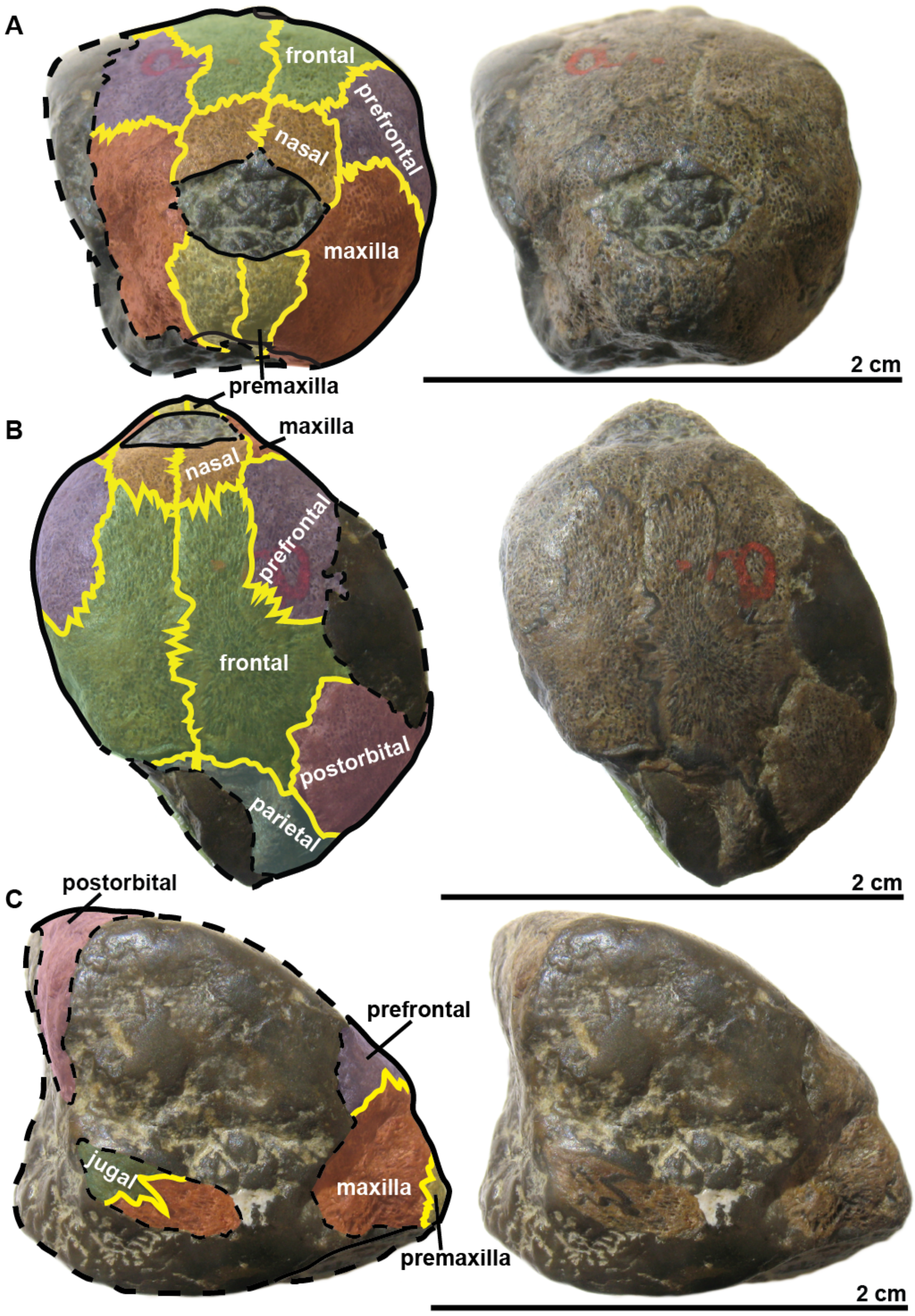

Figure S6: (**A**) Anterior, (**B**) dorsal and (**c**) lateral views of the specimen IRSNB GS70, assigned to *Rhinochelys pulchriceps*. Bone sutures are colored in yellow.

1.3 IRSNB GS63 & IRSNB GS67

IRSNB GS63 (Fig. S7) shares the following features with IRSNB GS67 (Fig. S8): feeble maxillary bulge above the maxillary sulcus; weak indentation of the maxillary sulcus; slight

dorsal lifting of the maxillary labial edge just posteriorly to the premaxillary suture; maxilla extends beyond the bottom edge of the nasal cavity, up to 2/3 of the height of the nasal cavity; the shape of the prefrontal-maxilla suture; the shape and size of the nasal cavity; the shape of the nasal and of the nasal-frontal suture. Therefore, it is likely that IRSNB GS63 and IRSNB GS67 represent the same species.

Given the inclination angle (in relation to the labial edge of the maxilla) of the nasal-frontal surface plus the dorsal exposition of the prefrontal (anteriorly to the maxillary bulge), the complete skull would certainly not resemble that of *Rhinochelys pulchriceps*. The skull of both specimens has a high profile like that of *Rhinochelys* morphotype '*elegans*', *Rhinochelys* morphotype '*cantabrigiensis*', *Rhinochelys* morphotype '*jessoni*', and probably wide like *Rhinochelys* morphotype '*cantabrigiensis*'. The maxillary bulge is feeble, like in *Rhinochelys* morphotype '*cantabrigiensis*' or *Rhinochelys* morphotype '*elegans*', in opposition to *Rhinochelys pulchriceps, Rhinochelys amaberti*, *Rhinochelys* morphotype '*jessoni*'. The maxilla-prefrontal suture does not extend dorsally to the dorsal border of the nasal cavity, as in *Rhinochelys amaberti* and *Rhinochelys* morphotype '*jessoni*' [Lydekker, 1889a, Moret, 1935], and maybe *Rhinochelys* morphotype '*elegans*' [Lydekker, 1889a, Moret, 1935, Collins, 1970]. The shape of the nasal (laterally compressed for antero-posteriorly elongated) and its suture with the frontal and maxilla are similarly observed in *Rhinochelys* morphotype '*jessoni*' (from the drawings of Moret [1935] and description of Lydekker [1889a]). The nasal cavity seems however proportionally larger to the skull than it is for *Rhinochelys* morphotype '*jessoni*' (which could be a juvenile feature). Also the rounded shape of the nasal cavity of IRSNB GS63 and IRSNB GS67 does not correspond to the angular shapes seen in *Rhinochelys* morphotype '*jessoni*' and *Rhinochelys* morphotype '*cantabrigiensis*'. The missing premaxillae are necessary to determine the existence of a beak (as well as its convexity). The maxilla-prefrontal suture is here different and located more ventrally than that of *Rhinochelys* morphotype '*jessoni*' [Moret, 1935]. The slight dorsal lifting of the labial edge of the maxilla seems similar to that of *Rhinochelys* morphotype '*jessoni*' and *Rhinochelys* morphotype '*cantabrigiensis*' which could, however, also be a consequence of erosion or deformation. The shape of the maxilla is similar to *Rhinochelys* morphotype '*cantabrigiensis*' and *Rhinochelys* morphotype '*jessoni*', but the position of the maxillary sulcus

reminds that of *Rhinochelys* morphotype '*cantabrigiensis*' because the sulcus of *Rhinochelys* morphotype '*jessoni*' is located much higher, at the maxilla-prefrontal suture. The shape of the frontal of IRSNB GS67 differs from that of *Rhinochelys* morphotype '*jessoni*', *Rhinochelys* morphotype '*cantabrigiensis*' and *Rhinochelys* morphotype '*elegans*' (however it is similar to that of IRSNB GS64 & IRSNB GS65).

To sum up, IRSNB GS63 and IRSNB GS67 may belong to a new species of *Rhinochelys* and are thus assigned to *Rhinochelys* indet. A thorough re-evaluation of the abundant Cambridge Greensand Member collections of the UK is however needed to confirm this hypothesis.

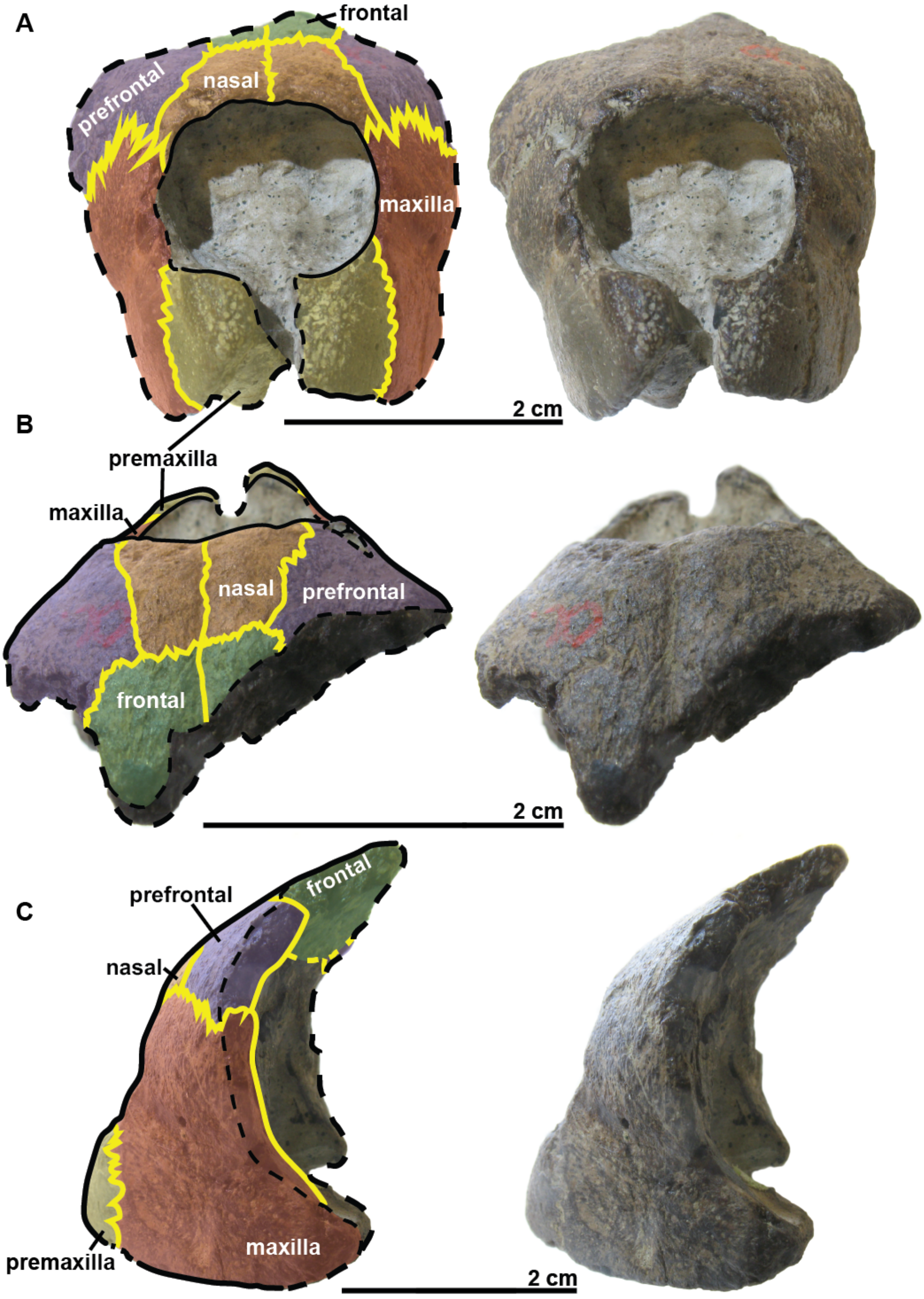

Figure S7: (**A**) Anterior, (**B**) dorsal and (**c**) lateral views of the specimen IRSNB GS63. Bone sutures are colored in yellow. Bone sutures are drawn in yellow plain lines. Yellow dashed lines indicate the hypothetic position of missing bone sutures.

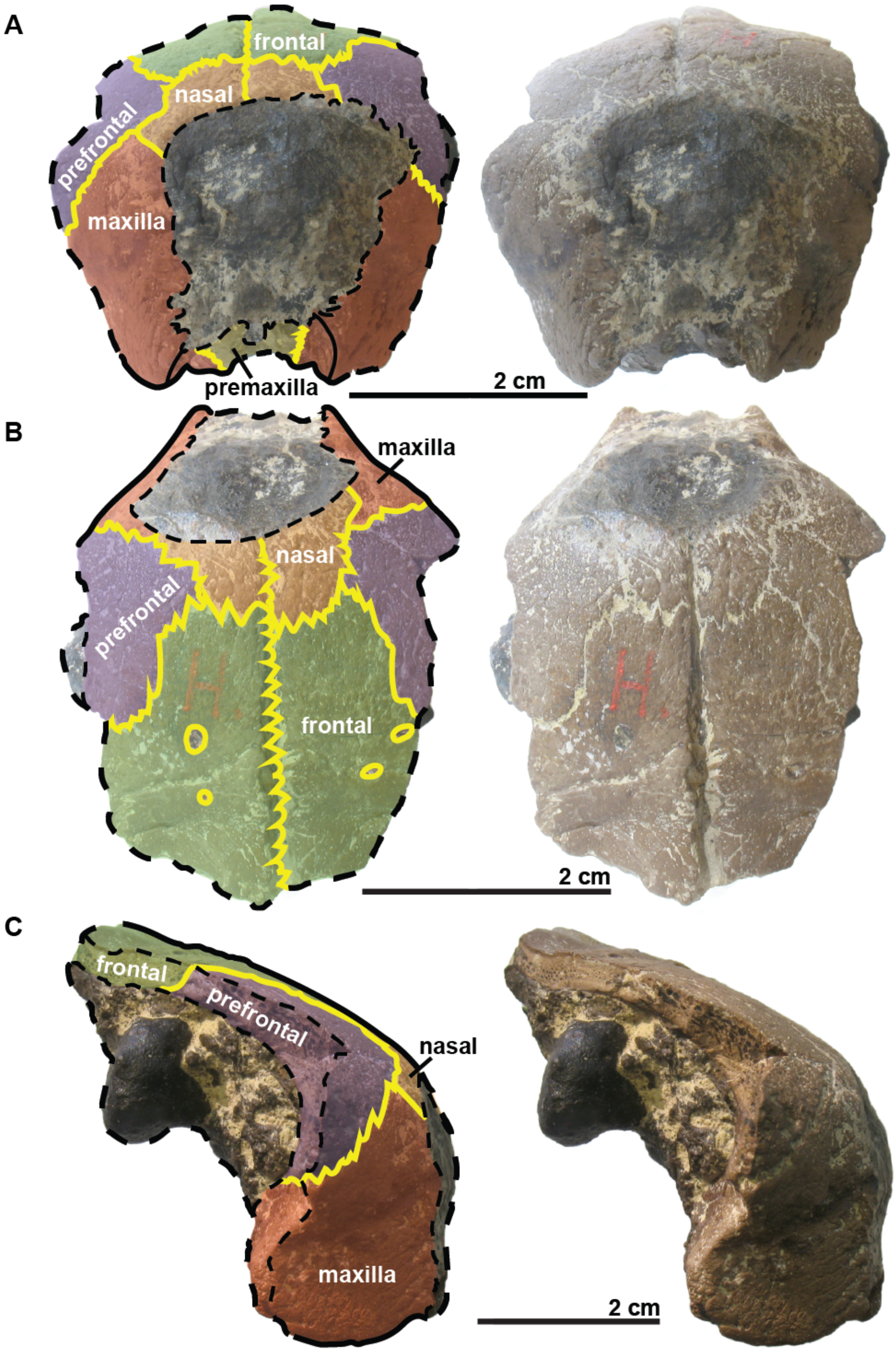

Figure S8: (**A**) Anterior, (**B**) dorsal and (**c**) lateral views of the specimen IRSNB GS67. Bone sutures are colored in yellow.

2 Supplementary phylogenetic results

2.1 'Full matrix'

The cladogram presented on Fig. S9 results from the maximum parsimony analysis in heuristic search analysis of the 'full' matrix in equal weighting. The dataset used comprises the entire set of taxa and characters from Cadena and Parham [2015] plus the new taxa *Rhinochelys amaberti*, *Rhinochelys* morphotype '*cantabrigiensis*', *Rhinochelys* morphotype '*elegans*' and the specimens IRSNB GS63 and IRSNB GS67. The scoring of *Rhinochelys pulchriceps* has also already been modified here.

There is an evident lack of resolution on the cladogram on Fig. S9 as the chelonioids share a polytomy with several terrestrial or semi-aquatic taxa ranging from Mesozoic period to modern days. Such a low resolution is possibly caused by noise from other clades, indeed the molecular data clearly contradict this topology mixing chelonioids with other terrestrial lineages. To cope with this issue, we decided to focus on pelagic taxa and thus removed all but chelonioids and *Solnhofia parsoni* as outgroup. We also noted that as we added our new taxa, the polytomy encompassing marine turtles grew large. This effect was due to the lack of characters to differentiate them (especially for middle Cretaceous taxa). For this reason, we created new characters (see section 4 above). All of these modifications of the original dataset from Cadena and Parham [2015] lead to what we call in this paper the 'chelonioid' dataset.

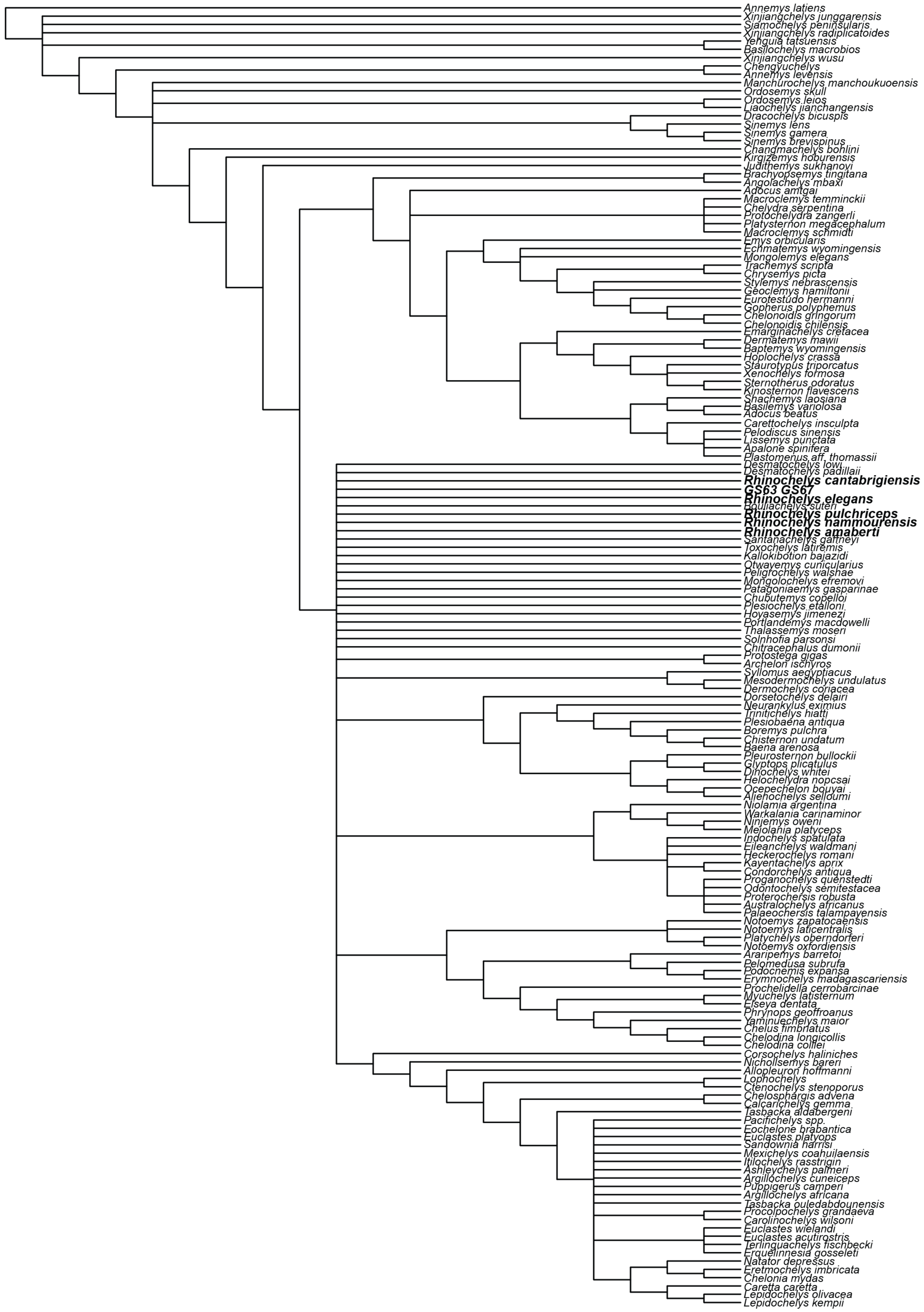

Figure S9: Strict consensus cladogram of 10000 trees (overflow) most parsimonious and 1207 steps long, from a matrix of 256 characters and 157 taxa ('full matrix'). In bold are the taxa we added to the dataset.

2.2 'Chelonioid matrix'

The Cheloniidae family shows several displacements of taxa in our new phylogeny (see Fig. 8 A from main article). *Ashleychelys* becomes the stem cheloniid. The crown-cheloniids form a polytomy in which the extant cheloniids are split in two: *Natator* is moved out of the lineage containing the modern cheloniids. Thus, *Natator* is no longer sister taxon with *Chelonia mydas* [Parham and Pyenson, 2010, Duchene et al., 2012, Cadena and Parham, 2015] or *Syllomus aegyptiacus* [Parham and Pyenson, 2010]. *Caretta* now separates the two species of *Lepidochelys* unlike in Duchene et al. [2012]. The crown-cheloniid lineage also shows these new additions: (1) the former basal cheloniid *Euclastes* unlike in Lynch and Parham [2003], Kear and Lee [2006], Parham and Pyenson [2010], de Lapparent de Broin et al. [2014], Parham et al. [2014]; (2) the former basal cheloniid unlike in Grant-Mackie et al. [2011] or toxochelyid unlike in Tong and Hirayama [2002] *Eochelone* (consequently the lineage containing *Puppigerus, Argillochelys, Tasbacka* and *Eochelone* is dissolved unlike in Tong and Hirayama [2002]); (3) the former basal cheloniid *Itilochelys* unlike in Danilov et al. [2010]; (4) *Mexichelys*, formerly known as *Euclastes coahuilensis* [Brinkman et al., 2009], is moved from Pan-Chelonioidea to Crown-Cheloniidae unlike in Parham and Pyenson [2010]; (5) the former basal cheloniid *Puppigerus* unlike in Weems [1988], Gaffney and Meylan [1988], Kear and Lee [2006], Weems [2014], Cadena and Parham [2015]. Thus *Puppigerus* is not sister to neither *Eochelone* (unlike in Weems [1988], Gaffney and Meylan [1988], Hirayama [1994], Tong and Hirayama [2002]), nor *Chelonia* (unlike in Hirayama [1998], de Lapparent de Broin et al. [2014]), nor *Euclastes* (unlike in Kear and Lee [2006]) anymore. *Pacifichelys* is now sister taxa with *Eochelone* while it supposedly belonged to a Paleogene cheloniid radiation (unlike in Parham and Pyenson [2010]). Consequently, *Argillochelys* loses its affiliation with *Eochelone*, unlike in Weems [1988], Gaffney and Meylan [1988], Hirayama [1994]. This layout reveals a low stratigraphic congruence; we hypothesize that the global relationships of chelonioids is still subject to important changes in the future.

3 Cladogenesis rates

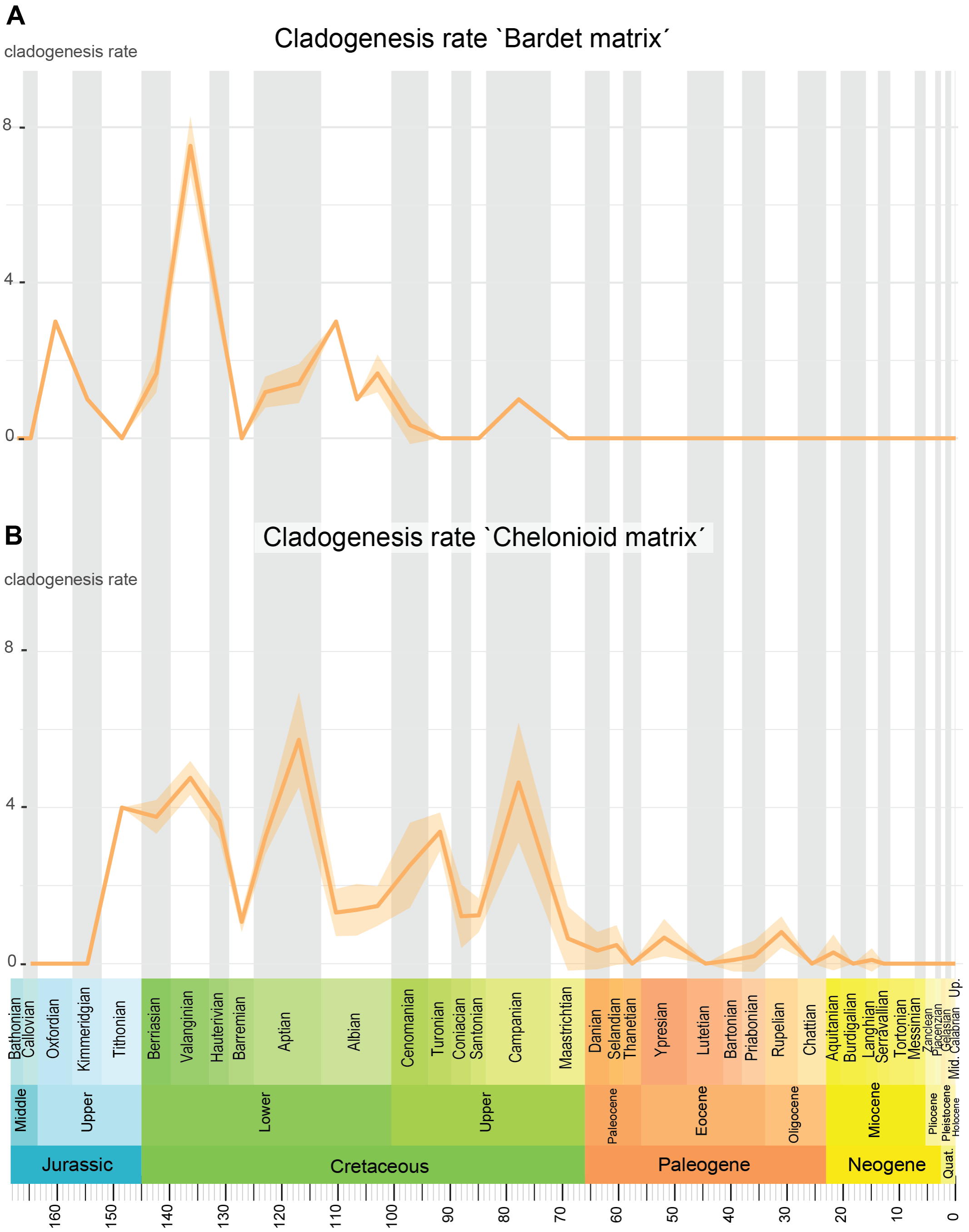

Figure S10: Mean cladogenesis rates and standard deviation using all most parsimonious trees arising from the analyses of **A** the reduced ‘Bardet matrix’, and **B** ‘chelonioid matrix’ using an ’equal’ optimization of branch lengths.

Mean and median cladogenesis rates, as well as their standard deviation were computed using, separately, the ’equal’ and ’basic’ optimizations of branch lengths all most parsimonious trees arising from the analyses of the ‘chelonioid’ and ‘Bardet’ datasets. Cladogenesis events (i.e.

node ages) were binned according to the Mesozoic and Cenozoic stages (or substages for the Norian, Aptian, and Albian, because of the long durations of these stages). The following packages were used to compute and plot the cladogenesis rates: APE [Paradis et al., 2004], PALEOTREE [Bapst, 2012], GGPLOT2 [Wickham, 2009] and RESHAPE2 [Wickham, 2007].

The cladogenesis rates were calculated for both 'chelonioid' and the reduced 'Bardet' matrices using both 'equal' and 'basic' optimizations for branch lengths. The Results using 'equal' optimization are presented in Fig. S10); results from the less informative 'basic' optimization are available in the supplementary information (see Fig. S22). Because this is an a posteriori method to reconstruct branch lengths (and thus node ages), the finer details of these results are expected to be volatile; this is why we solely focus on describing the broader patterns of our results. Both datasets agree on the general shape of the evolutionary radiation of chelonioids, which can be described as follows: the three main families of the Pan-Chelonioidea appeared prior to the Aptian, notably during a first major radiation event that occurred during the first half of the Early Cretaceous. The Protostegidae, which is the first chelonioid family to occur in the fossil record, accounts for the majority of this Early Cretaceous burst. A second peak is recorded in unclear position in mid-Cretaceous; this second peak is associated with the radiation of the derived protostegids, the dermochelyids and the basal cheloniids. A final Cretaceous radiation took place during the Late Cretaceous and is mainly formed by the Crown Cheloniidae clade. Its intensity is greater for the 'chelonioid matrix' as it samples a larger number of cheloniid taxa than the 'Bardet matrix'. It is evident that many chelonioid lineages survive the Cretaceous-Paleogene crisis, but their diversity slowly declines afterwards, as their diversity eroded while only a few novel lineages separated. From these results, it appears clear that most of the radiation of marine turtles is constrained within the Cretaceous. However, we envision that the precise position of cladogenesis peaks is subject to change, when new fossils will improve the topology and stratigraphic congruence of the chelonioid cladogram.

4 List of characters:

4.1 New characters

The following new characters are based on a thorough study of the RBINS specimens (*.i.e*: IRSNB GS63, IRSNB GS64, IRSNB GS65, IRSNB GS67, IRSNB GS68, IRSNB GS70) and the holotype of *Rhinochelys amaberti* (UJF-ID.11167), as well as on schemes and descriptions of Owen [1851], Lydekker [1889a], Moret [1935], Collins [1970], and were created to precise the relationships of middle Cretaceous protostegids.

c257 • Shape of frontal: **0** = frontal lacks anterior processes; **1** = frontal possesses anterior process and the lateral process is anteroposteriorly short (less than 1/3rd of frontal length); **2** = frontal possesses anterior process and the lateral process is anteroposteriorly long (equal or larger than half of frontal length). Note: **1** = *Rhinochelys pulchriceps, Rhinochelys amaberti, Rhinochelys nammourensis*; **2** = *Rhinochelys* morphotype '*elegans*', *Rhinochelys* morphotype '*cantabrigiensis*', *Rhinochelys* morphotype '*jessoni*'.

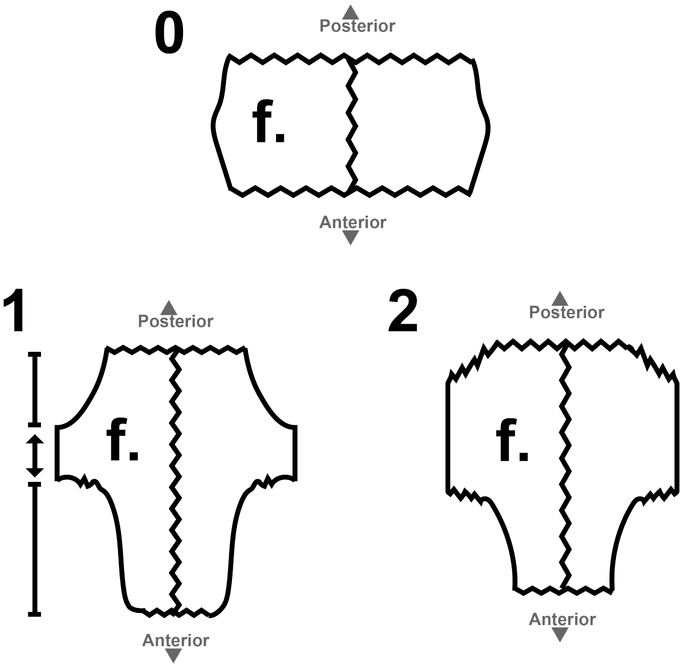

Figure S11: Illustration of the states of character 257.

258 • Shape of the naso-frontal region: **-** (inapplicable) = taxa where nasal bone is absent; **0** = nasal is as long as wide (with a ventral constriction) and straight dorsal margin; **1** = nasal is as long as wide and forms a small medial process separating the frontals medially ; **2** =

nasal is long and narrow (nasal-frontal suture is reduced), and forms a wide expansion that entirely forms the dorsal border of the nasal cavity (thus excluding the prefrontal from the nasal cavity); **3** = nasal is wider than long; **4** = nasal extends laterally beyond the anterior edge of the frontal. Note: **0** = *Rhinochelys pulchriceps*, *Rhinochelys* morphotype '*cantabrigiensis*', *Rhinochelys nammourensis*; **1** = *Rhinochelys* morphotype '*elegans*'; **2/3** = *Rhinochelys amaberti*; **2** = specimens IRSNB GS63 & GS67.

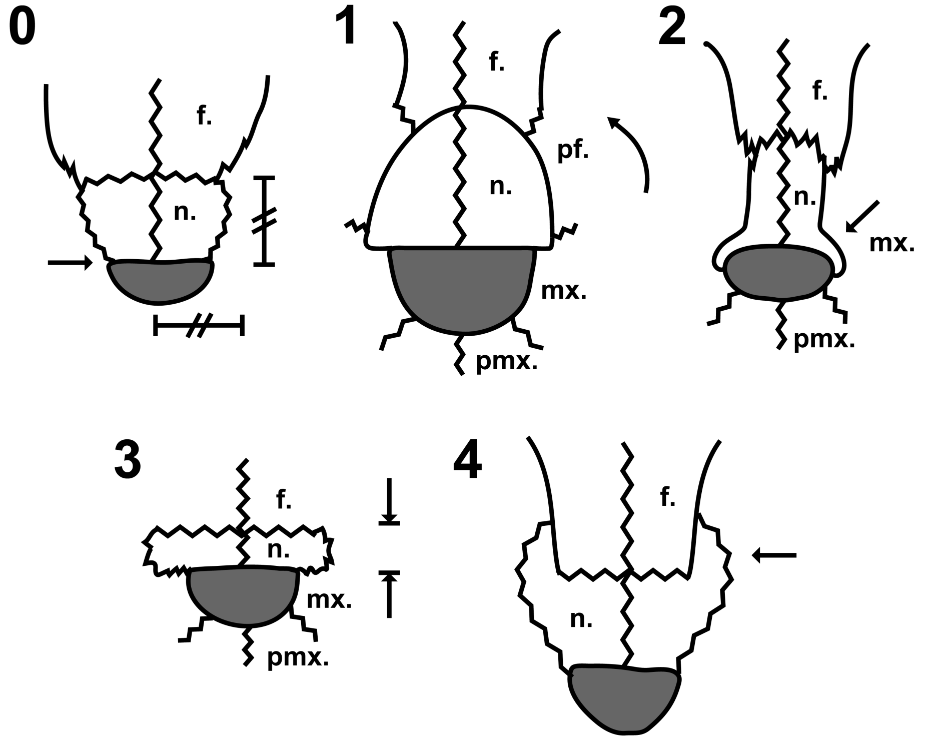

Figure S12: Illustration of the states of character 258.

259 • Posterodorsal extension of the maxilla in relation to the nasal cavity: **0** = the maxilla extends, laterally, beyond the nasal cavity; **1** = the maxilla does not extends beyond the posterior border of the nasal cavity. Note: **0** = *Rhinochelys* morphotype '*cantabrigiensis*', *Rhinochelys amaberti, Rhinochelys nammourensis, Rhinochelys pulchriceps*; **1** = *Rhinochelys* morphotype '*elegans*', *Rhinochelys* morphotype '*jessoni*', specimens IRSNB GS63 & GS67.

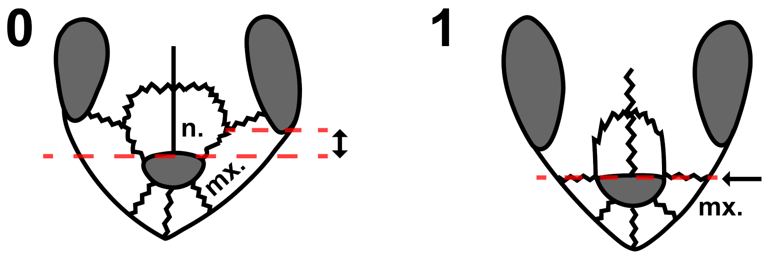

Figure S13: Illustration of the states of character 259.

260 • Labial edge of the maxilla in lateral view: **0** = the labial edge of the maxilla is relatively flat; **1** = the ventral border/labial edge of the maxilla is raised anteriorly (before the premaxilla-maxilla suture). Note: **0** = *Rhinochelys* morphotype '*elegans*', *Rhinochelys amaberti*; **1** = *Rhinochelys pulchriceps*, *Rhinochelys* morphotype '*cantabrigiensis*', specimens IRSNB GS63 & GS67; **?** = *Rhinochelys nammourensis*.

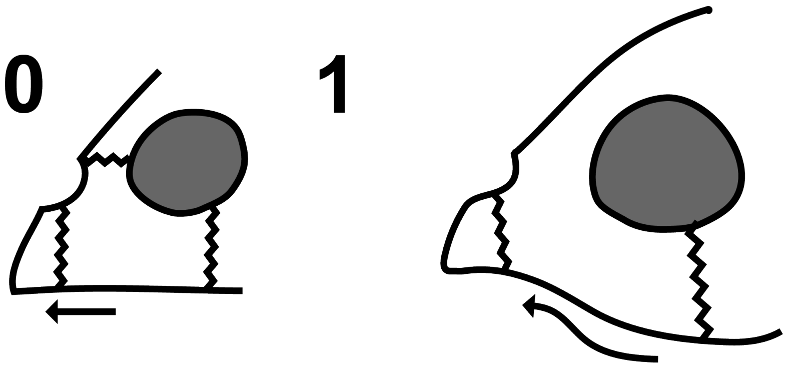

Figure S14: Illustration of the states of character 260.

261 • Maxillary bulge above the maxillary sinusoidal sulcus: **0** = absence of a maxillary bulge; **1** = maxillary bulge is present (just above the maxillary sulcus) but is feeble; **2** = maxillary bulge (just above the maxillary sulcus) is prominent to the point of concealing the labial edge of the maxilla in dorsal view. Note : **1** = *Rhinochelys* morphotype '*cantabrigiensis*', *Rhinochelys* morphotype '*elegans*', specimens IRSNB GS63 & GS67; **2** = *Rhinochelys pulchriceps, Rhinochelys amaberti*.

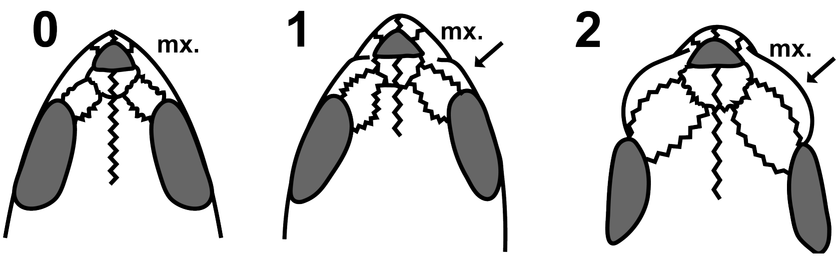

Figure S15: Illustration of the states of character 261.

262 • Position of the orbits with respect to the nasal cavity in lateral view: **0** = the center of the orbit is located dorsally to the level of the center of the nasal cavity; **1** = the center of the orbit and the center of the nasal cavity are located on the same horizontal plan; **2** = the center of the orbit is located ventrally to the center of the nasal cavity. Note: **1** = all species currently assigned to *Rhinochelys*; **?** = *Rhinochelys nammourensis*.

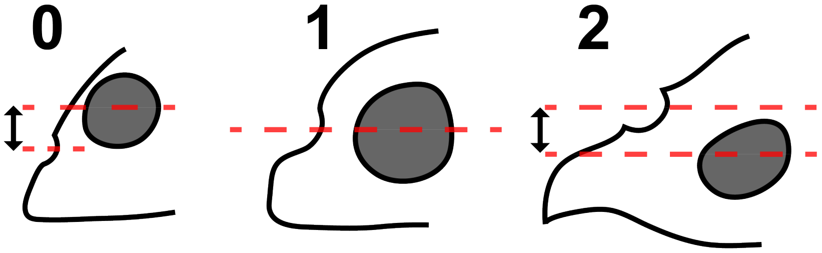

Figure S16: Illustration of the states of character 262.

263 • Skull general shape: **0** = the skull is elevated, the skull table faces antero-dorsally or forms a dome; **1** = the skull is dorsoventrally compressed, the skull table is horizontal. Note: **0** = *Rhinochelys* morphotype '*elegans*', *Rhinochelys* morphotype '*cantabrigiensis*', specimens IRSNB GS63 & GS67; **1** = *Rhinochelys pulchriceps, Rhinochelys amaberti*; **?** = *Rhinochelys nammourensis*.

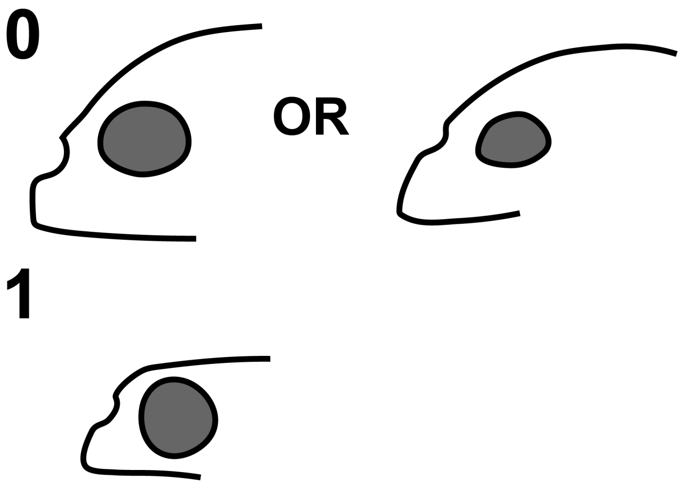

Figure S17: Illustration of the states of character 263.

264 • Orientation of the nasal cavity in dorsal view: **0** = the nasal cavity opens mainly dorsally; **1** = the nasal cavity opens mainly anteriorly. Note: **1** = all species currently assigned to *Rhinochelys*.

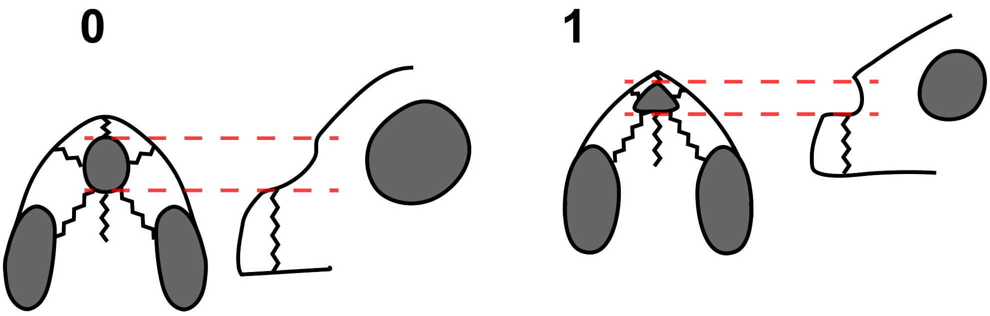

Figure S18: Illustration of the states of character 264.

265 • Concavity of the anterior (external) surface of the premaxilla: **0** = straight (orientated vertically or facing anterodorsally); **1** = convex. Note: **0** = *Rhinochelys* morphotype ‘*elegans*’, *Rhinochelys amaberti*, *Rhinochelys pulchriceps*; **1** = *Rhinochelys* morphotype ‘*cantabrigiensis*’; **?** = *Rhinochelys nammourensis*, specimens IRSNB GS63 & GS67.

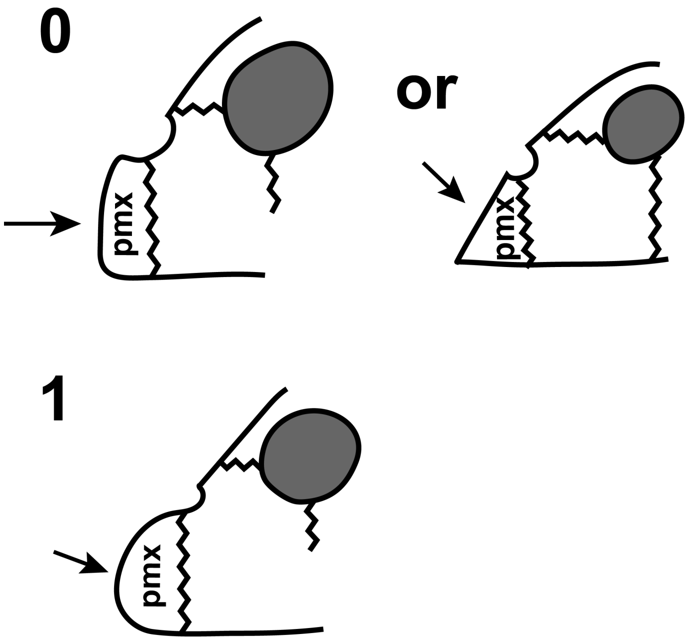

Figure S19: Illustration of the states of character 265.

266 • Relative width of premaxilla: **0** = Mediolaterally narrow, the width of both premaxillae is lesser than the height of one premaxilla ; **1** = Mediolaterally wide, the width of both premaxillae is greater than the height of one premaxilla; **2** = Squared, the width of both premaxillae is neither greater or lesser than the height of one premaxilla. Note: **0** = *Rhinochelys* morphotype '*elegans*'; **1** = *Rhinochelys amaberti, Rhinochelys pulchriceps*; **2** = *Rhinochelys* morphotype '*cantabrigiensis*'; **?** = *Rhinochelys nammourensis*, specimens IRSNB GS63 & GS67.

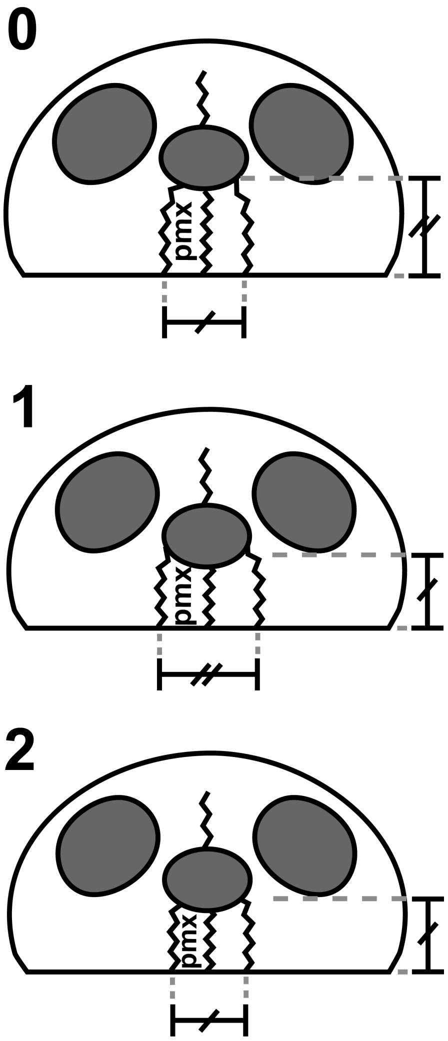

Figure S20: Illustration of the states of character 266.

Revision of character **10** from Cadena and Parham [2015] by adding state 2 and modifying state 1:

10 • ‘Frontal contribution to orbit’: **0** = absent, contact between the prefrontal and postorbital; **1** = frontal shows a minor participation to the orbital rim in comparison to its total length (antero-posteriorly) (*i.e.*, less than 1/3rd of this length); **2** = frontal shows a modest to important participation to the orbital rim. Note: **1** = *Rhinochelys pulchriceps, Rhinochelys amaberti, Rhinochelys nammourensis*; **2** = *Rhinochelys* morphotype ‘*elegans*’, *Rhinochelys* morphotype ‘*cantabrigiensis*’, *Rhinochelys* morphotype ‘*jessoni*’, specimens IRSNB GS63 & GS67.

4.2 Full list of characters

The characters employed in this study are listed here. They are issued from Cadena and Parham [2015], along with our new characters (see section above).

1. Nasals: 0 = present; 1 = absent. JY1 & STF (ch 1, Nasal A). HY2, KL, BR (ch 2).

2. Nasals, medial contact of nasals: 0 = nasals contact one another medially along their entire length; 1 = medial contact of nasals partially or fully hindered by long anterior frontal process. JY1 & STF (ch 2, Nasal B).

3. Nasals, size of nasals: 0 = dorsal exposure of nasals large; 1 = dorsal exposure of nasals greatly reduced relative to that of the frontals. JY1 & STF (ch 3, Nasal C).

4. Prefrontals, medial contact of prefrontals on the dorsal skull surface: 0 = absent; 1 = present, absence of contact between the nasal or apertura narium externa and the frontal. JY1 & STF (ch 4, Prefrontal A), HY2, KL, BR (ch 3).

5. Prefrontals, prefrontal-vomer contact: 0 = present; 1 = absent. JY1 & STF (ch 5, Prefrontal B).

6. Prefrontals, prefrontal-palatine contact: 0 = present; 1 = absent. JY1 & STF (ch 6, Prefrontal C).

7. Prefrontals, dorsal prefrontal exposure: 0 = present, large; 1 = reduced; 2 = absent or near absent. JY1 & STF (ch 7, Prefrontal D). Remarks: Anquetin (2012) splits this character in two: AN (ch 9) & (ch 10) arguing for a better test of the congruence of the lack of a dorsal exposure of prefrontals in the phylogenetic analysis. Ordered.

8. Prefrontals, cranial scutes on the prefrontal: 0 = one pair; 1 = two pairs or more. PH (ch 10); HY2 & KL (ch 1); BR (ch 1). Remarks: State 1 modifed considering the presence of more than two pairs of scutes in *Eretmochelys imbricata* and *Lepidochelys kempii*.

9. Lacrimal: 0 = present; 1 = absent. JY1 & STF (ch 9, Lacrimal A).

10. Frontals, frontal contribution to orbit (modified from Cadena and Parham [2015]): 0 = absent, contact between prefrontal and postorbital; 1 = frontal shows a minor participation to the orbital rim in comparison to its total length (antero-posteriorly) (*i.e.*, less than 1/3rd of this length); frontal shows a modest to important participation to the orbital rim. Note: **1** = *Rhinochelys pulchriceps, Rhinochelys amaberti, Rhinochelys nammourensis*; **2** = *Rhinochelys* morphotype '*elegans*', *Rhinochelys* morphotype '*cantabrigiensis*', *Rhinochelys* morphotype '*jessoni*', specimens IRSNB GS63 & GS67.

11. Frontals, both frontals medially fused: 0 = absent; 1 = present. Bona and de la Fuente (2005) and STF (ch 11, Frontal B).

12. Frontals, direction of the orbits in dorsal view of the skull: 0 = laterally facing, with a very narrow to almost complete absent dorsal exposure of the maxilla and jugal; 1 = dorsolateral facing, with portions of the maxilla and jugal dorsally exposed. Modifed from PH (ch 12); HY2, KL, BR (ch 5).

13. Parietals, parietal-squamosal contact: 0 = present, upper temporal emargination absent or poorly developed; 1 = absent, upper temporal emargination well developed. JY1 (ch 11) & STF (ch 12) (Parietal A); HY2, KL, BR (ch 7). Remarks: Ocepechelon and Archelon have a very narrow contact. Also the outline in *Alienochelys* is not complete and a very narrow contact could also be possible.

14. Parietals, closure of foramen nervi trigemini and the length of the anterior extension of the lateral braincase wall: 0 = foramen nervi trigemini anteriorly open, anterior extension of lateral braincase wall absent; 1 = foramen nervi trigemini anteriorly closed, processus inferior parietalis only produces a narrow strut anterior to the foramen nervi trigemini, usually absence of contact with palatine; 2 = foramen nerivi trigemini anteriorly closed, processus inferior parietalis produces an extended process anterior to the foramen nervi trigemini, contact with palatine commonly present. The character states of JY1 (ch 12) & STF (ch 13) (Parietal B) and JY1 (ch 13) & STF (ch 14) (Parietal C); HY2, KL, & BR (ch 6) form a logical morphocline and we therefore combine them into a single multistate character. Remarks: coded for few fossils because: poorly described specimens, lack of

fgures detailing this feature, or obscured by rock matrix.

15. Parietals, posterodorsal margin of the temporal fossa roofed by an overhanging process of the skull roof: 0 = absent; 1 = present. JY2 (ch 14) & STF (ch 15) (ParietalD).

16. Parietals, contribution to the processus trochlearis oticum: 0 = absent; 1 = present. Meylan and Gaffney (1989), STF (ch 17, Parietal F).

17. Parietals, foramen stapedio-temporalis: 0 = absent or weak, foramen stapedio-temporale concealed in dorsal view; 1 = moderate foramen stapedio-temporale, partial exposition of the processes trochlearis in dorsal view; 2 = strong, entire exposition of the processus trochlearis in dorsal view. STF (ch 19, Parietal H). Ordered.

18. Parietals, pineal foramen located medially between parietals: 0 = absent; 1 = present. New character.

19. Jugals, jugal-squamosal contact: 0 = present; 1 = absent, contact between postorbital and quadratojugal present. JY1 (ch 14) & STF (ch 20) (Jugal A); HY1 (ch 8).

20. Jugals, jugal participation in the rim of the upper temporal emargination: 0 = absent; 1 = present, upper temporal emargination extensive. JY1 (ch 15) & STF (ch 21) (Jugal B).

21. Jugals, jugal-quadrate contact: 0= absent; 1= present, quadratojugal does not contribute to lower temporal margin. HY2, KL & BR (ch 9).

22. Jugals, medial process of jugal beneath orbit, seen in ventral view to slightly ventroposterior view: 0 = weakly developed or absent, jugal only contacts the maxilla; 1 = weak to moderately developed, presence of a contact between the jugal and pteygoid due to the lateral extension of this last; 2 = strongly developed, jugal contacts the pterygoid, the palatine, and the maxilla. Combined and modifed from HY2, KL & BR (ch 10 and ch 11). Remarks: Ventral view is not always precise enough to see the contact, so there might be some specimens for which there is a contact between the palatine and the jugal but it is slightly or completely hidden by the maxilla or the palatine, that is why we included the observation of ventroposterior view of the skull in the defnition of this character. Ordered.

23. Quadratojugals, deep lower temporal emargination extending above the upper limit of the cavum tympani and the resulting loss of the quadratojugal: 0 = absent; 1 = present. Reworded from JY1 (ch 16) & STF (ch 22) (Quadratojugal A) and AN (ch 22); HY2, KL & BR (ch 12).

24. Quadratojugals, quadratojugal-maxilla contact: 0 = absent; 1 = present, jugal does not contribute to lower temporal emargination. JY1 (ch 17) & STF (ch 23) (Quadratojugal B).

25. Quadratojugals, quadratojugal-squamosal contact below the cavum tympani: 0 = absent; 1 = present. JY2 (ch 19) & STF (ch 24) (Quadratojugal C) and AN (ch 24).

26. Squamosals, squamosal-postorbital contact: 0 = present; 1 = absent, temporal roofng well developed, but postorbital short; 2 = absent, due to lower temporal emargination; 3 = absent, due to upper temporal emargination. JY1 (ch 18) & STF (ch 25) (Squamosal A). Remarks: Anquetin (2012) omitted this character, however we do not share his concerns in regard to this character and maintain it as developed by Joyce (2007).

27. Squamosals, squamosal-supraoccipital contact: 0 = absent; 1 = present. JY1 (ch 19) & STF (ch 26) (Squamosal B).

28. Squamosals, posterolateral protuberances developing horns: 0 = absent; 1 = present. Gaffney (1996), STF (ch 27, Squamosal C).

29. Squamosals, very long posterior process, formed exclusively by the squamosal and protruding beyond condyles occipitalis: 0 = absent; 1 = present. Gaffney et al. (2006) & STF (ch 28, Squamosal D).

30. Squamosals, squamosal-quadrate contact: 0 = tightly sutured; 1 = wide open. STF (ch 29, Squamosal E).

31. Postorbitals, postorbital-palatine contact: 0 = absent; 1 = present, foramen palatinum posterius situated posterior to the orbital wall. JY1 (ch 20) & STF (ch 30) (Postorbital A).

32. Supratemporal: 0 = present; 1 = absent. JY1 (ch 21) & STF (ch 31) (Supratemporal A).

33. Premaxilla, subdivision of the apertura narium externa by an internarial process of the premaxilla only: 0 = present; 1 = absent. JY2 (ch 24) & STF (ch 32) (Premaxilla A).

34. Premaxilla, fusion of premaxillae: 0 = absent; 1 = present. JY1 (ch 23) & STF (ch 33) (Premaxilla B).

35. Premaxilla, foramen praepalatinum: 0 = present; 1 = absent; 2 = absent, foramen intermaxillaris present. JY1 (ch 24) & STF (ch 34) (Premaxilla C); HY2, KL & BR (ch 14). Anquetin (2012) modifed this character from multistate to binary, however we do not follow the logic of Anquetin and maintain it as developed by Joyce (2007).

36. Premaxilla, exclusion of the premaxillae from the apertura narium externa: 0 = absent; 1 = present. JY1 (ch 25) & STF (ch 35) (Premaxilla D).

37. Premaxilla, distinct, medial premaxillary hook along the labial margin of the premaxillae: 0 = absent; 1 = present. JY1 (ch 26) & STF (ch 36) (Premaxilla E); HY2,KL & BR (ch 13).

38. Palatines, palatine contribution to the anterior extension of the lateral braincase wall: 0 = absent; 1 = present, well-developed. JY1 (ch 30) & STF (ch 48) (Palatine A).

39. Palatines, contribution to the upper triturating surface: 0 = absent or less than 30% of the total width of the triturating surface; 1 = present, at least 30% or more of the total width of the triturating surface. Modifed from HY2, KL, BR (ch 15), STF (ch 38, Maxilla B).

40. Palatines, secondary palate: 0 = absent; 1 = present, complete separation of the narial cavity from the oral cavity. PH (ch 1); BR (ch 15) & STF (ch 39, Maxilla C).

41. Palatines, vomer-palatine contact anterior to internal naris (apertura narium interna): 0 = absent; 1 = present. HY2, KL, & BR (ch 18). Remarks: Character visible in ventral/palatal view.

42. Maxilla, triturating surface defnition: 0 = triturating surface with labial ridge only; 1 = triturating surface with labial and lingual ridge; 2 = triturating surface with labial, lingual, and accessory ridge(s). AN (ch 38); HY2, KL, & BR (ch 19); STF (ch 40, Maxilla D).

Remarks: Sterli and de la Fuente (2013) coded Chelydra serpentina and Caretta caretta as lacking a lingual ridge (0), but the lingual ridge is present in both taxa, coded here as (1). Ordered.

43. Maxilla, accessory ridge(s): 0 = accessory ridge(s) on maxilla present along the triturating surface; 1 = accessory ridge(s) only in some sectors of the triturating surface. Gaffney (1992); STF (ch 41, Maxilla E).

44. Vomer, number of vomer(s): 0 = paired; 1 = single, but large; 2 = single and greatly reduced or absent. JY1 (ch 26) & STF (ch 42) (Vomer). Remarks: Anquetin (2012) split the character in two AN (ch 41 and ch 42), however we prefer to keep this character as unique and multistate.

45. Vomer, vomer-pterygoid contact in palatal view: 0 = present; 1 = absent, medial contact of palatines present. JY1 (ch 28) & STF (ch 43) (Vomer B); HY2, KL & BR (ch 20).

46. Vomer, vomerine and palatine teeth: 0 = present; 1 = absent. JY1 (ch 29) & STF (ch 44) (Vomer C).

47. Vomer, vomer-premaxilla contact in ventral view: 0 = broad, anterior margin of the vomer straight; 1 = very reduced, anterior margin of vomer forming an acute tip; 2 = absent, both maxilla meeting medially. ST (ch 31); PH (ch 4 and ch 9); STF (ch 45, Vomer D). Remarks: Chelonia mydas is coded here as (0) afer direct examination of specimens (see Table 1S). Tis character strictly deals with the contact on ventral surface of the skull; a premaxilla-vomer contact can be absent in ventral view but present dorsoanteriorly inside the palate. Ordered.

48. Vomer, ventral crest: 0 = absent; 1 = narrow and tall ventral crest present all along the vomer. Reworded from STF (ch 46, Vomer E).

49. Vomer, shape of the palate roof: 0 = flat; 1 = domed. Reworded from Gaffney (1983) and STF (ch 47, Vomer F). Remarks: coded for few fossils because: poorly described specimens, lack of fgures detailing this feature, or obscured by rock matrix.

50. Vomer, vomerine pillar visible in ventral view: 0 = vomerine pillar absent; 1 = present; 2 = present but obscured in ventral view by the posterior extension of the triturating surface of the vomer. Modifed from PH (ch 2); HY2, KL, & BR (ch 17). Remarks: An additional state was added for the absence of the pillar. Ordered.

51. Vomer, contribution to the upper triturating surface; 0 = absent, triturating surface narrow to absent; 1 = present. HY2, KL, & BR (ch 16).

52. Quadrates, flooring of cavum acustico-jugulare and recessus scale tympani: 0 = absent; 1 = fully or partially present, produced by the posterior process of the pterygoid, but the pterygoid does not cover the prootic; 2 = produced by the posterior process of the pterygoid, and the pterygoid covers the prootic; 3 = fully or partially present, produced by the ventral process of the quadrate or the prootic, or both. JY1 (ch 31) & STF (ch 49) (Quadrate A). Remarks: Anquetin (2012) redefned this character to make binary, however we to keep this character as multistate defned by Joyce (2007).

53. Quadrates, development of the cavum tympani: 0 = shallow, but not developed anteroposteriorly; 1 = shallow, but anteroposteriorly developed; 2 = deep and anteroposteriorly developed. JY1 (ch 32 and ch 33, Quadrate B and C), STF (ch 50, Quadrate B+C). Ordered.

54. Quadrates, precolumellar fossa: 0 = absent; 1 = present. JY1 (ch 34) & STF (ch 51) (Quadrate D).

55. Quadrates, antrum postoticum: 0 = absent; 1 = present, quadrate does not fully enclose the anterior perimeter of the antrum; 2 = present, quadrate fully encloses the anterior perimeter of the antrum. JY1 (ch 35, Quadrate E), STF (ch 53, Antrum postoticum A). Remarks: we do not follow Sterli (2008) or Anquetin (2012) and retain this character as originally worded by Joyce (2007). Ordered.

56. Quadrates, arrangement between the quadrate, ophisthotic, stapes and Eustachian tube: 0 = the quadrate and the opisthotic form an angle of 90 degrees in lateral view; 1 = present, but the quadrate and the opisthotic form an angle less than 90 degrees in lateral view; 2 = the quadrate is well developed posteroventrally enclosing only the stapes; 3 = the quadrate

is well developed posteroventrally enclosing the stapes and the Eustachian tube; 4 = the quadrate enclosing stapes and the Eustachian tube helped by the posteroventral projection of the squamosal and posterior of the quadratojugal. Modifed from JY2 (ch 37) & STF (ch 53) (Quadrate F). Remarks: we don't follow the rationale of Anquetin (2012) against the usage of multistate characters and recombine AnquetinâĂŹs characters 52, 53, and 54 back into one multistate character, as was done by Joyce (2007) and Sterli (2008). We furthermore do not follow Anquetin's (2012) rationale in regards to the scoring of *Meiolania platyceps*, as this taxon is similar to pleurodires in that the incisura is not close by the quadrate itself, but rather more superfcially by the squamosal, postorbital, and quadratojugal. We nevertheless accept Anquetin (2012) adjustment of Joyce (2007) scoring for *Dinochelys whitei*.

57. Quadrate, processus trochlearis oticum: 0 = absent; 1 = present, very reduce; 2 = present, large forming a well defned musculatory facet. Modifed from STF (ch 54, Quadrate G). Remarks: a third state is added here for those turtles with a very large processus trochlearis oticum. Ordered.

58. Quadrate, contribution to the musculatory facet of the processus trochlearis oticum: 0 = extensive contribution; 1 = small contribution, facet formed principally by the protic and/or parietal. Reworded from Meylan (1987) and STF (ch 55, Quadrate H).

59. Quadrate, qudrate-basiphenoid contact: 0 = absent; 1 = present. Lapparent de Broin and Werner (1998); Gffaney et al. (2006) (ch 104); STF (ch 56, Quadrate I).

60. Epipterygoids: 0 = present, rod like; 1 = present, laminar; 2 = absent. JY2 (ch 37) & STF (ch 57) (Epipterygoid A). Remarks: coded for few fossils because: poorly described specimens, lack of fgures detailing this feature, or obscured by rock matrix.

61. Pterygoids, pterygoid teeth: 0 = present; 1 = absent. JY1 (ch 38) & STF (ch 58) (Pterygoid A).

62. Pterygoids, basipterygoid process and basipterygoid articulation: 0 = basipterygoid process present with a movable basiptergoid articulation; 1 = basipterygoid process present

with a sutured basipterygoid articulation; 2 = basipterygoid process absent and sutured basipterygoid articulation. ST (ch 41), STF (ch 59, Pterygoid B). Remarks: coded for few fossils because: poorly described specimens, lack of fgures detailing this feature, or obscured by rock matrix.

63. Pterygoids, interpterygoid vacuity: 0 = triangular in shape; 1 = reduced to an interpterygoid slit; 2 = reduced to a paired foramen caroticum laterale. JY1 (ch 40) & STF (ch 60) (Pterygoid C). Remarks: coded for few fossils because: poorly described specimens, lack of fgures detailing this feature, or obscured by rock matrix. Ordered.

64. Pterygoids, pterygoid-basioccipital contact: 0 = absent; 1 = present. JY1 (ch 41) & STF (ch 62) (Pterygoid D).

65. Pterygoids, processus trochelaris pterygoideus: 0 = absent; 1 = present. JY1 (ch 42) & STF (ch 63) (Pterygoid E).

66. Pterygoids, foramen palatinum posterius: 0 = present; 1 = present, but open laterally; 2 = absent. JY1 (ch 43) & STF (ch 64) (Pterygoid F); HY2, KL, & BR (ch 21). Remarks: we do not follow the rationale of Anquetin (2012) against the usage of multistate characters and retain this as a multistate character. We nevertheless accept Anquetin (2012) adjustment of Joyce (2007) scoring for Sandownia harrisi. Ordered.

67. Pterygoids, medial contact of pterygoid: 0 = present, pterygoids in a very long medial contact with one another, longer than the basisphenoid total length in midline; 1 = present, pterygoids in medial contact with one another, contact length equal or shorter than the basisphenoid total length in midline; 2 = absent, contact of the basisphenoid with the vomer and/or palatines present. Modifed from JY1 (ch 44) & STF (ch 65) (Pterygoid G). Remarks: two additional states were added to differentiate the length of the contact in relationship to the basisphenoid midline length. Ordered.

68. Pterygoids, pterygoid contribution to foramen palatinum posterius: 0 = present; 1 = absent. JY1 (ch 45) & STF (ch 66) (Pterygoid H).

69. Pterygoids, vertical flange on processus pterygoideus externus: 0 = absent; 1 = present. Zhou et al. (2014) & JY1 (ch 67) (Pterygoid I).

70. Pterygoids, contact with the exoccipital: 0 = absent; 1 = present. STF (ch 68, Pterygoid J).

71. Pterygoids, fossa podocnemidoidea or cavum pterygoidei: 0 = absent; 1 = present. Lapparent de Broin (2000); STF (ch 69, Pterygoid K).

72. Pterygoids, processus pterygoideus externus: 0 = large, forming an extensive lateral wing; 1 = reduced, forming an acute tip; 2 = extremely reduced due to the posterolateral projection of the pterygoid; 3 = absent. Modifed from PH (ch 11); HY2, KL, & BR (ch 22); STF (ch 70, Pterygoid L). Ordered.

73. Pterygoids, level of the position of the pterygoid respect to basisphenoid: 0 = both bones are at the same level on ventral surface; 1 = two different levels, creating a step between the two bones. Reworded from STF (ch 71, Pterygoid M).

74. Pterygoids, medial ridge: 0 = incipient to absent; 1 = present, ridge spans nearly the full length of the pteygoids, sometimes reaching the most posterior portion of the vomer. The medial ridge is produced by the extremely concave posterolateral portions of both pterygoids. Reworded from PH (ch 14); HY2, KL, & BR (ch 23).

75. Pterygoids, extending laterally almost reaching the mandibular condyle facet: 0 = absent; 1 = present, the pterygoid contacts the medial edge of the mandibular condyle when is seem in ventral view; 2 = present, the pterygoids extends not only laterally to reach the outline of the mandibular condyle facet, but also posteriorly far from the level of the condyles. Reworded from HY2, KL, & BR (ch 24). Ordered.

76. Supraoccipitals, crista supraoccipitalis: 0 = poorly developed; 1 = protruding signifcantly posterior to the foramen magnum. JY1 (ch 46) & STF (ch 72) (Supraoccipital A); HY2, KL, & BR (ch 28).

77. Supraoccipitals, large supraoccipital exposure on dorsal skull roof: 0 = absent; 1 = present. JY2 (ch 49) & STF (ch 73) (Supraoccipital B).

78. Supraoccipitals, horizontal crest in the crista supraoccipitalis: 0 = absent or poorly developed anteriorly; 1 = present, along the entire crista supraoccipitalis. STF (ch 74, Supraoccipital C).

79. Exoccipitals, medial contact of exoccipitals dorsal to foramen magnum: 0 = absent; 1 = present. JY1 (ch 48) & STF (ch 75) (Exoccipital A).

80. Basioccipital, morphology of the anteriormost part of the basioccipital: 0 = with two or one ventral tubercle; 1 = tubercle absent. ST (ch 52); STF (ch 76, Basioccipital A).

81. Basioccipital, deep C-shaped concavity between basioccipital tubera: 0 = absent; 1 = present. STF (ch 77, Basioccipital B).

82. Prootic, dorsal exposure: 0 = large; 1 = very reduce or absent. STF (ch 78, Prootic A).

83. Opisthotics, wide transverse occipital plane with depression for the nuchal musculature: 0 = absent; 1 = present. ST (ch 54); STF (ch 80, Opisthotic B).

84. Opisthotics, ventral ridge on opisthotic: 0 = absent; 1 = present, with an incipient enclosed middle ear region; 2 = present, but modifed with an enclosed middle ear region. ST (ch 55); STF (ch 81, Opisthotic C).

85. Opisthotics, procesus interfenestralis: 0 = present, but not reaching the floor of cavum acustico-jugulare; 1 = present, reaching the floor of the cavum acusticojugulare but small; 2 = present, reaching the floor of the cavum acustico-jugulare but robust. ST (ch 56); STF (ch 82, Opisthotic D). Ordered.

86. Basisphenoid, rostrum basisphenoidale: 0 = flat; 1 = rod-like, thick and rounded. JY2 (ch 56) & STF (ch 83) (Basisphenoid A); PH (ch 15); HY2, KL, & BR (ch 34).

87. Basisphenoid, paired pits on ventral surface of basisphenoid: 0 = absent; 1 = present. JY2 (ch 57) & STF (ch 84) (Basisphenoid B).

88. Basiphenoid, ventral surface: 0= flat to slightly convex, with posterior margin straight or slightly concave; 1=V-shaped crest, with posterior margin forming the basipterygoid

process projected posterolaterally. Character combined from HY2, KL, & BR (ch 31 and 32), STF (ch 85, Basisphenoid C).

89. Basiphenoid, rough surface between basisphenoid and basioccipital: 0 = absent; 1 = present. STF (ch 87, Basisphenoid E).

90. Basiphenoid, dorsum sellae: 0 = low; 1 = high. PH (ch 16); HY2, Kl, & BR (ch 33). Remarks: coded for few fossils because: poorly described specimens, lack of fgures detailing this feature, or obscured by rock matrix.

91. Basisphenoid, foramen caroticum laterale larger than foramen anterius canalis carotici interni: 0 = absent; 1 = present. PH (ch 5); HY2, KL, & BR (ch 37). Remarks: coded for few fossils because: poorly described specimens, lack of fgures detailing this feature, or obscured by rock matrix.

92. Basiphenoid, foramen anterius canalis carotici interni visible in dorsalanterior view of basisphenoid: 0 = widely separated; 1 = close together. HY2, KL, & BR (ch 29). Remarks: coded for few fossils because: poorly described specimens, lack of fgures detailing this feature, or obscured by rock matrix.

93. Hyomandibular, path of hyomandibular branch of the facial nerve: 0 = hyomandibular nerve passes through cranioquadrate space parallel to vena capitis lateralis; 1 = hyomandibular nerve runs independent from vena capitis lateralis. JY1 (ch 52) & STF (ch 95) (Hyomandibular Nerve A).

94. Stapedial Artery, size of foramen stapedio-temporale: 0 = relatively large (the size of a large blood foramina, $\geq$5 mm diameter); 1 = signifcantly reduced in size (the size of a nerve foramina, $\leq$3 mm diameter); 2 = absent. JY1 (ch 54) & STF (ch 90) (Stapedial Artery B). Remarks: we do not agree with the rationale of Anquetin (2012) and retain this as a multistate character. Ordered.

95. Stapedial Artery, foramen stapedio-temporale location in the otic chamber: 0 = on dorsal part and pointing dorsally; 1 = on the anterior wall of the otic region, pointing anteriorly. Reworded from STF (ch 91, Stapedial Artery C).

96. Recessus scalae tympani: 0 = almost nonexistent, not surrounded by bone; 1 = well developed. STF (ch 92, Recessus scalae tympani A).

97. Foramen jugulare posterius, relationship with the fenestra postotica: 0 = separate from fenestra postotica; 1 = coalescent with fenestra postotica. STF (ch 93, Foramen jugulare posterius A). Remarks: coded for few fossils because: poorly described specimens, lack of fgures detailing this feature, or obscured by rock matrix.

98. Foramen nervi hypoglossi (XII), ventral covering: 0 = exposed in ventral view; 1 = covered in ventral view by an extension of the pterygoid and the basioccipital; 2 = covered in ventral view by an extension of the basioccipital. STF (ch 95, Foramen nervi hypoglossi A).

99. Internal Carotid Artery, splitting of the internal carotid artery and the cerebral and palatine arteries: 0 = not embedded in braincase bone elements, the cerebral artery enters at the foramen posterius canalis carotici cerebralis (known previously as the foramen caroticum basisphenoidale) in the basisphenoid; 1 = partially embedded, the internal carotid artery enters in the braincase elements through the foramen posterius canalis carotici interni, running along the pterygoid canal, and then splitting into the cerebral and palatine arteries at the fenestra caroticus; 2 = fully embedded, the internal carotid artery enters in the braincase elements through the foramen posterius canalis carotici interni, and split inside the braincase, lack of a ventral exposed fenestra caroticus. Combined character from HY2, KL, & BR (ch 30 and 36). Ordered.

100. Internal Carotid Artery, foramen posterius canalis carotici interny: 0 = absent; 1 = formed by pterygoid; 2 = formed by pterygoid and basisphenoid halfway along the basisphenoid-pterygoid suture; 3 = formed by prootic, prootic and basisphenoid, or prootic and pterygoid; 4 = formed by basisphenoid only. Reworded from JY1 (ch 56, Canalis Caroticum A); STF (ch 100, Canalis Caroticum G).

101. Palatine Artery, entering in the skull: 0 = through the interpterygoid vacuity or intrapterygoid slit; 1 = through the foramen posterius carotici palatinum between basisphenoid and pterygoid. Reworded from STF (ch 99, Canalis Caroticum F). Remarks: according to the

defnition of the foramina in Rabi et al. (2013), the entry of the palatine artery is through the foramen posterius carotici palatinum, known before as the foramen caroticum laterale.

102. Fenestra Perilymphatica: 0 = large; 1 = reduced in size to that of a small foramen. JY1 (ch 57) & STF (ch 101) (Fenestra Perilymphatica A). Remarks: coded for few fossils because: poorly described specimens, lack of fgures detailing this feature, or obscured by rock matrix.

103. Cranial scutes, scute D meeting in midline: 0 = absent; 1 = present. STF (ch 103) (Cranial Scute B).

104. Cranial scutes, scute X much smaller than scute D: 0 = absent; 1 = present. STF (ch 104) (Cranial Scute C).

105. Cranial scutes, scute X partially separates scutes G: 0 = absent; 1 = present. STF (ch 105) (Cranial Scute D).

106. Cranial scutes, scutes A, B, and C forming a continus posterolateral shelf: 0 = absent; 1 = present. STF (ch 106) (Cranial Scute E).

107. Cranial scutes, scute F: 0 = formed by several scutes; 1 = formed by a single scute. STF (ch 116) (Cranial Scute O).

108. Cranial scutes, scute J: 0 = formed by several scutes; 1 = formed by a single scute. STF (ch 117) (Cranial Scute P).

109. Dentary, medial contact of dentaries: 0 = fused; 1 = open suture. JY1 (ch 58) & STF (ch 120) (Dentary A).

110. Dentary, width triturating surface vs jaw length: 0 = narrow triturating surface, symphysis less than 1/3 of jaw length; 1 = broad triturating surface, symphysis $\geq$1/3 jaw length. Reworded from HY2, KL, & BR (ch 39).

111. Dentary, symphyseal ridge: 0 = absent, flat triturating surface; 1 = present, but not visible in lateral view, flat to slightly convex triturating surface; 2 = present and greatly developed,

visible in lateral view, ridge along entire length of symphysis. Reworded from HY2 (ch 41 & 42); PH (ch 6), & KL (ch 41). Ordered.

112. Dentary, lingual (tomial) ridge: 0 = prominent; 1 = weak or absent. HY2 (ch 43), PH (ch 7), KL & BR (ch 42).

113. Dentary-Surangular arrangement: 0 = lack of a posterior expansion of dentary and anterior projection of surangular; 1 = posterior expansion of dentary present almost reaching the articular surface, covering the dorsal half of the surangular in lateral view, surangular with anterior projection. HY2 (ch 44), PH (ch 8). Reworded from KL (ch 43).

114. Splenial: 0 = present; 1 = absent. JY1 (ch 59, Splenial A); HY2 (ch 45); KL & BR (ch 44).

115. Carapace, carapacial scutes: 0 = present; 1 = reduced not fully covering the carapace; 2 = absent. Reworded from JY1 (ch 60) & STF (ch 121) (Carapace A) and HY2, KL & BR (ch 80). Joyce's (2007) original wording for the character is somewhat confusing, as it is unclear how carapacial scutes might be "partially present". The original intention of this character was to capture the presence of carapacial scutes in some turtles that only cover part of the shell. This condition is found in *Mesodermochelys undulatus* and *Pseudanosteira pulchra*. Scutes are also found in juvenile individuals of *Carettochelys insculpta* (Zangerl 1959). We do not follow the reduction of this character to two character states, as proposed by Anquetin (2012). Ordered.

116. Carapace, three parallel lines of keels: 0 = absent; 1 = present, but only poorly developed; 2 = present and pronounced; 3 = present, but only with a medial line of keels on neurals, absence of keels on costals. JY1 (ch 61) & STF (ch 122) (Carapace B) and HY2, KL, & BR (ch 84). We do not follow the proposed reduction of this character to two character states (Anquetin 2012, character 88). Remarks: Sterli and de la Fuente (2013) coded *Araripemys barretoi* as (2), but examination of the holotype indicates that is poorly developed, changed here to (1). Also *Platychelys oberndorferi* is changed here from (0) to (1). A third state was added to include forms with a single medial line of keels as in protostegids and some cheloniids.

117. Shell, sculpturing of dorsal surface (carapace) and ventral surface (plastron): 0 = absent, smooth to slightly rugose; 1 = present, development of striations, vermiculations, striations, or pitting. Modifed from STF (ch 124) (Carapace D). Remarks: *Proganochelys quenstedti* is coded here as (0&1) with marked striations in the posterior portion of the carapace.

118. Shell, pattern of sculpturing of the dorsal surface (carapace) and ventral surface (plastron): 0 = parallel to radial striations; 1 = vermiculation; 2 = highly dense pattern of pitting combined with striations; 3 = dichotomic striations; 4 = spread pitting without marked striation pattern; 5 = granules (positive relief). Modifed from STF (ch 125) (Carapace E).

119. Carapacial Sutures: 0 = carapacial elements fnely sutured or the contact is smooth; 1 = carapacial sutures strongly serrated in adult stage. Character from Zhou et al. (2014) (ch 244).

120. Nuchal, articulation of nuchal with neural spine of eighth cervical vertebra: 0 = cervical articulates with nuchal along a blunt facet; 1 = articulation absent; 2 = cervical articulates with nuchal along a raised pedestal. JY1 (ch 62) & STF (ch 126) (Nuchal A). We do not follow Anquetin (2012) and retain this character as a single multistate character.

121. Nuchal, elongate costiform process: 0 = absent; 1 = present, crosses peripheral 1; 2 = present, well developed reaches peripherals 2 or 3. Modifed from JY1 (ch 63) & STF (ch 127) (Nuchal B). Remarks: we adjust the scoring of *Baptemys wyomingensis* and *Dermatemys mawii* to 1 (Knauss et al. 2011). State (1) was splitted in state (1) and (2). Ordered.

122. Nuchal, length versus width: 0 = wider than long; 1 = longer than wide or as long as wide. de la Fuente (2003) & STF (ch 128) (Nuchal C).

123. Nuchal, posteriomedial fontanelles: 0 = absent; 1 = present. HY2, KL, & BR (ch 81) & PH (ch 30). Remarks: Bardet et al. (2013) coded as present for *Erquelinnesia gosseleti* (Zangerl 1971).

124. Neurals, neural formula $6>4<6<6<6<6$: 0 = absent; 1 = present. JY1 (ch 64) & STF (ch 129) (Neural A).

125. Neurals, shape of neurals: 0 = very irregular in shape, wider than long or squared; 1 = regular, often perfectly hexagonal or pentagonal, longer than wide. STF (ch 130) (Neural B) & HY2, KL & BR (ch 86).

126. Neurals, number of neurals: 0 = ten or more; 1 = nine or less; 3 = CO all neurals lost even in ventral view. Modified character from HY2, KL & BR (ch 85 & ch 87) and PH (ch 33). State character (3) reworded. Remarks: a combined character from HY2 (ch 85 & ch 87) is proposed here that covers all the possible variations in the number and reduction of neurals.

127. Peripheral Gutter: 0 = peripheral gutter absent of only anteriorly developed; 1 = peripheral gutter extensively developed along anterior and bridge peripherals. Character from Zhou et al. (2014) (ch 246).

128. Peripherals, number of peripherals: 0 = more than 11 pairs of peripherals present; 1 = 11 pairs of peripherals present; 2 = 10 pairs of peripherals present; 3 = less than 10 pairs of peripherals present. JY1 (ch 65) & STF (ch 131) (Peripheral A). Remarks: we do not follow Anquetin (2012) and retain this character as a single multistate character. Ordered.

129. Peripherals, anterior peripherals incised by musk ducts: 0 = absent; 1 = present. JY1 (ch 66) & STF (132) (Peripheral B).

130. Costals, medial contact of the frst pair of costals: 0 = absent; 1 = present. Reworded from JY1 (ch 67) & STF (ch 133) (Costal A).

131. Costals, medial contact of posterior costals: 0 = absent; 1 = medial contact of up to three posterior costals present; 2 = medial contact of all costals present. Modifed from JY1 (ch 68) & STF (ch 134) (Costal B). Remarks: we do not follow Anquetin (2012) and retain this character as a single multistate character. However, we follow Anquetin (2012) by adjusting the scoring for *Mesodermochelys undulatus*. Ordered.

132. Costals, distal rib end and lateral ossifcation of the costal: 0 = costals fully ossifed laterally with strong sutural contact with peripherals, lack of dorsal exposure of distal end of costal ribs; 1 = costals fully ossifed laterally with strong sutural contact with peripherals, distal

end of costal ribs exposed on dorsal surface and surrounded by the peripheral; 2 = costals lack lateral ossifcation, allowing the dorsal exposure of the distal end of ribs and the development of fontanelles only at the most anterior and posterior costals; 3 = costals with extreme lost of lateral ossifcation, allowing the dorsal exposure of the distal end of ribs, in almost all series of costals. Remarks: character reworded from STF (ch 135) (Costal C) and the combination of characters (ch 243) (costal rib) and (ch 247) (costal rib distal end) from Zhou et al. (2014).

133. Rib free peripherals: 0 = absent; 1 = present, only anterior and posterior to ribs; 2 = present, between sixth and seventh ribs; 3 = present, between seventh and eighth ribs. Reworded from PH (ch 29).

134. Costals, alternative short and long ends in the lateral part of costals: 0 = absent; 1 = present. STF (ch 136) (Costal D).

135. Costals, costal 9: 0 = present; 1 = absent. Reworded from HY2, KL & BR (ch 90).

136. Costals, shape of Costal 3: 0 = tapering towards the lateral side of the shell or with parallel anterior and posterior borders; 1 = broadens towards the lateral side of the shell. Character from Zhou et al. (2014) (ch 242).

137. Suprapygals, number of suprapygals: 0 = one; 1 = two; 2 = more than two; 3 = absent. Hirayama et al. (2000) and STF (ch 137) (Suprapygal A). Ordered.

138. Suprapygals, size between suprapygal 1 and 2: 0 = suprapygal 1 smaller than suprapygal 2; 1 = suprapygal 1 larger. Reworded from KL (ch 88). Remarks: turtles with only one suprapygal or suprapygals absent are coding as (-).

139. Cervical scute: 0 = more than one cervical scute present; 1 = one cervical scute present; 2 = cervical scutes absent, carapacial scutes otherwise present. JY1 (ch 70) & STF (ch 138) (Cervical A). Remarks: we do not follow Anquetin (2012) and retain the original multistate arrangement for this character.

140. Pygal, posterior notch: 0 = present; 1 = absent. Modifed from Lapparent de Broin and Murelaga (1999) & PH (ch 35).

141. Supramarginals: 0 = complete row present, fully separating marginals from pleurals; 1 = partial row present, incompletely separating marginals from pleurals; 2 = absent. JY1 (ch 71) & STF (ch 139) (Supramarginal A). Remarks: we do not follow Anquetin (2012) and retain the original multistate arrangement for this character. We furthermore retain the scoring of *Platychelys oberndorferi* as 1, as this taxon clearly exhibits supramarginals (Bräm 1965). The Munich specimens are not informative in this regard, as the lateral portions of the shell are not preserved. In addition to its supramarginals, *P. oberndorferi* also possesses supernumerary pleural scales (Joyce 2003). Ordered.

142. Vertebrals, shape of the verterbrals: 0 = vertebrals 2 to 4 signifcantly broader than pleurals; 1 = vertebrals 2 to 4 as narrow as, or narrower than, pleurals. JY1 (ch 73) & STF (ch 141) (Verterbal B).

143. Vertebrals, position of vertebral 3-4 sulcus in taxa with fve vertebrals: 0 = sulcus positioned on neural 6; 1 = sulcus positioned on neural 5. JY1 (ch 74) & STF (142) (Vertebral C).

144. Vertebrals, vertebral 3-4 sulcus with a wide posteriorly oriented medial embayment: 0 = absent; 1 = present. AN (ch 108).

145. Vertebrals, verebral 1: 0 = vertebral 1 does not enter anterior margin of carapace; 1 = enters anterior margin. Character from Zhou et al. (2014) (ch 245).

146. Marginals, marginal scutes overlap onto costals: 0 = absent, marginals restricted to peripherals; 1 = present. Meylan and Gaffney (1989) & STF (ch 143) (Marginal A).

147. Pleurals, at least one pair of additional pleural scutes located laterally of vertebral scute 1, with anterior contact with cervical scute: 0 = absent; 1 = present. Modifed from PH (ch 32). Plastron

148. Plastron, connection between carapace and plastron: 0 = osseous; 1 = ligamentous. JY1 (ch 75) & STF (ch 144) (Plastron A). Remarks: we adjust Anquetin (2012) scoring of *Odontochelys semitestacea* to 1, as it is apparent that a turtle lacking peripherals can only have a ligamentous bridge.

149. Plastron, central plastral fontanelle: 0 = absent; 1 = present. Modifed from JY1 (ch 76) & STF (ch 145) (Plastron B) and HY2, KL & BR (ch 97). Remarks: see recommendation in Character 143 about levels of ossifcation and ontogeny.

150. Plastron, posterior plastral fontanelle, posterior plastral fontanelle between the xiphiplastra and/or the hypoplastra: 0 = absent in adult stage; 1 = retained in adult stage. Character from Zhou et al. (2014) (ch 239).

151. Plastron, plastral kinesis: 0 = absent, scutes sulci and bony sutures do not overlap; 1 = present, scutes sulci coincide with epiplastral-hyoplastral contact. JY1 (ch 77, Plastron C).

152. Plastron, plastral kinesis: 0 = between hyoplastron and hypoplastron; 1 = between hyoplastron and epiplastronentoplastron. STF (ch 148) (Plastral Kinesis B).

153. Plastron, hyo-hypoplastra contact and shape: 0 = deep U or V-shaped axillar and inguinal notches, contact between hyo-hyoplastra absent or reduced due to the presence of mesoplastra or a central fotanelle; 1 = deep axillar and inguinal notches, reduced contact between both elements due to the existence of central and lateral fontanelles; 2 = deep axillar and inguinal notches, extensive contact between hyo-hyoplastra (even for those taxa with plastral kinesis); 3 = a very narrow to absent contact between each other, star-shaped with extremely serrate medial edges, very shallow axillar and inguinal notches, and long lateral edges; 4 = extreme loss of ossifcation of hyo-hypoplastra, lack of contact between each other. Combined from HY2, KL & BR (ch 96) and PH (ch 28). Remarks: two states were added to cover all possible variations in the contact between hyo-hypoplastra and the shape of both elements related to the presence of mesoplastra or fontanelles.

154. Entoplastron: 0 = present; 1 = absent. STF (ch 153) (Entoplastron E).

155. Entoplastron, anterior entoplastral process: 0 = present, medial contact of epiplastra absent; 1 = absent, medial contact of epiplastra present. JY1 (ch 78) & STF (ch 149) (Entoplastron A).

156. Entoplastron, size of the posterior entoplastral process: 0 = posterior process long, reaching as far posteriorly as the mesoplastra; 1 = posterior process reduced in length. JY1 (ch 79) & STF (150) (Entoplastron B).

157. Entoplastron, distinct posterolateral process: 0 = present; 1 = absent. JY1 (ch 80) & STF (ch 151) (Entoplastron C).

158. Entoplastron, shape of the entoplastron in ventral view: 0 = dagger-shaped; 1 = massive diamond-shaped; 2 = T-shaped, longer than wide; 3 = T-shaped, wider than long, forming broad lateral wings; 4 = strap like and V-shaped. Combined from JY1 (ch 81) & STF (ch 152) (Entoplastron D); AN (ch 116); and HY2, KL & BR (ch 101).

159. Entoplastron, suture with hyoplastra: 0 = tightly sutured; 1 = lightly sutured to almost absent contact between both. Modifed from HY2, KL & BR (ch 99), and STF (ch 154) (Entoplastron F).

160. Epiplastra, shape and contact of epiplastra: 0 = epiplastra squarish in shape, lack a contact between each other due to the narrow participation of the entoplastron in the anterior plastral lobe edge; 1 = epiplastra elongate in shape, with medial contact located anterior to the entoplastron; 2 = epiplastra squarish in shape lack of medial contact due to the extensive anterior and lateral projections of the entoplastron. Modifed from JY1 (ch 83, Epiplastron A). Remarks: A third state is added to describe variations in the participation of the entoplastron to the anterior plastral lobe edge.

161. Epiplastra, very thick anterior lip in dorsal view: 0 = present; 1 = absent. Hirayama et al. (2000) and STF (ch 156) (Epiplastron B).

162. Hyoplastra, contacts of axillary buttresses: 0 = absent to slightly contacting peripherals only; 1 = peripherals and costal 1. Modifed from JY1 (ch 84) & STF (ch 157) (Hyoplastron A) and HY2, KL & BR (ch 92).

163. Hyoplastra, termination of axillary buttresses: 0 = terminates on peripheral 1 or 2; 1 = terminates on peripheral 3; 2 = terminates on peripheral 4 or 5 level; 3 = ossifed axillary buttresses absent. Reworded from Hutchison (1991) and STF (ch 159) (Hyoplastron B).

164. Mesoplastron: 0 = two present; 1 = one present; 2 = absent. Modifed from JY1 (ch 85) & STF (ch 160) (Mesoplastron A) and AN (ch 120 and ch 121). Remarks: we follow Sterli (2008) and Anquetin (2012) by splitting character 85 of Joyce (2007), but instead of three new characters, we only create two, one of which is multistate. Ordered.

165. Mesoplastron, medial contact of mesoplastra: 0 = present, or virtually present when a central plastral fontanelle is present, absence of contact between hyoplastron and hypoplastron; 1 = absent, partial contact between hyoplastron and hypoplastron present. Modified fromJY1 (ch 85) & STF (ch 160) (Mesoplastron A) and AN (ch 122). Remarks: we use the reworded character of Anquetin (2012) and follow his scoring for this character as well.

166. Hypoplastra, contacts of inguinal buttresses: 0 = absent to slightly contacting peripherals; 1 = peripheral and costal 5; 2 = peripheral, costals 5 and 6; 3 = peripherals and costal 4. Modifed from JY1 (ch 86) & STF (ch 161) (Hypoplastron A); AN (ch 123) and HY2, KL & BR (ch 93). Remarks: a third state is added for the condition in Chelus fmbriata, having an inguinal buttress restricted to costal 4 and peripherals.

167. Hypoplastra, termination of inguinal buttresses: 0 = peripheral 8; 1 = peripheral 7; 2 = peripheral 6. Iverson (1991) and STF (ch 162) (Hypoplastron B). Ordered.

168. Xiphiplastra, distinct anal notch: 0 = absent; 1 = present. JY1 (ch 87) & STF (163) (Xiphiplastron A).

169. Xiphiplastra, shape of xiphiplastra: 0 = almost triangular to trapezoidal, with lateral straight to convex margin; 1 = rectangular elongated in shape, coupled forming together with the hypoplastron a very narrow posterior plastral lobe; 2 = narrow struts, separated by the posterior fontanelle. Reworded from JY1 (ch 88) & STF (ch 164) (Xiphiplastron B) and HY2, KL & BR (chs 102, 103 and 104). Remarks: Zhou et al. (2014) proposed a new character (Plastron lobe, ch 241) for the posterior plastral lobe, however this becomes a redundant character because the shape of the posterior plastral lobe is mostly determinate by the shape of the xiphiplastron, and we combine this character with the previously defned

character 88 of Joyce (2007). However, we split the character state 1 in two: covering the two most common shapes of the xiphiplastron related to the shape of the posterior plastral lobe.

170. Plastral scutes: 0 = present; 1 = absent. JY1 (ch 89) & STF (ch 165) (Plastral scutes A) and HY2, KL & BR (ch 57).

171. Plastral scutes, midline sulcus: 0 = straight; 1 = distinctly sinuous, at least for part of its length. AN (ch 127) & STF (ch 166) (Plastral scutes B).

172. Gular, number of gulars: 0 = one pair of scutes; 1 = only one scute. Reworded from JY1 (ch 91) & STF (ch 167) (Gular A).

173. Extragulars: 0 = present; 1 = absent. JY1 (ch 92) & STF (ch 168) (Extragular A).

174. Extagulars, medial contact: 0 = absent; 1 = present, contacting one another anterior to gular(s); 2 = present, contacting one another posterior to gular(s). JY1 (ch 93) & STF (ch 169) (Extragular B). Remarks: Even though character state 2 is only developed in *Chelodina oblonga*, we retain this character as a multistate character, contra to Anquetin (2012).

175. Extragulars, anterior plastral tuberosities: 0 = present; 1 = absent. JY1 (ch 94) & STF (ch 170) (Extragular C). Remarks: we disagree in the coding for *Chelus frimbriata* and *Otwayemys cunicularius*, both taxa lack of strong tuberosities as the one in stem-testudines, coding as (1) for both here.

176. Extragulars, restricted to epiplastra: 0 = present; 1 = absent, extragulars reach the entoplastron. Reworded from An (ch 129) & STF (ch 171) (Extragular D).

177. Intergulars: 0 = absent; 1 = present. JY1 (ch 95) & STF (ch 172) (Intergular A).

178. Humerals, number of pairs: 0 = one pair present; 1 = two pairs present, subdivided by a plastral hinge. JY1 (ch 96) & STF (ch 173) (Humeral A).

179. Humerals, humero-pectoral sulcus: 0 = restricted to hyoplastra; 1 = crossing the posterior portion of entoplastron. STF (ch 174) (Humeral B). Remarks: in extant cheloniids,

this character is polymorphic, depending of the length of the posterior process of the entoplastron. This could be also the condition for most marine forms for which this character can be coded due to poor illustrations or bad preservation of sulci.

180. Pectorals: 0 = present; 1 = absent. JY1 (ch 97) & STF (ch 175) (Pectoral A).

181. Pectorals, antero-posteriorly developed: 0 = present; 1 = absent, very short antero-posterior development. STF (ch 176) (Pectoral B).

182. Abdominals: 0 = present, in medial contact with one another; 1 = present, medial contact absent; 2 = absent. JY1 (ch 98) & STF (ch 177) (Abdominal A). Remarks: we do not follow Anquetin (2012) and retain the original multistate nature of this character. The scoring of *Emarginachelys cretacea* is amended to 1 (pers. comn. of WGJ of type material). Ordered.

183. Anals: 0 = only cover parts of the xiphiplastra; 1 = overlap anteromedially onto the hypoplastra. JY1 (ch 99) & STF (ch 178) (Anal A) and HY2, KL & BR (ch 94).

184. Inframarginals: 0 = more than two pair present, plastral scales do not contact marginals; 1 = two pair present (axillaries and inguinals), limited contact between plastral scales and marginals present; 2 = absent, unrestricted contact between plastral scales and marginals present. JY1 (ch 100, Inframarginal A) & STF (ch 179, 180, and 181) (Inframarginals, A, B and C). Remarks: we do not follow Anquetin (2012) or Sterli and de la Fuente (2013) and retain the original multistate nature of this character. We furthermore adjust the scoring of all kinosternids from 0 to 1 (Knauss et al. 2011). Ordered.

185. Cervical ribs: 0 = large cervical ribs present; 1 = cervical ribs reduced or absent. JY1 (ch 101) & STF (ch 182) (Cervical Rib A). Remarks: we follow Anquetin (2012) correction for the scoring of *Palaeochersis talampayensis*.

186. Cervicals, position of the transverse processes: 0 = middle of the centrum; 1 = anterior end of the centrum. JY1 (ch 102) & STF (ch 183) (Cervical Vertebra A).

187. Cervicals, posterior cervicals with strongly developed ventral keels: 0 = absent or slightly

developed in all vertebrae; 1 = present, more developed on posterior vertebrae. JY1 (ch 103) & STF (ch 184) (Cervical Vertebra B); HY2 (ch 48); and KL & Br (ch 47).

188. Cervicals, cervical 8 centrum significantly shorter than cervical 7: 0 = absent; 1 = present. JY1 (ch 104) & STF (ch 185) (Cervical Vertebra C) and HY2, KL & BR (ch 52).

189. Cervicals, triangular diapophyses: 0 = absent; 1 = present. Gaffney (1996) and STF (ch 186) (Cervical Vertebra D).

190. Cervicals, central articulations of cervical vertebrae: 0 = articulations not formed, cervical vertebrae amphicoelous or platycoelous; 1 = articulations formed, cervical vertebrae procoelous or opisthocoelous. JY1 (ch 105) & STF (ch 187) (Cervical Articulation A); HY2 (ch 49); and Kl & BR (ch 48). Remarks: Bardet et al. (2013) coded *Notochelone* as amphicoelous based on the descriptions from Gaffney (1981).

191. Cervicals, articulation between cervical 8 and dorsal vertebrae 1: 0 = 8 (dorsal 1; 1 = 8) dorsal 1; 2 = none, vertebrae only meet at zygapophyses. JY1 (ch 112) & STF (ch 188) (Cervical Articulation H) and RH2, KL & BR (ch 51). Remarks: we do not follow Anquetin (2012) and retain the original multistate character of Joyce (2007).

192. Cervicals, biconvex cervical vertebrae in the middle of the neck: 0 = absent; 1 = present. STF (ch 189) (Cervical Vertebra E).

193. Cervicals, biconvex cervical vertebra in the middle of the neck: 0 = cervical 2; 1 = cervical 3; 2 = cervical 4; 3 = cervical 5. JY1 (ch 106) (Cervical Articulation B, C & D); STF (ch 190) (Cervical Vertebra F).

194. Cervicals, biconcave cervical vertebrae: 0 = absent; 1 = present. STF (ch 191) (Cervical Vertebra G).

195. Cervicals, double articulation between cervical 5 and 6: 0 = absent; 1 = present. JY1 (ch 109) (Cervical Articulation E); STF (ch 192) (Cervical Vertebra I).

196. Cervicals, double articulation between cervical 6 and 7: 0 = absent; 1 = present. JY1 (ch 110) (Cervical Articulation F); STF (ch 193) (Cervical Vertebra J).

197. Cervicals, central articulation between cervical 6 and 7: 0 = cervical 6 concave (cervical 7 convex; 1 = platycoelous, cervical 6 II cervical 7. JY1 (ch 110) (Cervical Articulation F); STF (ch 194) (Cervical Vertebra K).

198. Cervicals, double articulation between cervical 7 and 8: 0 = absent; 1 = present. JY1 (ch 111) (Cervical Articulation G); STF (ch 195) (Cervical Vertebra L); PH (ch 27); and RH2, KL & BR (ch 51 and 53).

199. Cervicals, height versus length of centra and neural arch: 0 = total height of centra and neural arch longer than the anteroposterior length of the cervical centra; 1 = total height of centra and neural arch much shorter than the anteroposterior length of the cervical centra. STF (ch 196) (Cervical Vertebra H).

200. Cervicals, modifcation of neural arch on cervical 8: 0 = neural arch without modifciation of postzygapophyses; 1 = neural arch with postzygapophyses poiting anteroventrally. STF (ch 197) (Cervical Vertebra I).

201. Cervicals, postzygapophyses united in midline: 0 = absent; 1 = present. Bona and de la Fuente (2005); STF (ch 198) (Cervical Vertebra J).

202. Cervicals, ventral process on cervical 8: 0 = absent; 1 = present, well developed (as tall or taller than the height of the centrum). STF (ch 199) (Cervical Vertebra K).

203. Cervicals, shape of central articulation of cervicals 7 and 8: 0 = as high as wide; 1 = much wider than high. Reworded from HY2 (ch 47), KL & BR (ch 46).

204. Ribs, length of frst dorsal rib: 0 = long, extends full length of frst costal and may even contact peripherals distally; 1 = intermediate, in contact with welldeveloped anterior bridge buttresses; 2 = intermediate to short, extends less than halfway across frst costal. JY1 (ch 113) & STF (ch 200) (Dorsal Rib A) and HY2, KL & BR (ch 55). Remarks: although we agree with Anquetin (2012) that the anterior plastral buttress cannot be used as a fxed reference when assessing the length of the frst thoracic, our experience with this character demonstrates that all turtle clearly fall into the three classes developed as

character states herein. We therefore maintain the character of Joyce (2007) as originally developed. Ordered.

205. Ribs, contact of dorsal ribs 9 and 10 with costals: 0 = present; 1 = absent. JY1 (ch 114) & STF (ch 201) (Dorsal Rib B).

206. Dorsal rib 10: 0 = long, spanning full length of costals and contacting peripherals distally; 1 = short, not spanning father distally than pelvis. JY1 (ch 115) & STF (ch 202) (Dorsal Rib C), HY2, KL & BR (ch 56). Remarks: we follow AnquetinâĂŹs (2012) adjustment in the scoring of *Santachelys gaffneyi*.

207. Dorsals, anterior articulation of the frst dorsal centrum: 0 = faces at most slightly anteroventrally; 1 = faces strongly anteroventrally. JY1 (ch 116) & STF (ch 203) (Dorsal Vertebra A) and HY2, KL & BR (ch 54).

208. Caudals, tail club: 0 = present; 1 = absent. JY1 (ch 118) & STF (ch 204) (Caudal A).

209. Caudals, caudal centra: 0 = all centra amphicoelous; 1 = all centra more or less pronounced procoelous; 2 = all centra more or less pronounced opisthocoelous; 3 = anterior few centra procoelous, posterior centra predominantly opisthocoelous. JY1 (ch 119) & STF (ch 205) (Caudal B) and HY2, KL & BR (ch 58 and 59). Remarks: we do not follow Anquetin (2012) and retain the original multistate character of Joyce (2007).

210. Caudals, anterior caudal centra: 0 = amphicoelous; 1 = procoelous or platycoelous; 2 = opisthocoelous. STF (ch 206) (Caudal C).

211. Caudals, posterior caudal centra: 0 = amphicoelous; 1 = procoelous or platycoelous: 2 = opisthocoelous. STF (ch 207) (Caudal D).

212. Caudals, chevrons: 0 = present on nearly all caudal vertebrae; 1 = absent, or only poorly developed, along the posterior caudal vertebrae. JY1 (ch 117) & STF (ch 207) (Chevron A) and HY2, KL & BR (ch 57).

213. Caudals, tail ring: 0 = absent; 1 = present. STF (ch 208) (Tail Ring A).

214. Scapula, anterodorsal ridge of acromion: 0 = present; 1 = absent. New character. Remarks: the acromion ridge of basal turtles such as *Proganochelys quenstedti* is triradiate in cross section due to the developed of three ridges. The anterodorsal ridge (sensu Gaffney 1990) runs from the acromion to the dorsal process of the scapula. The ventral ridge (sensu Gaffney 1990; acromion ridge sensu Joyce 2007) runs from the acromion to the glenoid. The horizontal ridge (sensu Gaffney 1990) spans between the acromion and the coracoid and may contain the coracoid foramen. These three ridges are apparent lost in steps independently from one another and we therefore reorganize characters 122 and 124 of Joyce (2007) into three characters, one of which of multistate. These character partially contain the morphologies discussed by Sterli (2007, character 75) and Anquetin (2012, character 165).

215. Scapula, ventral ridge of acromion: 0 = present; 1 = absent developed proximally near glenoid. Reworded from JY1 (ch 122, Scapula B).

216. Scapula, horizontal ridge of acromion: 0 = well-developed, coracoid foramen present; 1 = reduced, only developed along distal portion of acromion. Modifed from JY1 (ch 124, Coracoid A). Ordered.

217. Scapula, glenoid neck on scapula: 0 = absent; 1 = present. JY1 (ch 123, Scapula C).

218. Scapula, lamina between the dorsal process of the scapula and the acromion: 0 = well developed; 1 = reduced; 2 = absent. STF (ch 215) (Scapula A). Ordered.

219. Scapula, internal angle between acromion process and scapular process ≥110°: 0 = absent; 1 = present. Reworded from PH (ch 18) and HY2, KL & BR (ch 61).

220. Coracoid, coracoid vs humerus length: 0 = shorter than humerus; 1 = at least as long as humerus. PH (ch 26) and HY2, KL & BR (ch 60).

221. Coracoid, foramen: 0 = present; 1 = absent. Gaffney et al. (2007) and Joyce et al. (2013) (ch 82).

222. Cleithrum: 0 = present and in contact with the carapace; 1 = present, osseous contact with carapace absent; 2 = absent. JY1 (ch 120) & STF (ch 214) (Cleithrum A). Ordered.

223. Pelvis, pelvis-shell attachment: 0 = pelvis-shell attachment by ligaments; 1 = pelvis attached by strong sutural contact of the ischium and pubis with the plastron, and illium with the carapace. ST (ch 138); JY1 (ch 134) & STF (ch 221) (Pelvis A). Remarks: States 1 and 2 of STF combined in one, considering that one of them is only apomorphic for *Palaeochersis*.

224. Pelvis, thyroid fenestra: 0 = coalescent; 1 = two separated fenestra completely or partially separated. STF (ch 222) (Pelvis B).

225. Ilium, elongated iliac neck: 0 = absent; 1 = present. JY1 (ch 126) & STF (ch 225) (Ilium A).

226. Ilium, iliac scar: 0 = extends from costals onto the peripherals and pygal; 1 = positioned on costals only. JY1 (ch 127) & STF (ch 226) (Ilium B).

227. Ilium, shape of the ilium articular site on the visceral surface of the carapace: 0 = narrow and pointed posteriorly; 1 = oval. JY1 (ch 128) & STF (ch 227) (Ilium C).

228. Ilium, posterior notch in acetabulum: 0 = absent; 1 = present. JY1 (ch 129) & STF (ch 228) (Ilium D).

229. Ilium, thelial process: 0 = absent; 1 = present. Meylan (1987); STF (ch 229) (Ilium E).

230. Pubis, lateral process: 0 = small, poorly developed, columnar; 1 = well developed and flat. STF (ch 223) (Pubis A); HY2, KL & BR (ch 62).

231. Pubis, epipubis process: 0 = osseus or calcifed; 1 = cartilaginous or absent. STF (ch 224) (Pubis B).

232. Ischium, ischial contacts with plastron: 0 = contact via a large central tubercle; 1 = contact via two separate ischial processes. JY1 (ch 130, Ischium A).

233. Ischium, lateral process of ischium or metischial process: 0 = absent; 1 = present. PH (ch 19); HY2, KL & BR (ch 64); STF (ch 230) (Ischium A).

234. Hypoischium: 0 = present; 1 = absent. JY1 (ch 131, Hypoischium A).

235. Humerus, ectepicondylar foramen: 0 = in a channel; 1 = only a groove. Meylan (1987) & STF (ch 216) (Humerus A).

236. Humerus, proximal articular surface of humerus: 0 = with shoulder on preaxial side, upturned; 1 = without shoulder, not upturned. Gaffney (1990); HY2, KL & BR (ch 68); STF (ch 217) (Humerus B).

237. Humerus, lateral process of humerus: 0 = abuts caput humeri; 1 = slightly separated from caput humeri; 2 = located distal to caput humeri but along proximal end of shaf; 3 = located at middle of humeral shaf. HY2, KL & BR (ch 67); STF (ch 218) (Humerus C). Ordered.

238. Humerus, lateral process of humerus: 0 = visible in dorsal view: 1 = not visible in dorsal view. STF (ch 219) (Humerus D).

239. Humerus, lateral process shape: 0 = rounded to slightly squared; 1 = V-shaped or triangular. Reworded from PH (ch 25) and HY2, KL & BR (ch 70).

240. Humerus, expansion of lateral process: 0 = limited to anterior surface of shaf; 1 = expands onto ventral surface. Reworded from HY2, KL & BR (ch 71).

241. Humerus, medial concavity of lateral process: 0 = absent; 1 = present. HY2, KL & BR (ch 72).

242. Humerus, prominent anterior projection of lateral process: 0 = absent; 1 = present. HY2, KL & BR (ch 73).

243. Humerus, length of the humerus versus the width of the proximal end: 0 = two times or less the width of the proximal end: 1 = more than two times the width of the proximal end. STF (ch 220) (Humerus E).

244. Humerus, scar for Muscle latissimus dorsi and Muscle teres major: 0 = located anterior to humeral shaf; 1 = located at middle of shaf. HY2, KL & BR (ch 69).

245. Humerus, humerus length vs femur length: 0 = shorter than femur; 1 = longer than femur. HY2, KL & BR (ch 66).

246. Ulna, contact with radius through rugosity and ridge: 0 = absent; 1 = present. HY2, KL & BR (ch 74).

247. Radius, curves towards anterior: 0 = absent; 1 = present. HY2, KL & BR (ch 75).

248. Manus, phalangeal formula of the manus: 0 = most digits with two shortenened phalanges: 1 = most digits with three elongated phalanges. JY1 (ch 132) & STF (ch 232) (Manus A).

249. Manus, paddles: 0 = absent, moveable articulations of all digits; 1 = 'half-paddle' digits 3 and 5 modifed into paddle with rigid articulations. But digits 1 and 2 immoveable; 2 = elongate paddles present, digits 1 and 2 modifed into paddle with rigid articulations, and very flat carpal and tarsal elements. Combined from JY1 (ch 133, Manus B) and HY2, KL & BR (chs 76, 77 and 78). Remarks: we do not follow Anquetin (2012) and retain this as a multistate character. Ordered.

250. Manus, flippers: 0 = absent; 1 = short flippers present; 2 = elongate flippers present. JY1 (ch 134, Manus C). Remarks: we do not share Anquetin (2012) reservation in regards to this character and leave it in the matrix. Ordered.

251. Ulnare, size of the ulnare vs the intermedium: 0 = smaller than intermedium: 1 = nearly as large as intermedium; 2 = much larger than intermedium. Remarks: character defned by Tong et al. (2006), but first time included in a phylogenetic analysis. New character. Ordered.

252. Pes, number of digits: 0 = five; 1 = four. STF (ch 237) (Pes C).

253. Manus and Pes, flattening of carpals and tarsal elements: 0 = absent; 1 = present. HY2, KL & BR (ch 76); STF (ch 238) (Manus and Pes A).

254. Manus and Pes, hyperphalangy manus digits 4 and 5, pes digit 4: 0 = absent; 1 = present. Meylan (1987) & STF (ch 239) (Manus and Pes B).

255. Femur, femoral trochanters: 0 = distinct, and separated from one another; 1 = fossa obliterated, space between trochanters not concave, but notch present; 2 = fossa obliterated,

trochanters connected by bony ridge without a notch. Character combined from HY2, KL & BR (ch 79) and PH (ch 20 and ch 21). Ordered.

256. Tibia, tibial pit for pubotibialis and flexor tibialis internus muscles: 0 = absent; 1 = present. PH (ch 22).

257. Shape of frontal: 0 = frontal lacks anterior processes; 1 = frontal possesses anterior process and the lateral process is anteroposteriorly short (less than 1/3rd of frontal length); 2 = frontal possesses anterior process and the lateral process is anteroposteriorly long (equal or larger than half of frontal length). Note: **1** = *Rhinochelys pulchriceps, Rhinochelys amaberti, Rhinochelys nammourensis*; **2** = *Rhinochelys* morphotype '*elegans*', *Rhinochelys* morphotype '*cantabrigiensis*', *Rhinochelys* morphotype '*jessoni*'.

258. Shape of the naso-frontal region: - (inapplicable) = taxa where nasal bone is absent; 0 = nasal is as high as wide (with a ventral constriction) and straight dorsal margin; 1 = nasal is as long as wide and forms a small medial process separating the frontals medially; 2 = nasal is long and narrow (nasal-frontal suture is reduced), and forms a wide expansion that entirely forms the dorsal border of the nasal cavity (thus excluding the prefrontal from the nasal cavity); 3 = nasal is wider than long; 4 = nasal extends laterally beyond the anterior edge of the frontal. Note: **0** = *Rhinochelys pulchriceps*, *Rhinochelys* morphotype '*cantabrigiensis*', *Rhinochelys nammourensis*; **1** = *Rhinochelys* morphotype '*elegans*'; **2/3** = *Rhinochelys amaberti*; **2** = specimens IRSNB GS63 & GS67.

259. Posterodorsal extension of the maxilla in relation to the nasal cavity: 0 = the maxilla extends, laterally, beyond the nasal cavity; 1 = the maxilla does not extends beyond the posterior border of the nasal cavity. Note: **0** = *Rhinochelys* morphotype '*cantabrigiensis*', *Rhinochelys amaberti, Rhinochelys nammourensis, Rhinochelys pulchriceps*; **1** = *Rhinochelys* morphotype '*elegans*', *Rhinochelys* morphotype '*jessoni*', specimens IRSNB GS63 & GS67.

260. Labial edge of the maxilla in lateral view: 0 = the labial edge of the maxilla is relatively flat; 1 = the ventral border/labial edge of the maxilla is raised anteriorly (before the

premaxilla-maxilla suture). Note: **0** = *Rhinochelys* morphotype '*elegans*', *Rhinochelys amaberti*; **1** = *Rhinochelys pulchriceps*, *Rhinochelys* morphotype '*cantabrigiensis*', specimens IRSNB GS63 & GS67; **?** = *Rhinochelys nammourensis*.

261. Maxillary bulge above the maxillary sinusoidal sulcus: 0 = absence of a maxillary bulge; 1 = maxillary bulge is present (just above the maxillary sulcus) but is feeble; 2 = maxillary bulge (just above the maxillary sulcus) is prominent to the point of concealing the labial edge of the maxilla in dorsal view. Note : **1** = *Rhinochelys* morphotype '*cantabrigiensis*', *Rhinochelys* morphotype '*elegans*', specimens IRSNB GS63 & GS67; **2** = *Rhinochelys pulchriceps, Rhinochelys amaberti*.

262. Position of the orbits with respect to the nasal cavity in lateral view: 0 = the center of the orbit is located dorsally to the level of the center of the nasal cavity; 1 = the center of the orbit and the center of the nasal cavity are located on the same horizontal plan; 2 = the center of the orbit is located ventrally to the center of the nasal cavity. Note: **1** = all species currently assigned to *Rhinochelys*; **?** = *Rhinochelys nammourensis*.

263. Skull general shape: 0 = the skull is elevated, the skull table faces antero-dorsally or forms a dome; 1 = the skull is dorsoventrally compressed, the skull table is horizontal. Note: **0** = *Rhinochelys* morphotype '*elegans*', *Rhinochelys* morphotype '*cantabrigiensis*', specimens IRSNB GS63 & GS67; **1** = *Rhinochelys pulchriceps, Rhinochelys amaberti*; **?** = *Rhinochelys nammourensis*.

264. Orientation of the nasal cavity in dorsal view: 0 = the nasal cavity opens mainly dorsally; 1 = the nasal cavity opens mainly anteriorly. Note: **1** = all species currently assigned to *Rhinochelys*.

5 Supplementary phylogenetic results: 'Bardet full matrix'

The cladogram presented on Fig. S21 results from the maximum parsimony analysis in heuristic search analysis of the 'Bardet full matrix' in equal weighting. The dataset used comprises the entire set of taxa and characters from Bardet et al. [2013] plus the new taxa *Rhinochelys nammourensis*, *Rhinochelys pulchriceps*, *Rhinochelys amaberti*, *Rhinochelys* morphotype '*cantabrigiensis*',

Rhinochelys morphotype ‘*elegans*’ and the specimens IRSNB GS63 and IRSNB GS67. There is an evident lack of resolution on the cladogram on Fig. S21 as Pan-Chelonioidea is spilt and forms a polytomy with several other clades.

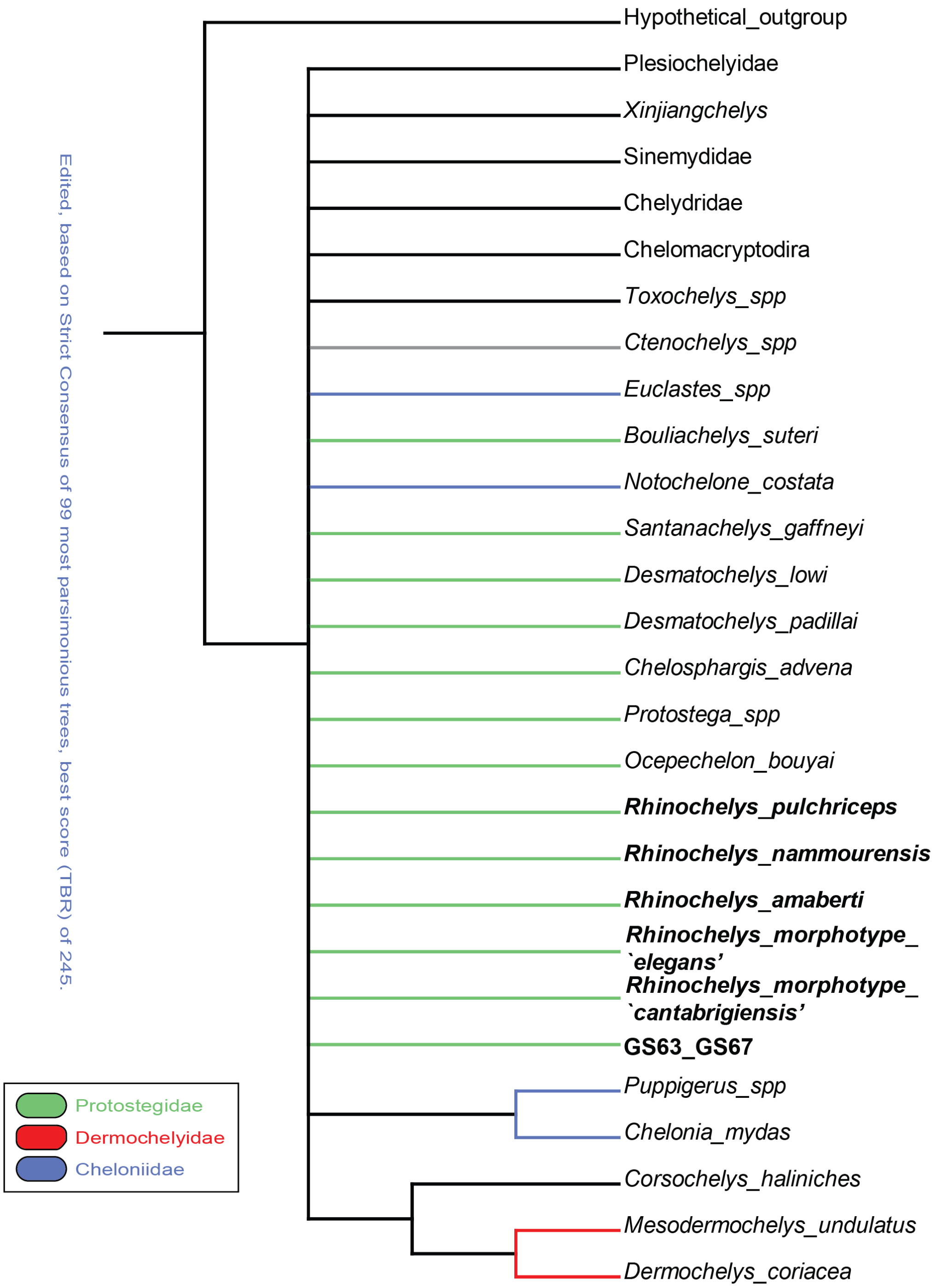

Figure S21: Strict consensus cladogram of 99 trees most parsimonious and 245 steps long, from a matrix of 104 characters and 28 taxa (‘Bardet matrix full’). In bold are the taxa we added to the dataset.

6 Cladogenesis rate

On Fig. S22 is presented the cladogenesis rates of both 'Bardet' and 'chelonioid' matrices in basic weight. Both dataset agree on the presence of an important cladogenesis burst during the Lower Cretaceous (with the peak culminating during the Hauterivian). The overall shape of the evolutionary radiation of chelonioids is similar to the one obtained in equal-weights, with the Cretaceous containing the majority of the radiation of Pan-Chelonioidea. In both graphs (**A** and **B**), the first stages of the Lower Cretaceous comprises the most cladogenesis burst, which corresponds to the apparition of the three major turtle families. Towards the end of the Lower Cretaceous, in the 'chelonioid dataset' a second intense radiating event takes place, corresponding to the radiation of dermochelyids, basal cheloniids and derived protostegids. For the 'Bardet matrix', this radiating event is split in two smaller ones. The Upper Cretaceous presents two last important events corresponding to the last radiation of derived protostegid and the apparition of Crown cheloniids. Some Pan-Chelonioidea lineages pass through the Cretaceous-Paleogene boundary, but their declining diversity never reaches the levels found during the Mesozoic.

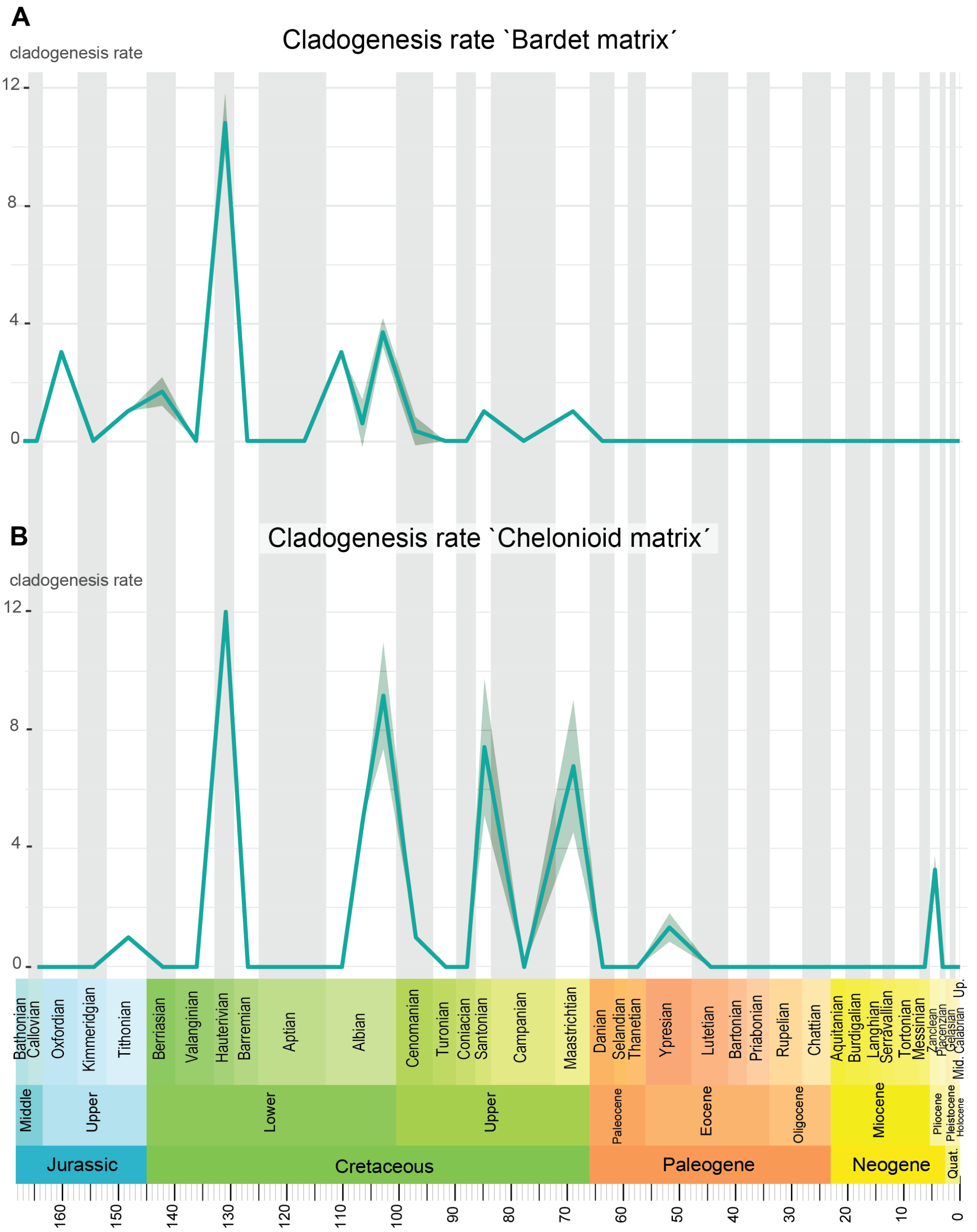

Figure S22: Mean cladogenesis rates and standard deviation using all most parsimonious trees arising from the analyses of **A** the reduced 'Bardet matrix', and **B** 'chelonioid matrix' using a 'basic' optimization of branch lengths.

References

D. W. Bapst. Paleotree: an R package for paleontological and phylogenetic analyses of evolution. *Methods in Ecology and Evolution*, 3(5):803–807, 2012. doi: 10.1111/j.2041-210X.2012.00223.x.

N. Bardet, N.-E. Jalil, F. de Lapparent de Broin, D. Germain, O. Lambert, and M. Amaghzaz. A giant chelonioid turtle from the Late Cretaceous of Morocco with a suction feeding apparatus unique among tetrapods. *PLoS ONE*, 8(7):1–10, 2013. ISSN 1932-6203. doi: 10.1371/journal.pone.0063586.

D. B. Brinkman, M. C. Aquillon-Martinez, C. A. De Leon Davila, H. Jamniczky, D. A. Eberth, and M. Colbert. *Euclastes coahuilaensis* sp. nov., a basal cheloniid turtle from the late Campanian Cerro del Pueblo Formation of Coahuila State, Mexico. *PaleoBios*, 28:76–88, 2009.

E. A. Cadena and J. F. Parham. Oldest known marine turtle? A new protostegid from the Lower Cretaceous of Colombia. *PaleoBios*, 32:1–42, 2015. URL `http://escholarship.org/uc/item/147611bv`.

J. I. Collins. The chelonian *Rhinochelys* Seeley from the Upper Cretaceous of England and France. *Palaeontology*, 13(3):355–378, 1970.

E. Cope. On *Euclastes*, a genus of extinct Cheloniidae. *Proceedings of the National Academy of Sciences of Philadelphia*, 41, 1867.

I. G. Danilov, A. O. Averianov, and A. A. Yarkov. *Itilochelys rasstrigin* gen. et sp. nov, a new hard-shelled sea turtle (Cheloniidae sensu lato) from the Lower Paleocene of Volgograd Province, Russia. *Proceedings of the Zoological Institute RAS*, 314(1):24–41, 2010.

F. de Lapparent de Broin, N. Bardet, M. Amaghzaz, and S. Meslouh. A strange new chelonioid turtle from the Latest Cretaceous Phosphates of Morocco. *Comptes Rendus Palevol*, 13(2): 87–95, 2014. doi: 10.1016/j.crpv.2013.07.008.

S. Duchene, A. Frey, A. Alfaro-núñez, P. H. Dutton, M. T. P. Gilbert, and P. A. Morin. Marine turtle mitogenome phylogenetics and evolution. *Molecular Phylogenetics and Evolution*, 65 (1):241–250, 2012. doi: 10.1016/j.ympev.2012.06.010.

E. S. Gaffney and P. A. Meylan. A phylogeny of turtles. In M. J. Benton, editor, *The phylogeny and classification of tetrapods*, pages 157–219. Oxford: Clarendon Press, 1988.

J. a. Grant-Mackie, J. Hill, and B. J. Gill. Two Eocene chelonioid turtles from Northland, New Zealand. *New Zealand Journal of Geology and Geophysics*, 54(2):181–194, 2011. ISSN 0028-8306. doi: Pii 9348058881̊0.1080/00288306.2010.520325.

O. P. Hay. On the group of fossil turtles known as the Amphichelydia, with remarks on the origin and relationships of the suborders, superfamilies, and families of Testudines. *Bulletin of the American Museum of Natural History*, 21:137–175, 1905.

R. Hirayama. Phylogenetic systematics of chelonioid sea turtles. *The Island Arc*, 3:270–284, 1994.

R. Hirayama. Oldest known sea turtle. *Nature*, 392:705–708, 1998. ISSN 0028-0836.

N.-E. Jalil, F. de Lapparent de Broin, N. Bardet, R. Vacant, B. Bouya, M. Amaghzaz, and S. Meslouh. *Euclastes acutirostris*, a new species of littoral turtle (Cryptodira, Cheloniidae) from the Palaeocene phosphates of Morocco (Oulad Abdoun Basin, Danian-Thanetian). *Comptes Rendus Palevol*, 8:447–459, 2009. doi: 10.1016/j.crpv.2009.03.002.

B. P. Kear and M. S. Lee. A primitive protostegid from Australia and early sea turtle evolution. *Biology Letters*, 2(1):116–119, 2006. ISSN 1744-9561. doi: 10.1098/rsbl.2005.0406.

R. Lydekker. On remains of Eocene and Mesozoic Chelonia and a tooth of (?) *Ornithopsis*. *Quarterly Journal of the Geological Society*, 45:227–246, 1889a. ISSN 0370-291X. doi: 10.1144/GSL.JGS.1889.045.01-04.16.

R. Lydekker. Catalogue of the fossil Reptilia and Amphibia in the British Museum (Natural history): Part 3, containing the order Chelonia. *British Museum (Natural History). Department of Geology.*, page 350, 1889b.

S. C. Lynch and J. F. Parham. The first report of hard-shelled sea turtles (Cheloniidae sensu lato) from the Miocene of California, including a new species (*Euclastes hutchisoni*) with unusually plesiomorphic characters. *Paleobios*, 23(3):21–35, 2003.

L. Moret. *Rhinochelys amaberti*, nouvelle espèce de tortue marine du Vraconien de la Fauge

près du Villard-de-Lans (Isère). *Bulletin de la Societe Geologique de France, 5ème série, t. V*, pages 605–619, 1935.

R. Owen. A monograph on the fossil Reptilia of the Cretaceous formations. *Monograph of the Palaeontographical society*, 5(11):1–118, 1851. doi: 10.5962/bhl.title.61855.

E. Paradis, J. Claude, and K. Strimmer. APE: analyses of phylogenetics and evolution in R language. *Bioinformatics*, 20:289–290, 2004.

J. F. Parham and N. D. Pyenson. New sea turtle from the Miocene of Peru and the iterative evolution of feeding ecomorphologies since the cretaceous. *Journal of paleontology*, 84(2): 231–247, 2010.

J. F. Parham, R. A. Otero, and M. E. Suárez. A sea turtle skull from the Cretaceous of Chile with comments on the taxonomy and biogeography of *Euclastes* (formerly *Osteopygis*). *Cretaceous Research*, 49:181–189, 2014. doi: 10.1016/j.cretres.2014.03.004.

H. Tong and R. Hirayama. A new species of *Tasbacka* (Testudines: Cryptodira: Cheloniidae) from the Paleocene of the Ouled Abdoun phosphate basin, Morocco. *Neues Jahrbuch für Geologie und Paläontologie*, 5:277–294, 2002.

H. Tong, R. Hirayama, E. Makhoul, and F. Escuillie. *Rhinochelys* (Chelonioidea, Prostostegidae) from the Late Cretaceous (Cenomanian) of Nammoura, Lebanon. *Atti della Societa Italiana di Scienze Naturali e del Museo Civico di Storia Naturale de Milano*, 147(1):113–138, 2006. ISSN 00378844.

R. E. Weems. Middle Miocene sea turtles (*Syllomus, Procolpochelys, Psephophorus*) from the Calvert Formation. *Journal of paleontology*, 48(2):278–303, 1974.

R. E. Weems. *Syllomus aegyptiacus*, a Miocene Pseudodont Sea Turtle. *Copeia*, (4):621–625, 1980. doi: 10.2307/1444438.

R. E. Weems. Paleocene turtles from the Aquia and Brightseat formations, with a discussion of their bearing on sea turtle evolution and phylogeny. *Proceedings of the Biological Society of Washington*, 101:109–145, 1988.

R. E. Weems. Paleogene chelonians from Maryland and Virginia. *PaleoBios*, 1:1–32, 2014. ISSN 1936-900X.

H. Wickham. Reshaping Data with the reshape Package. *Journal of Statistical Software*, 21 (12):1–20, 2007.

H. Wickham. ggplot2: Elegant Graphics for Data Analysis. *Springer-Verlag New York*, 2009.

TABLES

OTU	DATA SOURCE
‘GS63-GS67’	IRSNB GS63 and IRSNB GS67
Rhinochelys morphotype ‘*elegans*’*	Based on IRSNB GS64, IRSNB GS65, descriptions, pictures and drawings of Lydekker [1889a] and Collins [1970]
Rhinochelys pulchriceps	Revision based on IRSNB GS68, IRSNB GS70, descriptions, drawings and pictures of Owen [1851], Lydekker [1889a], and Collins [1970]
Rhinochelys amaberti	The holotype skull (UJF-ID.11167) and the associated mandible
Rhinochelys morphotype ‘*cantabrigiensis*’*	Description and pictures of the holotype from Lydekker [1889a] and Collins [1970]
Rhinochelys nammourensis	Descriptions, drawings and pictures of Tong et al. [2006]

Table S 1: Added or revised OTU’s in the phylogenetic datasets of Cadena and Parham [2015] and/or Bardet et al. [2013] and relevant data sources. The asterisk (*) indicates that these OTU’s are morphotypes based the listed data source alone; they do not include or represent all the specimens that have been referred to these species in the past.

OTU	DATA SOURCE
Syllomus aegyptiacus	Descriptions, pictures and drawings of Lydekker [1889b], [Weems, 1974] and [Weems, 1980]
Euclastes platyops	Descriptions, pictures and drawings of Cope [1867] and [Hay, 1905]
Euclastes acutirostris	Descriptions, pictures and drawings of Jalil et al. [2009]
Pacifichelys	Descriptions, pictures and drawings of Parham and Pyenson [2010]
Natator depressus	Descriptions, pictures and drawings of Brinkman et al. [2009]

Table S 2: Added or revised OTU's in the phylogenetic datasets of Cadena and Parham [2015] and/or Bardet et al. [2013] and their sources.
